# Catalyst-Free Formal Conjugate Addition/Aldol or Mannich Multicomponent Reactions of Mixed Aliphatic Organozinc Reagents, π-Electrophiles and Michael Acceptors

**DOI:** 10.3390/molecules28031401

**Published:** 2023-02-01

**Authors:** Marine Pinaud, Marc Presset, Erwan Le Gall

**Affiliations:** Univ Paris Est Créteil, CNRS, ICMPE, UMR 7182, 2 rue Henri Dunant, 94320 Thiais, France

**Keywords:** organozinc compounds, multicomponent reaction, uncatalyzed reaction, acrylates, π-electrophiles

## Abstract

Catalyst-free multicomponent reactions of mixed alkylzinc reagents with Michael acceptors and aldehydes, ketones or activated imines are described. Primary, secondary and tertiary alkylzinc reagents, pre-generated in acetonitrile from the corresponding iodoalkanes, were used in the process, leading to the very efficient formation of a variety of β-hydroxycarbonyl compounds. The imines showed more contrasting results, due to the direct addition of the organozinc compound to the C=N bond. Mechanistic assays involving TEMPO account for a polar instead of a radical character of the reaction.

## 1. Introduction

The synthesis of β-hydroxy or β-aminocarbonyl compounds has been the subject of a sustained development in recent decades [1,2,3,4]. These compounds indeed represent reliable building blocks due to the dense presence of functions and substituents at vicinal positions of the scaffold. Typical reaction conditions for their synthesis imply conjugate addition of an organometallic species to a Michael acceptor followed by the coupling of the resulting metalated species with a carbonyl compound [5,6,7,8,9,10,11,12] or an imine [13,14,15]. However, although transition metal-catalyzed reactions represent common features in the area, some related examples of uncatalyzed radical reactions mediated by dialkylzinc reagents have proved to constitute interesting routes to multi-substituted lactones or oxazolidinones [16,17]. In this context, multicomponent syntheses [18,19,20,21,22] of β-hydroxy or β-aminocarbonyl compounds are of current interest [23,24,25,26,27,28,29,30,31,32,33,34,35,36,37]. Indeed, as they avoid sequential addition of reagents, they enable notable experimental simplification and, therefore, represent a very relevant alternative to more classical sequenced conjugate addition/aldol or Mannich coupling sequenced domino reactions. However, while most works describe the copper-catalyzed reactions of dialkylzinc species with Michael acceptors and electrophiles, catalyst-free reactions of aliphatic mixed organozinc reagents have been scarcely documented [38]. These compounds are, however, characterized by their important functional tolerance, thereby representing very relevant synthetic intermediates [39,40]. In a pioneering work, Shono described the uncatalyzed zinc-promoted multicomponent assembly of alkyl iodides, Michael acceptors and carbonyl compounds [41]. However, only a limited number of examples were described, and the use of imines as potential electrophiles was not mentioned. Moreover, the polar or radical character of the mechanism was not discussed, likely due to the only transient in situ formation of organometallic (“anionic”) species under these Barbier-like conditions. Therefore, a comprehensive work on the subject remains desirable.

In preceding works, we reported the synthesis of β-hydroxy- and β-aminocarbonyl derivatives from aryl bromides, acrylates and carbonyl compounds or imines through cobalt-catalyzed multicomponent reactions [42,43,44]. In this contribution, we show that pre-generated mixed aliphatic organozinc reagents allow the high-yielding preparation of β-hydroxy- and β-aminocarbonyl compounds through catalyst-free formal conjugate addition/aldol or Mannich multicomponent reactions.

## 2. Results and Discussion

We began our study with the optimization of the three-component coupling between organozinc **1c**, chosen for its important reactivity, methyl acrylate (**2a**) and benzaldehyde (**3a**) (Table 1).

A first attempt with comparable amounts of the organozinc reagent **1c** and the acrylate **2a** quickly indicated that the uncatalyzed three-component coupling was possible in MeCN (Entry 1). The addition of 0.1 equiv CoBr_2_ and 0.2 equiv 2,2′-bipyridine (bpy) allowed a slight improvement of the reaction yield (Entry 2), but did not provide as satisfactory results as those obtained in similar couplings with arylzinc reagents (for which the presence of cobalt was mandatory) [43,44]. Unfortunately, the addition of TMSCl did not improve the result of the coupling (Entry 3). Similarly, raising the reaction temperature from rt to 80 °C (Entry 4) or increasing the acrylate amount to 4 equiv (Entry 5) provided comparable and deceiving results. However, a significant improvement of the reaction yield was observed when the organozinc **1c** was used in a higher amount than the acrylate **2a** (Entry 6). This observation was confirmed by using 3 equiv of the organozinc compound and 1 equiv of the acrylate (Entry 7). Indeed, in this case, a quantitative yield of the multicomponent coupling product **4caa** was obtained after 16 h stirring at ambient temperature. The effect of a temperature decrease was assessed by operating at −10 °C (Entry 8). However, very deceiving results were observed, with a significant drop in the reaction yield in conjunction with a decreased diastereoisomeric ratio. Finally, we assessed the effect of copper salts in the multicomponent reaction (Entry 9). In this case, a very good yield was still obtained, but the diastereoselectivity of the reaction was less interesting.

**Figure 1:**
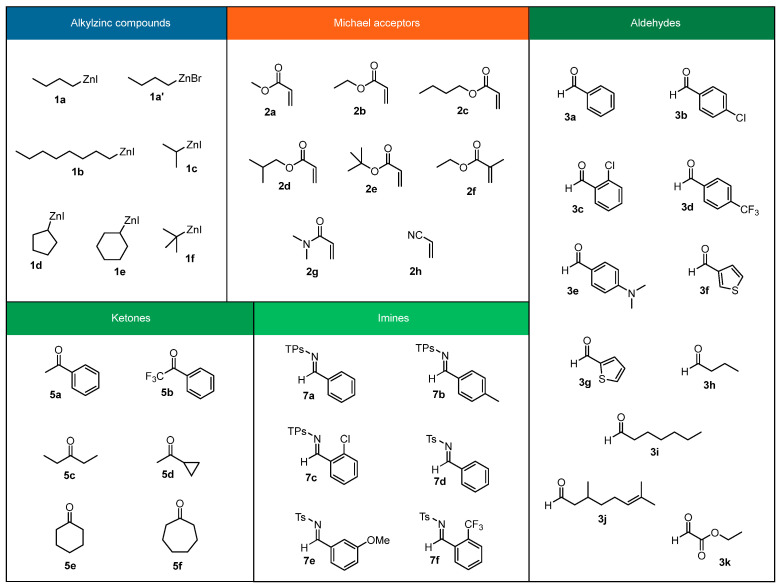
Classes of compounds used in the multicomponent reaction. Ts = toluenesulfonyl, TPs = 2-thiophenesulfonyl.

These promising results prompted us to pursue a global study in which the reactivity of mixed alkylzinc reagents would be assessed in three-component couplings with Michael acceptors and electrophiles, chosen from the pool of reagents selected for this purpose (Figure 1).

**Scheme 1:**
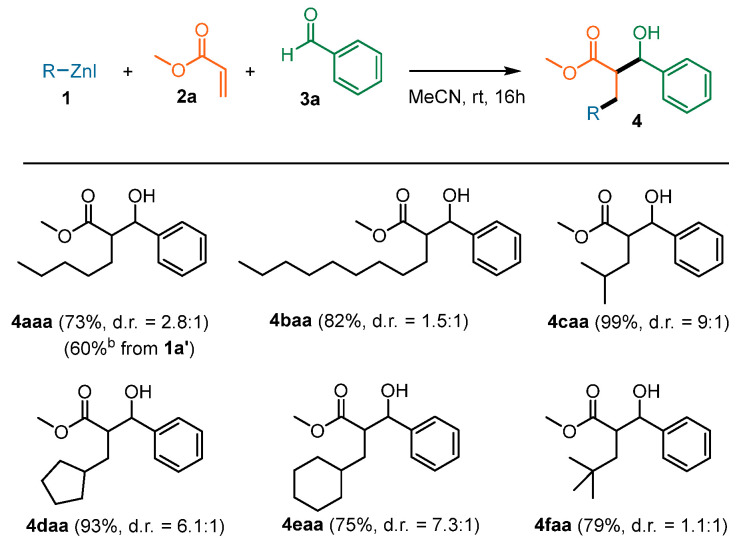
Scope of mixed alkylzinc reagents ^a^. ^a^ Yields of isolated products. Diastereoisomeric ratios (d.r.) were determined using ^1^H NMR or GC. Reaction conditions: the aldehyde, the Michael acceptor (1 equiv) and the organozinc compound (3 equiv) were stirred in MeCN for 16 h at ambient temperature. ^b^ Average GC yield over three attempts.

The reaction was first extended to a range of organozinc reagents **1**, readily prepared in acetonitrile from the corresponding alkyl iodides using a procedure already described in a recent paper from our group [45] (Scheme 1).

All the organozinc compounds gave good to excellent results, with the coupling products being obtained in 73–99% yield. The primary organozinc reagents **1a** and **1b**, obtained from the corresponding iodides, gave good results (73% and 82%, products **4aaa** and **4baa**). It can be noted that **1a’**, the brominated counterpart of **1a**, could be prepared in THF from the corresponding bromide in the presence of LiCl [46] and proved reactive in the three-component coupling, but only after the addition of acetonitrile. However, in this case, very variable yields were obtained for **4aaa** (30 to 99% GC yield, 60% average over three attempts). The reactivity of secondary organozinc compounds proved to be very convincing, with yields of the coupling products ranging from 75% (organozinc **1e**) to 99% (organozinc **1c**). Finally, we were pleased to notice that a tertiary organozinc compound, **1f**, was also usable in the coupling to furnish product **4faa** in very good yield (79%).

**Scheme 2:**
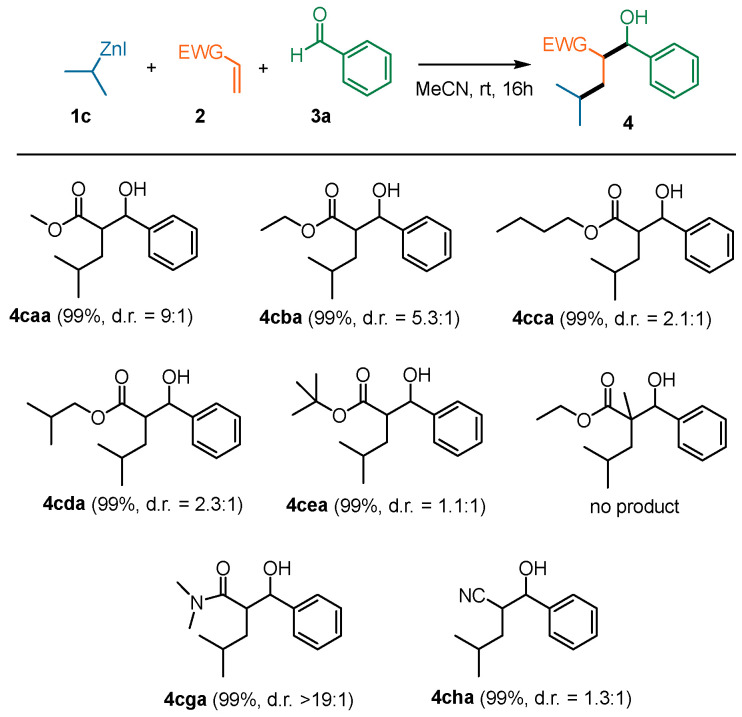
Scope of Michael acceptors ^a^. ^a^ Yields of isolated products. Diastereoisomeric ratios (d.r.) were determined using ^1^H NMR or GC. Reaction conditions: the aldehyde, the Michael acceptor (1 equiv) and the organozinc compound (3 equiv) were stirred in MeCN for 16 h at ambient temperature.

Given the results presented above, isopropylzinc iodide (**1c**) was used as the model organozinc compound for the rest of the study. Next, a range of Michael acceptors was assessed in the process (Scheme 2).

All standard acrylates **2a–e** worked very well in the three-component reaction, furnishing the corresponding coupling products in quantitative yield and confirming the predictable limited effect of the ester group on the reaction outcome. Unfortunately, ethyl methacrylate (**2f**) did not undergo the reaction. However, *N*,*N*-dimethylacrylamide (**2g**) gave very significant results, both in terms of diastereoselectivity (d.r. > 19:1) and yield (99%, product **4cga**). Finally, acrylonitrile (**2i**) worked very well and furnished the coupling product **4cia** in almost quantitative yield (99%), albeit with very limited diastereoselectivity.

**Scheme 3:**
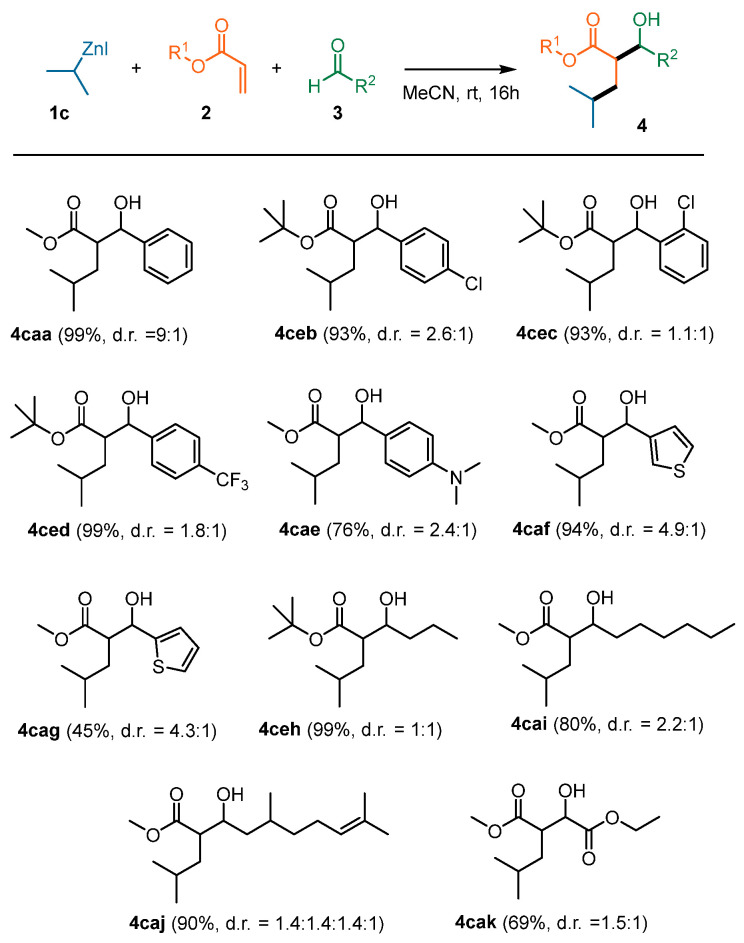
Scope of aldehydes ^a^. ^a^ Yields of isolated products. Diastereoisomeric ratios (d.r.) were determined using ^1^H NMR or GC. Reaction conditions: the aldehyde, the Michael acceptor (1 equiv) and the organozinc compound (3 equiv) were stirred in MeCN for 16 h at ambient temperature.

The reactivity of aldehydes was then evaluated in the three-component coupling (Scheme 3).

Aromatic aldehydes **3a**–**e** worked very well in the coupling. Even an *ortho*-substituted benzaldehyde **3c** gave the corresponding coupling product **4cec** in high yield (93%). The reaction could be efficiently extended to heteroaromatic aldehydes such as 3- (**3f**) or 2-thiophenecarboxaldehyde (**3g**), furnishing the coupling products **4caf** and **4cag** in moderate to high yield. Finally, aliphatic aldehydes **3h–j** were also very efficient in the multicomponent reaction and gave rise to the formation of the products **4ceh**, **4cai** and **4caj** in good to excellent yields (80–99%). It can be noted that a more original attempt using ethyl glyoxylate **3k** led to the corresponding coupling product **4cak** in good yield (69%).

**Scheme 4:**
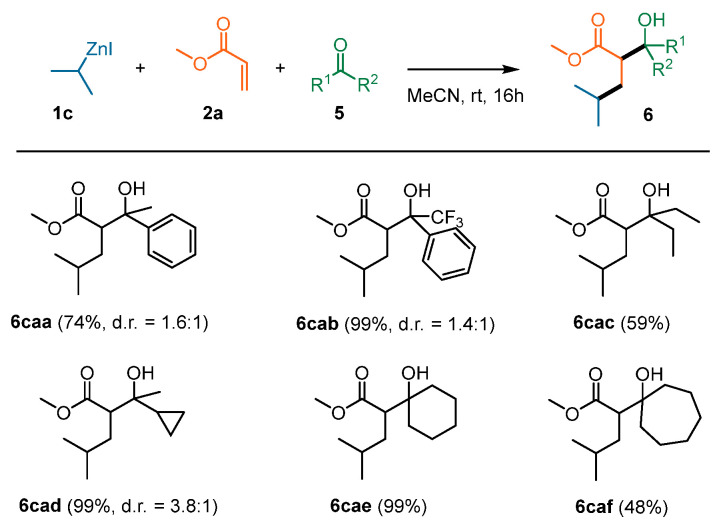
Scope of ketones ^a^. ^a^ Yields of isolated products. Diastereoisomeric ratios (d.r.) were determined using ^1^H NMR or GC. Reaction conditions: the ketone, the Michael acceptor (1 equiv) and the organozinc compound (3 equiv) were stirred in MeCN for 16 h at ambient temperature.

Next, reactions employing ketones were examined (Scheme 4).

Ketones proved usable in the three-component coupling but provided contrasting results. Indeed, while aromatic ketones gave rather good yields (74% and 99% for products **6caa** and **6cab,** respectively), reactions with some aliphatic ketones such as pentan-3-one (**5c**) or alicyclic ketones such as cycloheptanone (**5f**) gave more limited yields. However, coupling products were obtained in almost quantitative yield starting from cyclopropyl methyl ketone (**5d**) and cyclohexanone (**5e**).

**Scheme 5:**
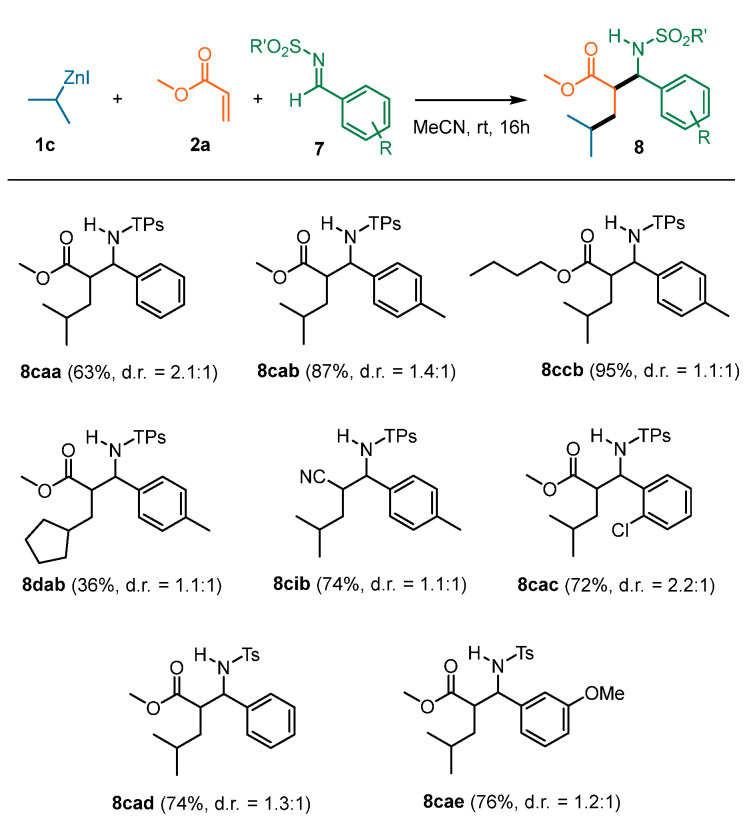
Scope of imines ^a^. ^a^ Yields of isolated products. Diastereoisomeric ratios (d.r.) were determined using ^1^H NMR or GC. Reaction conditions: the imine, the Michael acceptor (2 equiv) and the organozinc compound (3 equiv) were stirred in MeCN for 16 h at ambient temperature. TPs = 2-thiophenesulfonyl, Ps = 2-pyridinesulfonyl.

Next, the reactivity of sulfonylimines was assessed (Scheme 5).

**Scheme 6:**
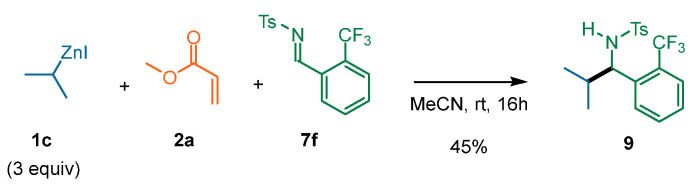
Reaction with *ortho*-trifluoromethylated imine **7f**.

In a general manner, reactions with imines were less efficient than those with carbonyl compounds. Indeed, in this case, most three-component coupling products were formed along with α-branched benzylamides, arising from the direct addition of the organozinc compound to the imine. As the probable consequence of more selective additions, the best product yields were obtained with 2-thiophenesulfonyl imines (products **8caa**–**8caf**). Interestingly, as the 2-thiophenesulfonyl activating group is easily cleaved under reductive conditions [47,48], this multicomponent reaction could represent an interesting alternative to existing methods in the case that a further regeneration of the primary amine is envisioned. It can be noted that the problem of chemoselectivity was particularly pronounced in the case of tosylimine **7f**. Indeed, in this case, and despite the presence of the Michael acceptor, the multicomponent product was only observed as traces, whereas the direct addition product **9** proved predominant (Scheme 6).

**Scheme 7:**
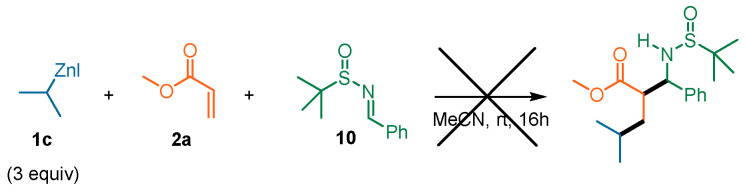
Reaction with Ellman-type sulfinylimine.

Given these original results on sulfonylimines, a sulfinylimine, the corresponding Ellman-type sulfinylimine **10**, was also evaluated in the reaction. This would additionally be a chance to assess the stereochemical outcome of the multicomponent coupling. Unfortunately, this Michael acceptor did not undergo the reaction (Scheme 7).

**Scheme 8:**
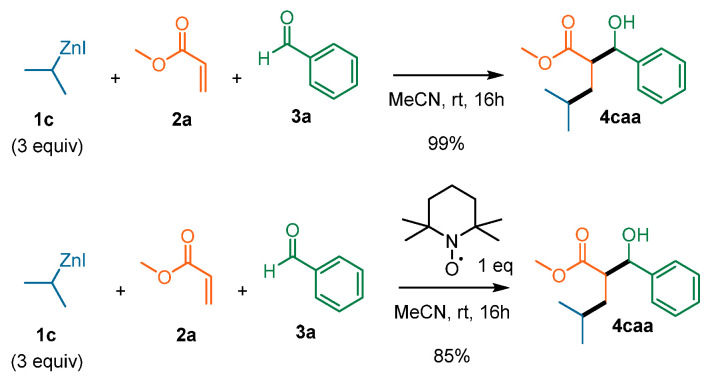
Comparative reactions in the presence and absence of TEMPO.

Insight into the reaction mechanism could be obtained by the comparison of reactions conducted in the absence or presence of 2,2,6,6-tetramethylpiperidin 1-oxyl (TEMPO), known as radical’s trap [49]. Years ago, Takai and coworkers described similar Mn-mediated reactions that were presumed to occur via the initial generation of an alkyl radical that adds to the Michael acceptor [50,51]. In our case, similar results were observed in the presence or absence of TEMPO, thus suggesting that the reaction does not involve radicals (Scheme 8).

**Scheme 9:**
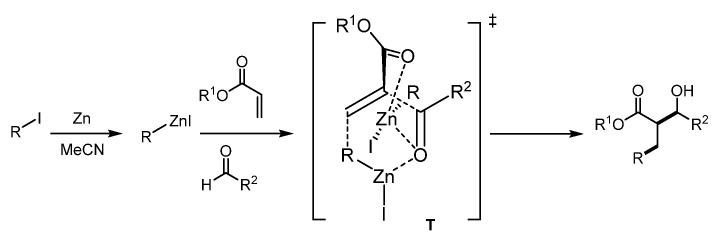
Possible reaction mechanism.

In addition, as the organozinc species are generated and titrated before multicomponent coupling with the other partners, and because an important amount of organozinc is crucial for efficiency, we presume that the reaction rather occurs via an anionic (polar) mechanism that involves the presence of a second organozinc compound, as Lewis acid, at the stage of the transition state **T** (Scheme 9).

Such a mechanism is additionally supported by the results observed when the reaction is conducted in the absence of the π-electrophile. Indeed, after the reaction of a mixed alkylzinc reagent with an acrylate, the assumed enolate intermediate is unable to trap an aldehyde sequentially added to the reaction mixture, thus indicating that all the reagents must be simultaneously present in the reaction mixture to induce the reaction.

## 3. Materials and Methods

All commercially available reagents, including solvents, were used as received.

Room temperature means 18–25 °C.

Melting points (mp) are uncorrected and were measured on a Büchi B-545 apparatus.

Analytical thin layer chromatography (TLC) was performed on TLC silica gel plates (0.25 mm) precoated with a fluorescent indicator. Visualization was effected using ultraviolet light (λ = 254 nm) and/or an aqueous solution of KMnO_4_. Flash chromatography (FC) was performed on 40–63 μm silica gel with mixtures of solvents.

High-resolution mass spectra were obtained at the ICOA of the Université of Orléans through electrospray ionization using a Q-TOF analyzer.

NMR spectra were recorded on a Bruker Avance II 400 MHz spectrometer. ^1^H NMR chemical shifts were referenced to the residual solvent signal; ^13^C NMR chemical shifts were referenced to the deuterated solvent signal. Multiplicity was defined through DEPT 135 analysis. Data are presented as follows: chemical shift δ (ppm), multiplicity (s = singlet, d = doublet, t = triplet, q = quartet, m = multiplet, br = broad), coupling constant J (Hz), integration.

d.r. were determined using GC or ^1^H NMR analysis of the crude reaction mixture.

Compound characterization data and NMR spectra for all compounds are provided in the Supplementary Materials.

**3CR with carbonyl compounds**

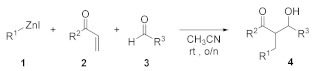



**General procedure 1 (GP1):** A 10 mL sealable tube equipped with a stir bar and filled with an argon atmosphere was charged with aldehyde **3** (0.5 mmol, 1.0 equiv), acrylate **2** (1.0 equiv) and acetonitrile (2 mL). Under stirring, a solution of organozinc reagent **1** in CH_3_CN (3.0 equiv) was added in one portion. The reaction was stirred at room temperature overnight. Then, the reaction mixture was poured into sat aq NH_4_Cl (20 mL). The resulting solution was extracted twice with EtOAc (15 + 15 mL), and the combined organic layers were washed with brine, dried over anhydrous Na_2_SO_4_ and evaporated. The resulting crude material was purified on silica gel to give product **4**.

**3CR with sulfonyl imines**

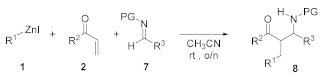



**General procedure 2 (GP2):** A 10 mL sealable tube equipped with a stir bar and filled with an argon atmosphere was charged with imine **7** (0.4 mmol, 1.0 equiv), acrylate **2** (2.0 equiv) and acetonitrile (2 mL). Under stirring, a solution of organozinc reagent **1** in CH_3_CN (3.0 equiv) was added in one portion. The reaction was stirred at room temperature overnight. Then, the reaction mixture was poured into sat aq NH_4_Cl (20 mL). The resulting solution was extracted twice with EtOAc (15 + 15 mL), and the combined organic layers were washed with brine, dried over anhydrous Na_2_SO_4_ and evaporated. The resulting crude material was purified on silica gel to give product **7**.

## 4. Conclusions

In conclusion, we show that mixed aliphatic organozinc reagents can constitute very relevant nucleophiles in uncatalyzed multicomponent reactions with Michael acceptors and π-electrophiles such as aldehydes, ketones or activated imines. Yields are generally very high and the range of compounds usable in the three-component coupling is rather important. However, reactions involving activated imines are less efficient due to the possible direct addition of the organozinc compound to the C=N bond. Comparison between reactions conducted in the presence or absence of TEMPO accounts for a polar character of the reaction mechanism instead of a radical coupling.

## Figures and Tables

**Table 1:**
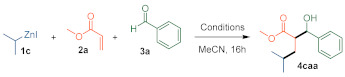
Optimization of the reaction parameters ^a^.

Entry	T (°C)	Additive	1c Equiv	2a Equiv	Yield (%)	d.r.
1	20	-	1.5	2	16%	6:04
2	20	CoBr_2_.bpy_2_ 0.1 eq	1.5	2	25%	
3	20	TMSCl 1 eq	1.5	2	6%	9:01
4	80	-	1.5	2	16%	4:01
5	20	-	1.5	4	18%	8:02
6	20	-	3	2	68%	8:02
7	20	-	3	1	99%	9:01
8	−10	-	3	1	27%	6:04
9	20	CuI	3	1	81%	8:02

^a^ Yields of isolated products. Diastereoisomeric ratios (d.r.) were determined using ^1^H NMR. Reaction conditions: the aldehyde (1 equiv), the Michael acceptor and the organozinc compound were stirred in MeCN for 16 h.

## Data Availability

Data is contained within the article or Supplementary Materials.

## Supplementary Materials

The following supporting information can be downloaded at: https://www.mdpi.com/article/10.3390/molecules28031401/s1. Experimental procedures, compound characterization data and NMR spectra for all compounds.

## Sample Availability

Samples of the compoundsare not available from the authors.
