# Supplementary material for: Catalyst-Free Formal Conjugate Addition/Aldol or Mannich Multicomponent Reactions of Mixed Aliphatic Organozinc Reagents, π-Electrophiles and Michael Acceptors
Source: Molecules. 2023 Feb 1;28(3):1401. doi: 10.3390/molecules28031401 (PMC9919058; doi:10.3390/molecules28031401)
Supplement: Supplementary file 1 [file molecules-28-01401-s001.zip › molecules-2163610-supplementary.pdf]

molecules

Catalyst-Free Formal Conjugate Addition/Aldol or Mannich Multicomponent Reactions of Mixed Aliphatic Organozinc Reagents, π-Electrophiles and Michael Acceptors

Marine Pinaud, Marc Presset * and Erwan Le Gall *

Univ Paris Est Créteil, CNRS, ICMPE, UMR 7182, 2 rue Henri Dunant, 94320 Thiais, France
* Correspondence: marc.presset@u-pec.fr (M.P.); erwan.le-gall@cnrs.fr (E.L.G.)

Table of contents

1. General information S2

2. 3CR with carbonyl compounds S2

3. 3CR with sulfonyl imines S16

4. Copies of NMR spectra S20

1. General information

All commercially available reagents, including solvents, were used as received.

Room temperature means 18–25 °C.

Melting points (mp) are uncorrected and were measured on a Büchi B-545 apparatus.

Analytical thin layer chromatography (TLC) was performed on TLC silica gel plates (0.25 mm) precoated with a fluorescent indicator. Visualization was effected using ultraviolet light (λ = 254 nm) and/or a aqueous solution of $KMnO_4$. Flash chromatography (FC) was performed on 40–63 μm silica gel with mixtures of solvents.

High-Resolution Mass Spectra were obtained at the ICOA of the Université of Orléans by electrospray ionization using a Q-TOF analyzer.

NMR spectra were recorded on a Bruker Avance II 400 MHz spectrometer. ^{1}H NMR chemical shifts were referenced to the residual solvent signal; ^{13}C NMR chemical shifts were referenced to the deuterated solvent signal. Multiplicity was defined by DEPT 135 analysis. Data are presented as follows: chemical shift δ (ppm), multiplicity (s = singlet, d = doublet, t = triplet, q = quartet, m = multiplet, br = broad), coupling constant *J* (Hz), integration.

d.r. were determined by GC or ^{1}H NMR analysis of the crude reaction mixture.

2.3. CR with carbonyl compounds

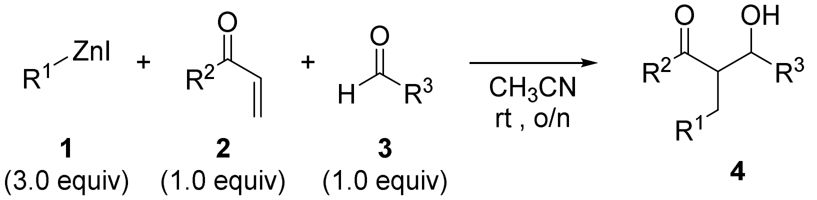

General procedure 1 (GP1): A 10 mL sealable tube equipped with a stir bar and filled with an argon atmosphere was charged with aldehyde **3** (0.5 mmol, 1.0 equiv), acrylate **2** (1.0 equiv) and acetonitrile (2 mL). Under stirring, a solution of organozinc reagent **1** in CH_3CN (3.0 equiv) was added in one portion. The reaction was stirred at room temperature overnight. Then, the reaction mixture was poured into sat aq NH_4Cl (20 mL). The resulting solution was extracted twice with EtOAc (15+15 mL) and the combined organic layers were washed brine, dried over anhydrous Na_2SO_4 and evaporated. The resulting crude material was purified on silica gel to give product **4**.

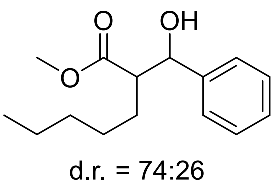

d.r. = 74:26
$C_{15}H_{22}O_3$
MW = 250,34

Compound **4aaa**: Following **GP1** performed with benzaldehyde (47 μL, 0.46 mmol, 1.0 equiv), methyl acrylate (41 μL, 0.46 mmol, 1.0 equiv) and organozinc reagent **1a** (3.0 mL, C = 0.46 M, 1.3 mmol, 3.0 equiv), the desired product was obtained after purification by FC [V(SiO_2) = 20 mL, Cy/AE: 95:5 (100 mL), 90:10 (100 mL)] as a colorless oil (84 mg, 73%, d.r: 74:26).

R*f* (Cy/AE: 95/5, UV+*p*-Anis): 0.37.

HRMS (ESI$^+$): m/z [M+Na]$^+$ Calcd. for $C_{15}H_{22}NaO_3$: 273.1461. Found: 273.1467.

^{1}H NMR (400 MHz, $CDCl_3$): δ 7.43–7.22 (m, 10H), 4.96 (d, *J* = 5.5 Hz, 2H), 3.72 (d, *J* = 1.9 Hz, 1H), 3.62 (d, *J* = 1.9 Hz, 3H), 3.60–3.56 (m, 2H), 2.92–2.69 (m, 2H), 2.13 (m, 2H), 1.86–1.53 (m, 4H), 1.52–1.15 (m, 12H), 0.99–0.75 (m, 6H).

$^{13}C\{^1H\}$ NMR (100 MHz, $CDCl_3$): δ 175.9 (C), 175.6 (C), 141.6 (2 C), 128.7 (2 CH), 128.3 (2 CH), 127.7 (2 CH), 126.5 (2 CH), 126.1 (2 CH), 75.5 (CH), 74.2 (CH), 53.2 (CH_3), 53.0 (CH_3), 51.7 (CH), 51.6 (CH), 31.7 (CH_2), 31.5 (CH_2), 29.9 (CH_2), 29.5 (CH_2), 27.3 (CH_2), 27.0 (CH_2), 22.5 (CH_2), 22.4 (CH_2), 14.0 (CH_3), 14.0 (CH_3).

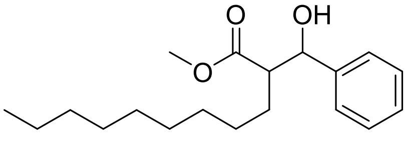

d.r. = 60:40
$C_{19}H_{30}O_3$
MW = 306,45

Compound **4baa**: Following **GP1** performed with benzaldehyde (60 µL, 0.59 mmol, 1.0 equiv), methyl acrylate (53 µL, 0.59 mmol, 1.0 equiv) and organozinc reagent **1b** (3.0 mL, C = 0.59 M, 1.8 mmol, 3.0 equiv), the desired product was obtained after purification by FC [V(SiO_2) = 20 mL, Cy/AE: 95:5 (100 mL), 90:10 (100 mL), 80:20 (100 mL)] as a colorless oil (149 mg, 82%, d.r: 60:40).

R_f (Cy/AE : 95/5, UV+*p*-Anis): 0.18.

HRMS (ESI$^+$): m/z $[M+Na]^+$ Calcd. for $C_{19}H_{30}NaO_3$: 329.2087. Found: 329.2092.

^1H NMR (400 MHz, $CDCl_3$): δ (major dia) 7.38–7.32 (m, 5H), 4.78 (d, *J* = 8.7 Hz, 1H), 3.70 (s, 3H), 3.64 (t, *J* = 6.6 Hz, 2H), 2.77–2.73 (m, 2H), 1.59–1.55 (m, 4H), 1.28–1.19 (m, 10H), 0.87 (t, *J* = 5.4 Hz, 3H).

$^{13}C\{^1H\}$ NMR (100 MHz, $CDCl_3$): δ (major dia) 176.0 (C), 142.2 (C), 128.7 (2 CH), 128.2 (CH), 126.6 (2 CH), 75.7 (CH), 53.3 (CH_3), 51.9 (CH), 32.0 (CH_2), 29.7 (CH_2), 29.6 (CH_2), 29.5 (2 CH_2), 29.4 (CH_2), 27.3 (CH_2), 22.8 (CH_2), 14.2 (CH_3).

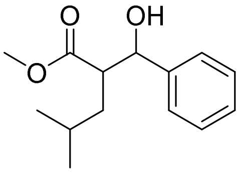

d.r. = 90:10
$C_{14}H_{20}O_3$
MW = 236,31

Compound 4caa: Following GP1 performed with benzaldehyde (49 µL, 0.48 mmol, 1.0 equiv), ethyl acrylate (43 µL, 0.48 mmol, 1.0 equiv) and organozinc reagent **1c** (4.0 mL, C = 0.36 M, 1.5 mmol, 3.0 equiv), the desired product was obtained after purification by FC [V(SiO_2) = 50 mL, Cy/AE : 95:5 (250 mL), 90:10 (250 mL)] as a yellow oil (112 mg, 99%, d.r : 90:10).

R_f (EP/AE: 95/5 , UV+$KMnO_4$): 0.34.

HRMS (ESI$^+$): m/z $[M+Na]^+$ Calcd. for $C_{14}H_{20}NaO_3$: 259.1305. Found: 259.1310.

^1H NMR (400 MHz, $CDCl_3$): δ 7.47–7.26 (m, 10H), 4.93 (d, *J* = 5.2 Hz, 2H), 3.70 (s, 1H), 3.59 (s, 5H), 2.93 (s, 2H), 2.81 (d, *J* = 5.4 Hz, 2H), 1.76 (t, *J* = 10.2 Hz, 3H), 1.43 (d, *J* = 7.6 Hz, 4H), 0.88 (d, *J* = 4.6 Hz, 5H), 0.82 (d, *J* = 4.5 Hz, 6H).

$^{13}C\{^1H\}$ NMR (100 MHz, $CDCl_3$): δ 176.1 (C), 175.7 (C), 142.1 (C), 141.6 (C), 128.6 (2 CH), 128.3 (2 CH), 128.1 (CH), 127.7 (CH), 126.5 (2 CH), 126.1 (2 CH), 76.0 (CH), 74.5 (CH), 51.7 (2 CH_3), 51.3 (CH), 51.2 (CH), 38.6 (CH_2), 36.1 (CH_2), 26.4 (CH), 26.1 (CH), 23.5 (CH_3), 23.3 (CH_3), 21.5 (CH_3), 21.4 (CH_3).

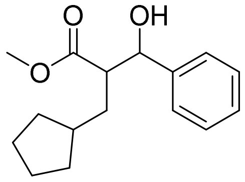

d.r. = 86:14
$C_{16}H_{22}O_3$
MW = 262,35

Compound **4daa**: Following **GP1** performed with benzaldehyde (78 μL, 0.77 mmol, 1.0 equiv), methyl acrylate (70 μL, 0.77 mmol, 1.0 equiv) and organozinc reagent **1d** (8.0 mL, C = 0.29 M, 2.3 mmol, 3.0 equiv), the desired product was obtained after purification by FC [V(SiO_2) = 50 mL, Cy/AE : 90:10 (250 mL), 80:20 (125 mL)] as a yellow oil (187 mg, 93%, d.r: 86:14).

R*f* (Cy/AE: 95/5 , UV+*p*-Anis): 0.18.

HRMS (ESI$^+$): m/z $[M+Na]^+$ Calcd. for $C_{16}H_{22}NaO_3$: 285.1461. Found: 285.1466.

^{1}H NMR (400 MHz, $CDCl_3$): δ 7.25 (m, 10H), 4.83 (d, *J* = 4.9 Hz, 2H), 3.50 (s, 6H), 2.84 (t, *J* = 2.2 Hz, 2H), 2.77–2.63 (m, 2H), 1.78 (m, 3H), 1.68–1.49 (m, 8H), 1.49–1.31 (m, 7H), 1.00–0.76 (m, 4H).

^{13}C{^{1}H} NMR (100 MHz, $CDCl_3$): δ (major dia) 175.8 (C), 141.6 (C), 128.6 (2 CH), 127.7 (CH), 126.1 (2 CH), 74.4 (CH), 52.5 (CH_3), 51.7 (CH), 38.3 (CH), 33.4 (CH_2), 33.2 (CH_2), 32.0 (CH_2), 25.1 (CH_2), 25.0 (CH_2).

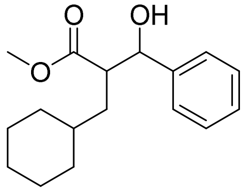

d.r. = 88:12
$C_{17}H_{24}O_3$
MW = 276,38

Compound **4eaa**: Following **GP1** performed with benzaldehyde (62 μL, 0.61 mmol, 1.0 equiv), methyl acrylate (55 μL, 0.61 mmol, 1.0 equiv) and organozinc reagent **1e** (8.0 mL, C = 0.23 M, 1.8 mmol, 3.0 equiv), the desired product was obtained after purification by FC [V(SiO_2) = 50 mL, Cy/AE : 90:10 (250 mL), 80:20 (250 mL)] as a colorless solid (126 mg, 75%, d.r: 88:12).

R*f* (Cy/AE: 95/5 , UV+*p*-Anis): 0.20.

HRMS (ESI$^+$): m/z $[M+Na]^+$ Calcd. for $C_{17}H_{24}NaO_3$: 299.162. Found: 299.1622.

mp: 66 °C

^{1}H NMR (400 MHz, $CDCl_3$): δ 7.46–7.22 (m, 10H), 4.90 (d, *J* = 5.7 Hz, 2H), 3.57 (m, 6H), 3.02 (s, 2H), 2.93–2.81 (m, 2H), 1.79–1.53 (m, 12H), 1.53–1.40 (m, 2H), 1.11 (m, 8H), 0.97–0.81 (m, 2H), 0.81–0.58 (m, 2H).

^{13}C{^{1}H} NMR (100 MHz, $CDCl_3$): δ (major dia) 175.7 (C), 141.7 (C), 128.3 (2 CH), 127.7 (CH), 126.1 (2 CH), 74.6 (CH), 51.6 (CH_3), 50.6 (CH), 35.8 (CH), 34.6 (CH_2), 34.0 (CH_2), 32.2 (CH_2), 26.5 (CH_2), 26.3 (CH_2), 26.1 (CH_2).

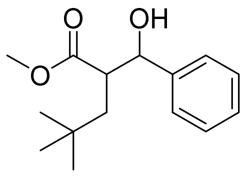

d.r. = 53:47
$C_{15}H_{22}O_3$
MW = 250,34

Compound **4faa**: Following **GP1** performed with benzaldehyde (0.17 mL, 1.6 mmol, 1.0 equiv), methyl acrylate (0.14 mL, 1.6 mmol, 1.0 equiv) and organozinc reagent **1f** (8.0 mL, C = 0.61 M, 4.9 mmol, 3.0 equiv), the desired product was obtained after purification by FC [V(SiO_2) = 50 mL, Cy/AE: 90:10 (250 mL), 80:20 (125 mL)] as a yellow oil (0.317 g, 79%, d.r: 53:47).

R_f (Cy/AE: 95/5 , UV+*p*-Anis): 0.20.

HRMS (ESI$^+$): m/z $[M+H]^+$ Calcd. for $C_{15}H_{22}NaO_3$: 273.1461. Found: 273.1461.

^{1}H NMR (400 MHz, $CDCl_3$): δ 7.33 (m, 10H), 4.81 (d, *J* = 5.7, 1H), 4.64 (d, *J* = 8.3 Hz, 1H), 3.65 (s, 3H), 3.50 (s, 3H), 2.99 (m, 2H), 2.91–2.81 (m, 1H), 2.81–2.72 (m, 1H), 1.89–1.72 (m, 2H), 1.63 (d, *J* = 2.0 Hz, 1H), 1.13 (m, 1H), 0.78 (m, 18H).

$^{13}C\{^1H\}$ NMR (100 MHz, $CDCl_3$): δ 176.8 (C), 176.2 (C), 142.1 (C), 141.6 (C), 128.5 (2 CH), 128.2 (2 CH), 128.1 (CH), 127.7 (CH), 126.7 (2 CH), 126.2 (2 CH), 77.0 (CH), 75.0 (CH), 51.8 (CH_3), 51.7 (CH_3), 49.9 (CH), 49.7 (CH), 43.0 (CH_2), 40.4 (CH_2), 30.4 (C), 30.2 (C), 29.2 (3 CH_3), 29.2 (3 CH_3).

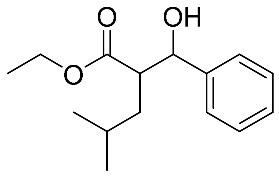

d.r. = 84:16
$C_{15}H_{22}O_3$
MW = 250,34

Compound **4cba**: Following **GP1** performed with benzaldehyde (27 µL, 0.27 mmol, 1.0 equiv), ethyl acrylate (29 µL, 0.27 mmol, 1.0 equiv) and organozinc reagent **1c** (4.0 mL, C = 0.20 M, 0.8 mmol, 3.0 equiv), the desired product was obtained after purification by FC [V(SiO_2) = 50 mL, Cy/AE: 95:5 (250 mL), 80:20 (250 mL)] as a colorless oil (67 mg, 99%, d.r: 84:16).

R_f (EP/AE : 95/5 , UV+$KMnO_4$): 0.30.

HRMS (ESI$^+$): m/z $[M+Na]^+$ Calcd. for $C_{15}H_{22}NaO_3$: 273.1461. Found: 273.1463.

^{1}H NMR (400 MHz, $CDCl_3$): δ 7.42–7.26 (m, 10H), 4.94–4.88 (m, 2H), 4.76 (t, J = 11.9, 5.9 Hz, 2H), 4.21–4.11 (m, 2H), 4.04 (m, 2H), 3.05–2.92 (m, 2H), 2.92–2.77 (m, 2H), 1.76 (t, J = 11.2 Hz, 2H), 1.53–1.40 (m, 2H), 1.22 (m, 2H), 1.12 (m, 4H), 0.88 (m, 4H), 0.87–0.82 (m, 8H).

$^{13}C\{^1H\}$ NMR (100 MHz, $CDCl_3$): δ 175.6 (C) , 175.2 (C), 142.2 (C), 141.6 (C), 128.5 (2 CH), 128.3 (2 CH), 127.9 (CH), 127.7 (CH), 126.4 (2 CH), 126.2 (2 CH), 75.8 (CH), 74.6 (CH), 60.6 (CH_2), 60.5 (CH_2), 51.3 (CH), 51.2 (CH), 38.7 (CH_2), 36.2 (CH_2), 26.4 (CH), 26.1 (CH), 23.6 (CH_3), 23.3 (CH_3), 21.6 (CH_3), 21.4 (CH_3), 14.2 (CH_3), 14.1 (CH_3).

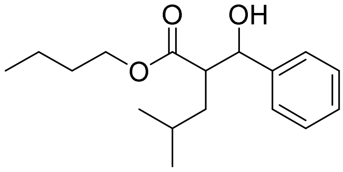

d.r. = 68:32

$C_{17}H_{26}O_3$

MW = 278,39

Compound **4cca**: Following **GP1** performed with benzaldehyde (27 μL, 0.27 mmol, 1.0 equiv), butyl acrylate (39 μL, 0.27 mmol, 1.0 equiv) and organozinc reagent **1c** (4.0 mL, C = 0.20 M, 0.8 mmol, 3.0 equiv), the desired product was obtained after purification by FC [V(SiO_2) = 50 mL, Cy/AE: 95:5 (250 mL), 80:20 (250 mL)] as a yellow oil (75 mg, 99%, d.r: 68:32).

R_f (EP/AE: 95/5 , UV+$KMnO_4$): 0.30.

HRMS (ESI$^+$): m/z $[M+Na]^+$ Calcd. for $C_{17}H_{26}NaO_3$: 301.1774. Found: 301.1775.

^{1}H NMR (400 MHz, $CDCl_3$) δ 7.44–7.26 (m, 10H), 4.93 (d, *J* = 5.9 Hz, 1H), 4.84–4.71 (m, 1H), 4.10 (t, *J* = 7.3 Hz, 3H), 4.00 (t, *J* = 6.4 Hz, 1H), 3.05–2.96 (m, 1H), 2.89 (m, 2H), 1.82–1.64 (m, 2H), 1.63–1.40 (m, 7H), 1.39–1.22 (m, 4H), 1.12 (m, 2H), 0.99–0.82 (m, 18H).

^{13}C NMR (100 MHz, $CDCl_3$) δ 175.7 (C), 175.4 (C), 142.3 (C), 141.6 (C), 128.5 (2 CH), 128.3 (2 CH), 127.9 (CH), 127.7 (CH), 126.3 (2 CH), 126.2 (2 CH), 75.8 (CH), 74.6 (CH), 64.5 (CH_2), 64.4 (CH_2), 51.3 (CH), 51.2 (CH), 38.8 (CH_2), 36.2 (CH_2), 30.6 (CH_2), 30.5 (CH_2), 26.4 (CH), 26.1 (CH), 23.6 (CH_3), 23.3 (CH_3), 21.6 (CH_3), 21.4 (CH_3), 19.0 (2 CH_2), 13.7 (2 CH_3).

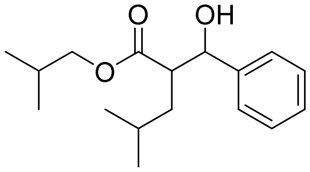

d.r. = 70:30

$C_{17}H_{26}O_3$

MW = 278,39

Compound **4cda**: Following **GP1** performed with benzaldehyde (27 μL, 0.27 mmol, 1.0 equiv), *iso*-butyl acrylate (39 μL, 0.27 mmol, 1.0 equiv) and organozinc reagent **1c** (4.0 mL, C = 0.20 M, 0.8 mmol, 3.0 equiv), the desired product was obtained after purification FC [V(SiO_2) = 50 mL, Cy/AE: 95:5 (250 mL), 80:20 (250 mL)] as a yellow oil (75 mg, 99%, d.r: 70:30).

R_f (EP/AE : 95/5 , UV+$KMnO_4$): 0.32.

HRMS (ESI$^+$): m/z $[M+Na]^+$ Calcd. for $C_{17}H_{26}NaO_3$: 301.1774. Found: 301.1776.

^{1}H NMR (400 MHz, $CDCl_3$): δ 7.43–7.27 (m, 10H), 4.92 (d, *J* = 5.4 Hz, 1H), 4.76 (t, *J* = 6.2 Hz, 1H), 3.91–3.85 (m, 2H), 3.85–3.70 (m, 2H), 3.10–2.94 (m, 2H), 2.83 (m, 2H), 1.94–1.60 (m, 5H), 1.47 (m, 4H), 0.89 (d, *J* = 4.7 Hz, 6H), 0.89–0.83 (m, 17H).

^{13}C{^{1}H} NMR (100 MHz, $CDCl_3$): δ 175.7 (C), 175.3 (C), 142.3 (C), 141.7 (C), 128.5 (2 CH), 128.3 (2 CH), 127.9 (CH), 127.7 (CH), 126.4 (2 CH), 126.2 (2 CH), 75.8 (CH), 74.6 (CH), 70.8 (CH_2), 70.7 (CH_2), 51.4 (CH), 51.3 (CH), 38.8 (CH_2), 36.3 (CH_2), 27.6 (CH), 27.5 (CH), 26.4 (CH), 26.1 (CH), 23.6 (CH_3), 23.3 (CH_3), 21.5 (CH_3), 21.4 (CH_3), 19.0 (2 CH_3), 19.0 (2 CH_3).

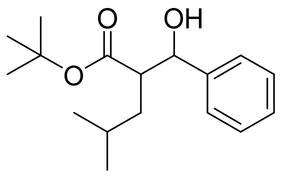

d.r. = 53:47
$C_{17}H_{26}O_3$
MW = 278,39

Compound **4cea**: Following **GP1** performed with benzaldehyde (27 µL, 0.27 mmol, 1.0 equiv), *tert*-butyl acrylate (39 µL, 0.27 mmol, 1.0 equiv) and organozinc reagent **1c** (4.0 mL, C = 0.20 M, 0.8 mmol, 3.0 equiv), the desired product was obtained after purification by FC [V(SiO_2) = 50 mL, Cy/AE: 95:5 (250 mL), 80:20 (250 mL)] as a yellow oil (75 mg, 99%, d.r: 53:47).

R*f* (EP/AE: 95/5, UV+$KMnO_4$): 0.30.

HRMS (ESI$^+$): m/z [M+Na]$^+$ Calcd. for $C_{17}H_{26}NaO_3$: 301.1774. Found: 301.1775.

^{1}H NMR (400 MHz, $CDCl_3$): δ 7.42–7.26 (m, 10H), 4.87 (m, 1H), 4.74 (m, 1H), 3.19 (d, *J* = 6.9, 1H), 2.83–2.68 (m, 2H), 1.79–1.65 (m, 2H), 1.40 (s, 13H), 1.33 (s, 10H), 0.99–0.83 (m, 12H).

^{13}C{^{1}H} NMR (100 MHz, $CDCl_3$): δ 175.1 (C), 174.6 (C), 142.5 (C), 141.7 (C), 128.3 (2 CH), 128.2 (2 CH), 127.7 (CH), 127.6 (CH), 126.5 (2 CH), 126.3 (2 CH), 81.2 (C), 81.0 (C), 75.7 (CH), 74.8 (CH), 51.7 (CH), 51.5 (CH), 39.0 (CH_2), 36.5 (CH_2), 28.0 (3 CH_3), 27.9 (3 CH_3), 26.4 (CH), 26.1 (CH), 23.7 (CH_3), 23.4 (CH_3), 21.6 (CH_3), 21.4 (CH_3).

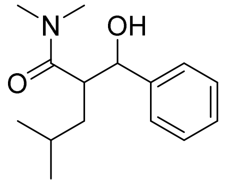

d.r. > 95:5
$C_{15}H_{23}NO_2$
MW = 249,35

Compound **4cga**: Following **GP1** performed with benzaldehyde (27 µL, 0.27 mmol, 1.0 equiv), dimethyl acrylamide (28 µL, 0.27 mmol, 1.0 equiv) and organozinc reagent **1c** (4.0 mL, C = 0.20 M, 0.8 mmol, 3.0 equiv), the desired product was obtained after purification by FC [V(SiO_2) = 50 mL, Cy/AE: 95:5 (250 mL), 90:10 (250 mL), 70:30 (125 mL), 60:40 (125 mL)] as a yellow oil (66 mg, 99%, d.r > 95:5).

R*f* (Cy/AE: 95/5 , UV+$KMnO_4$): 0.10.

HRMS (ESI$^+$): m/z [M+Na]$^+$ Calcd. for $C_{15}H_{23}NaNO_2$: 272.1621. Found: 272.1630.

^{1}H NMR (400 MHz, $CDCl_3$): δ 7.45–7.22 (m, 5H), 4.89 (d, *J* = 3.1 Hz, 1H), 3.05 (d, *J* = 10.9 Hz, 1H), 2.93 (d, *J* = 6.0 Hz, 6H), 2.00 (br s, 1H), 1.84 (t, *J* = 10.5 Hz, 1H), 1.35 (m, 2H), 0.83 (d, *J* = 4.4 Hz, 3H), 0.74 (d, *J* = 4.3 Hz, 3H).

^{13}C{^{1}H} NMR (100 MHz, $CDCl_3$): δ 175.9 (C), 142.1 (C), 128.1 (2 CH), 127.3 (CH), 125.9 (2 CH), 74.2 (CH), 46.0 (CH), 37.5 (2 CH_3), 35.7 (CH_2), 26.0 (CH), 23.6 (CH_3), 22.1 (CH_3).

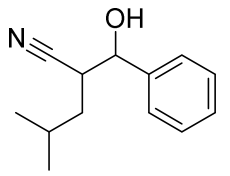

d.r. = 56:44
$C_{13}H_{17}NO$
MW = 203,29

Compound **4cha**: Following **GP1** performed with benzaldehyde (27 μL, 0.27 mmol, 1.0 equiv), acrylonitrile (18 μL, 0.27 mmol, 1.0 equiv) and organozinc reagent **1c** (4.0 mL, C = 0.20 M, 0.8 mmol, 3.0 equiv), the desired product was obtained after purification by FC [V(SiO_2) = 50 mL, Cy/AE: 95:5 (250 mL), 90:10 (250 mL), 70:30 (125 mL)] as a **pale yellow oil** (55 mg, 99%, d.r: 56:44).

R_f (EP/AE: 95/5, UV+$KMnO_4$): 0.31.

HRMS (ESI$^+$): m/z $[M+H]^+$ Calcd. for $C_{13}H_{18}NO$: 204.1383. Found: 204.1386.

^1H NMR (400 MHz, $CDCl_3$): δ 7.51–7.34 (m, 10H), 4.86–4.71 (m, 2H), 3.10–2.83 (m, 4H), 1.99–1.80 (m, 2H), 1.68 (m, 1H), 1.51 (m, 1H), 1.45–1.30 (m, 1H), 1.26–1.16 (m, 1H), 0.96 (m, 6H), 0.94–0.82 (m, 6H).

$^{13}C\{^1H\}$ NMR (100 MHz, $CDCl_3$): δ 140.5 (C), 140.0 (C), 128.8 (2 CH), 128.7 (2 CH) , 128.7 (2 CH), 126.5 (2 CH), 126.2 (2 CH), 120.3 (2 C), 74.2 (CH), 74.0 (CH), 39.3 (CH), 38.6 (CH), 37.7 (CH_2), 36.6 (CH_2), 26.1 (CH), 26.1 (CH), 23.3 (CH_3), 23.1 (CH_3), 21.2 (CH_3), 21.2 (CH_3).

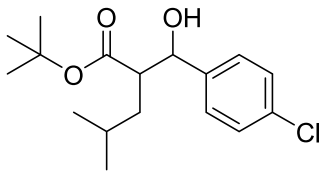

d.r. = 72:28
$C_{17}H_{25}ClO_3$
MW = 312,83

Compound **4ceb**: Following **GP1** performed with 4-chlorobenzaldehyde (51 μL, 0.43 mmol, 1.0 equiv), *tert*-butyl acrylate (62 μL, 0.43 mmol, 1.0 equiv) and organozinc reagent **1c** (4.0 mL, C = 0.32 M, 1.3 mmol, 3.0 equiv), the desired product was obtained after purification by FC [V(SiO_2) = 50 mL, Cy/AE: 95:5 (250 mL), 80:20 (250 mL)] as a colorless oil (125 mg, 93%, d.r: 72:28).

R_f (Cy/AE: 95/5, UV+$KMnO_4$): 0.34.

HRMS (ESI$^+$): m/z $[M+Na]^+$ Calcd. for $C_{17}H_{25}ClNaO_3$: 335.1384. Found: 335.1384.

^1H NMR (400 MHz, $CDCl_3$): δ 7.33–7.15 (m, 8H), 4.78 (d, *J* = 5.4 Hz, 1H), 4.60 (s, 1H), 3.24 (d, *J* = 6.0 Hz, 1H), 3.03 (s, 1H), 2.67–2.52 (m, 2H), 1.72–1.56 (m, 2H), 1.56–1.33 (m, 4H), 1.28 (m, 18H), 0.88–0.78 (m, 6H), 0.74 (d, *J* = 6.5, 6H).

$^{13}C\{^1H\}$ NMR (100 MHz, $CDCl_3$): δ 174.9 (C), 174.6 (C), 141.1 (C), 140.2 (C), 133.3 (C), 133.2 (C), 128.5 (2 CH), 128.3 (2 CH), 127.9 (2 CH), 127.7 (2 CH), 81.5 (C), 81.3 (C), 74.9 (CH), 73.9 (CH), 51.4 (CH), 51.3 (CH), 39.0 (CH_2), 36.1 (CH_2), 28.0 (3 CH_3), 27.9 (3 CH_3), 26.3 (CH), 26.1 (CH), 23.7 (CH_3), 23.3 (CH_3), 21.6 (CH_3), 21.3 (CH_3).

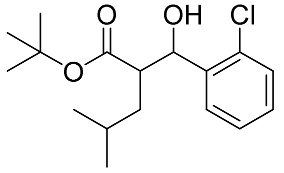

d.r. = 52:48
$C_{17}H_{25}ClO_3$
MW = 312,83

Compound **4cec**: Following **GP1** performed with 2-chlorobenzaldehyde (48 µL, 0.43 mmol, 1.0 equiv), *tert*-butyl acrylate (62 µL, 0.43 mmol, 1.0 equiv) and organozinc reagent **1c** (4.0 mL, C = 0.32 M, 1.3 mmol, 3.0 equiv), the desired product was obtained after purification by FC [V(SiO_2) = 50 mL, Cy/AE: 95:5 (250 mL), 80:20 (250 mL)] as a pale yellow oil (125 mg, 93%, d.r: 52:48).

R_f (Cy/AE: 95/5, UV+$KMnO_4$): 0.30.

HRMS (ESI$^+$): m/z $[M+Na]^+$ Calcd. for $C_{17}H_{25}ClNaO_3$: 335.1384. Found: 335.1383.

^{1}H NMR (400 MHz, $CDCl_3$): δ 7.62 (d, *J* = 7.7 Hz, 1H), 7.48 (m, 1H), 7.46–7.19 (m, 6H), 5.34 (d, *J* = 4.1, 1H), 5.14 (d, *J* = 4.3 Hz, 1H), 2.90 (m, 2H), 1.90–1.53 (m, 2H), 1.44 (m, 11H), 1.33 (m, 13H), 1.03–0.89 (m, 6H), 0.86 (d, *J* = 6.6, 3H), 0.74 (d, *J* = 6.7, 3H).

^{13}C{^{1}H} NMR (100 MHz, $CDCl_3$): δ 175.5 (C), 175.3 (C), 140.1 (2 C), 131.9 (2 C), 129.4 (CH), 129.4 (CH), 128.7 (CH), 128.6 (CH), 128.6 (CH), 127.6 (CH), 126.8 (CH), 126.7 (CH), 81.6 (C), 81.3 (C), 72.0 (CH), 71.0 (CH), 48.7 (CH), 47.8 (CH), 39.3 (CH_2), 35.0 (CH_2), 28.0 (3 CH_3), 28.0 (3 CH_3), 26.4 (CH), 26.1 (CH), 23.5 (CH_3), 23.2 (CH_3), 21.9 (CH_3), 21.3 (CH_3).

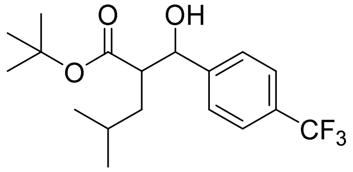

d.r. = 58:42
$C_{18}H_{25}F_3O_3$
MW = 346,39

Compound **4ced**: Following **GP1** performed with 4-trifluoromethylbenzaldehyde (59 µL, 0.43 mmol, 1.0 equiv), *tert*-butyl acrylate (62 µL, 0.43 mmol, 1.0 equiv) and organozinc reagent **1c** (4.0 mL, C = 0.32 M, 1.3 mmol, 3.0 equiv), the desired product was obtained after purification by FC [V(SiO_2) = 50 mL, Cy/AE: 95:5 (250 mL), 80:20 (250 mL)]as a yellow oil (0.149 g, 99%, d.r: 58:42).

R_f (Cy/AE: 95/5, UV+$KMnO_4$): 0.28

HRMS (ESI$^+$): m/z $[M+Na]^+$ Calcd. for $C_{18}H_{25}F_3NaO_3$: 369.1648. Found: 369.1650.

^{1}H NMR (400 MHz, $CDCl_3$): δ 7.56–7.48 (m, 4H), 7.48–7.35 (m, 4H), 4.91–4.83 (m, 1H), 4.69 (m, 1H), 3.45 (d, *J* = 7.4 Hz, 1H), 3.24–3.14 (m, 1H), 2.69–2.53 (m, 2H), 1.73–1.54 (m, 2H), 1.37 (d, *J* = 13.9 Hz, 2H), 1.26 (m, 18H), 1.20–1.09 (m, 3H), 0.82–0.76 (m, 8H), 0.72 (m, 3H).

^{13}C{^{1}H} NMR (100 MHz, $CDCl_3$): δ 174.9 (C), 174.8 (C), 146.8 (C), 145.7 (C), 129.9 (q, *J* = 39.1 Hz, C), 129.9 (q, *J* = 25.8 Hz, C), 126.9 (2 CH), 126.6 (2 CH), 125.3 (q, *J* = 3.8 Hz, 2 CH), 125.2 (q, *J* = 3.9 Hz, 2 CH), 124.3 (d, *J* = 271.8 Hz, C), 124.2 (q, *J* = 271.8 Hz, C), 81.8 (C), 81.6 (C), 75.0 (CH), 74.0 (CH), 51.3 (CH), 51.2 (CH), 39.1 (CH_2), 36.0 (CH_2), 28.0 (6 CH_3), 26.3 (CH), 26.2 (CH), 23.7 (CH_3), 23.3 (CH_3), 21.8 (CH_3), 21.3 (CH_3).

^{19}F NMR (377 MHz, $CDCl_3$): δ –62.4 (3F), –62.5 (3F).

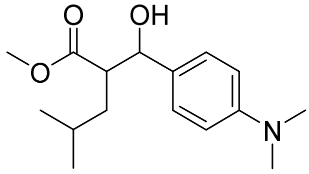

d.r. = 71:29
$C_{16}H_{25}NO_3$
MW = 279,38

Compound **4cae**: Following **GP1** performed with 4-dimethylaminobenzaldehyde (31 mg, 0.21 mmol, 1.0 equiv), methyl acrylate (19 µL, 0.21 mmol, 1.0 equiv) and organozinc reagent **1c** (3.0 mL, C = 0.21 M, 0.63 mmol, 3.0 equiv), the desired product was obtained after purification by FC [V(SiO_2) = 50 mL, Cy/AE: 95:5 (250 mL), 90:10 (125 mL), 70:30 (125 mL), 60:40 (125 mL)] as an orange oil (45 mg, 76%, d.r: 71:29).

R_f (Cy/AE: 95/5, UV+$KMnO_4$): 0.20.

HRMS (ESI$^+$): m/z [M+H]$^+$ Calcd. for $C_{16}H_{26}NO_3$: 280.1907. Found: 280.1909.

^{1}H NMR (400 MHz, $CDCl_3$): δ 7.22 (d, *J* = 1.9 Hz, 4H), 6.76–6.67 (m, 4H), 4.80 (d,J = 8.6 Hz, 1H), 4.66 (d, *J* = 8.6 Hz, 1H), 3.75 (m, 3H), 3.59–3.54 (m, 3H), 3.02–2.93 (m, 12H), 1.86–1.63 (m, 5H), 1.62–1.38 (m, 5H), 1.00–0.78 (m, 12H).

^{13}C{^{1}H} NMR (100 MHz, $CDCl_3$): δ 176.3 (C), 175.5 (C), 150.4 (C), 150.2 (C), 129.7 (C), 129.6 (C), 127.5 (2 CH), 127.1 (2 CH), 112.4 (2 CH), 112.3 (2 CH), 76.1 (CH), 74.7 (CH), 51.6 (CH_3), 51.5 (CH_3), 40.6 (CH), 40.6 (CH), 38.6 (2 CH_3), 38.3 (2 CH_3), 37.3 (CH_2), 36.9 (CH_2), 26.4 (CH), 26.2 (CH), 23.6 (CH_3), 23.4 (CH_3), 21.5 (CH_3), 21.4 (CH_3).

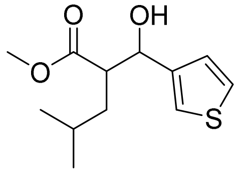

d.r. = 83:17
$C_{12}H_{18}O_3S$
MW = 242,33

Compound **4caf**: Following **GP1** performed with thiophene 3-carboxyaldehyde (18 µL, 0.21 mmol, 1.0 equiv), methyl acrylate (19 µL, 0.21 mmol, 1.0 equiv) and organozinc reagent **1c** (3.0 mL, C = 0.21 M, 0.63 mmol, 3.0 equiv), the desired product was obtained after purification by FC [V(SiO_2) = 50 mL, Cy/AE: 90:10 (250 mL), 80:20 (125 mL), 70:30 (125 mL)]as an orange oil (48 mg, 94%, d.r: 83:7)

R_f (Cy/AE: 95/5, UV+$KMnO_4$): 0.10.

HRMS (ESI$^+$): m/z [M+Na]$^+$ Calcd. for $C_{12}H_{18}NaO_3S$:265.0869. Found: 265.0872.

^{1}H NMR (400 MHz, $CDCl_3$): δ 7.40–7.27 (m, 2H), 7.27–7.18 (m, 2H), 7.08 (m, 2H), 4.84 (t, *J* = 5.8 Hz, 2H), 3.70 (s, 4H), 3.63 (m, 2H), 3.00 (d, *J* = 6.1 Hz, 2H), 2.88 (m, 2H), 1.65 (m, 2H), 1.49 (m, 2H), 1.14 (m, 2H), 0.86 (d, *J* = 6.6, 12H).

^{13}C{^{1}H} NMR (100 MHz, $CDCl_3$): δ 176.0 (C), 175.6 (C), 143.8 (C), 143.1 (C), 126.4 (CH), 126.0 (CH), 125.5 (CH), 125.5 (CH), 121.9 (CH), 121.4 (CH), 71.9 (CH), 71.4 (CH), 51.8 (CH_3), 51.7 (CH_3), 50.7 (2 CH), 38.6 (CH_2), 36.3 (CH_2), 26.3 (CH), 26.1 (CH), 23.5 (CH_3), 23.2 (CH_3), 21.6 (CH_3), 21.5 (CH_3).

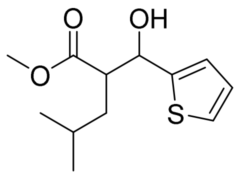

d.r. = 81:19
$C_{12}H_{18}O_3S$
MW = 242,33

Compound **4cag**: Following **GP1** performed with thiophene 2-carboxyaldehyde (18 µL, 0.21 mmol, 1.0 equiv), methyl acrylate (19 µL, 0.21 mmol, 1.0 equiv) and organozinc reagent **1c** (3.0 mL, C = 0.21 M, 0.63 mmol, 3.0 equiv), the desired product was obtained after purification by FC [V(SiO_2) = 50 mL, Cy/AE: 95:5 (250 mL), 90:10 (125 mL), 70:30 (125 mL)]as a yellow oil (23 mg, 45%, d.r: 81:19).

R_f (Cy/AE: 95/5 , UV+*p*-Anis): 0.29.

HRMS (ESI$^+$): m/z [M+Na]$^+$ Calcd. for $C_{12}H_{18}NaO_3S$: 265.0869. Found: 265.0870

^{1}H NMR (400 MHz, $CDCl_3$): δ 7.34–7.25 (m, 3H), 7.00 (d, *J* = 5.5 Hz, 3H), 5.02 (t, *J* = 7.6 Hz, 1H), 3.74 (s, 3H), 3.68 (d, *J* = 5.5 Hz, 4H), 3.11–2.86 (m, 2H), 1.58 (m, 6H), 1.41–1.12 (m, 3H), 0.89 (t, *J* = 6.1 Hz, 11H).

^{13}C{^{1}H} NMR (100 MHz, $CDCl_3$): δ 175.9 (2 C),146.4 (2 C), 126.8 (2 CH), 125.4 (2 CH), 124.9 (2 CH), 71.9 (2 CH), 52.0 (2 CH_3), 51.5 (2 CH), 38.9 (2 CH_2), 26.2 (2 CH), 23.3 (2 CH_3), 21.7 (2 CH_3).

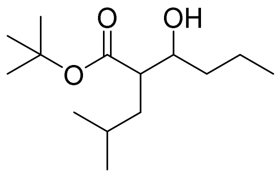

d.r. = 51:49
$C_{14}H_{28}O_3$
MW = 244,38

Compound **4ceh**: Following **GP1** performed with butyraldehyde (39 µL, 0.43 mmol, 1.0 equiv), *tert*-butyl acrylate (62 µL, 0.43 mmol, 1.0 equiv) and organozinc reagent **1c** (4.0 mL, C = 0.32 M, 1.3 mmol, 3.0 equiv), the desired product was obtained after purification by FC [V(SiO_2) = 50 mL, Cy/AE: 95:5 (250 mL), 80:20 (125 mL)]as a colorless oil (0.104 g, 99%, d.r: 51:49).

R_f (Cy/AE: 95/5, UV+$KMnO_4$): 0.25.

HRMS (ESI$^+$): m/z [M+Na]$^+$ Calcd. for $C_{14}H_{28}NaO_3$: 267.1931. Found: 267.1933.

^{1}H NMR (400 MHz, $CDCl_3$): δ 3.75–3.73 (m, 1H), 3.58–3.54 (m, 1H), 2.65–2.57 (m, 2H), 2.39 (dd, *J* = 10.2, 4.6 Hz, 2H), 1.73–1.62 (m, 4H), 1.56–1.43 (m, 22H), 1.36–1.24 (m, 6H), 0.92–0.89 (m, 18H).

^{13}C{^{1}H} NMR (100 MHz, $CDCl_3$): δ 175.5 (C), 175.3 (C), 81.1 (C), 81.0 (C), 72.8 (CH), 72.1 (CH), 49.6 (CH), 49.3 (CH), 39.0 (CH_2), 38.2 (CH_2), 36.3 (CH_2), 35.9 (CH_2), 28.1 (3 CH_3), 28.1 (3 CH_3), 26.4 (CH), 26.1 (CH), 23.6 (CH_3), 23.2 (CH_3), 21.9 (CH_3), 21.6 (CH_3), 19.1 (CH_2), 19.1 (CH_2), 14.0 (2 CH_3).

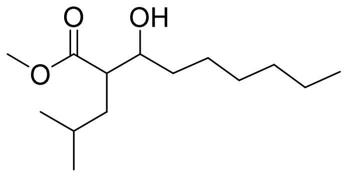

d.r. = 69:31
$C_{14}H_{28}O_3$
MW = 244,38

Compound **4cai**: Following **GP1** performed with heptanal (30 µL, 0.21 mmol, 1.0 equiv), methyl acrylate (19 µL, 0.21 mmol, 1.0 equiv) and organozinc reagent **1c** (3.0 mL, C = 0.21 M, 0.63 mmol, 3.0 equiv), the desired product was obtained after purification by FC [V(SiO_2) = 50 mL, Cy/AE: 95:5 (250 mL), 90:10 (125 mL), 80:20 (125 mL)] as a yellow oil (41 mg, 80%, d.r: 69:31).

R_f (Cy/AE: 95/5, UV+*p*-Anis): 0.16.

HRMS (ESI$^+$): m/z [M+Na]$^+$ Calcd. for $C_{14}H_{28}NaO_3$: 267.1931. Found: 267.1929.

^{1}H NMR (400 MHz, $CDCl_3$): δ 3.74–3.55 (m, 8H), 2.54–2.29 (m, 2H), 2.24 (td, *J* = 8.9, 6.8, 2.9 Hz, 1H), 1.89–1.58 (m, 3H), 1.58–1.28 (m, 8H), 1.21 (m, 16H), 0.83 (d, *J* = 7.0 Hz, 18H).

^{13}C{^{1}H} NMR (100 MHz, $CDCl_3$): δ 176.3 (C), 176.3 (C), 72.8 (CH), 72.4 (CH), 51.6 (CH_3), 51.5 (CH_3), 49.0 (CH), 48.9 (CH), 41.5 (CH_2), 38.8 (CH_2), 35.9 (CH_2), 35.9 (CH_2), 34.2 (CH_2), 31.8 (CH_2), 29.2 (CH_2), 27.6 (CH_2), 26.4 (CH), 26.2 (CH), 25.9 (CH_2), 25.7 (CH_2), 22.8 (CH_3), 22.6(2 CH_2), 22.2 (CH_3), 22.0 (CH_3), 21.7 (CH_3), 14.1 (2 CH_3).

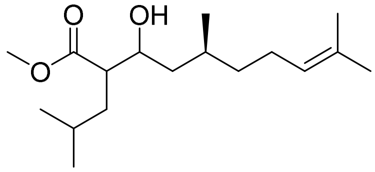

d.r. = 27:27:27:19
$C_{17}H_{32}O_3$
MW = 284,44

Compound **4caj**: Following **GP1** performed with citronellal (38 µL, 0.21 mmol, 1.0 equiv), methyl acrylate (19 µL, 0.21 mmol, 1.0 equiv) and organozinc reagent **1c** (3.0 mL, C = 0.21 M, 0.63 mmol, 3.0 equiv), the desired product was obtained after purification by FC [V(SiO_2) = 50 mL, Cy/AE: 90:10 (250 mL), 80:20 (125 mL), 70:30 (125 mL)] as a yellow oil (54 mg, 90%, d.r. : 27:27:27:19).

R_f (Cy/AE: 95/5, UV+$KMnO_4$): 0.28.

HRMS (ESI$^+$): m/z [M+H]$^+$ Calcd. for $C_{17}H_{33}O_3$: 285.2424. Found: 285.2423.

^{1}H NMR (400 MHz, $CDCl_3$): δ 5.10 (t, *J* = 7.3 Hz, 4H), 3.71 (d, *J* = 2.0 Hz, 13H), 2.53–2.39 (m, 6H), 2.11–1.82 (m, 8H), 1.64 (d, *J* = 31.3 Hz, 31H), 1.44–1.08 (m, 28H), 1.00–0.81 (m, 38H).

^{13}C{^{1}H} NMR (100 MHz, $CDCl_3$): δ 176.3 (C), 176.3 (C), 176.3 (C), 176.1 (C), 131.3 (C), 131.2 (C), 131.2 (2 C), 124.7 (4 CH), 70.9 (CH), 70.6 (CH), 70.3 (CH), 70.0 (CH), 51.6 (CH_3), 51.6 (CH_3), 51.5 (2 CH_3), 49.8 (CH), 49.7 (CH), 49.1 (CH), 48.9 (CH), 43.4 (CH_2), 43.1 (CH_2), 41.5 (CH_2), 41.5 (CH_2), 38.8 (CH_2), 38.8 (CH_2), 37.9 (CH_2), 37.9 (CH_2), 36.3 (CH_2), 36.3 (CH_2), 36.2 (CH_2), 35.6 (CH_2), 29.3 (CH), 29.2 (CH), 28.8 (CH), 28.8 (CH), 26.9 (4 CH_2), 26.4 (CH), 26.4 (CH), 26.2 (CH), 26.2 (CH), 25.7 (4 CH), 25.5 (CH_2), 25.5 (CH_2), 25.3 (CH_2), 25.3 (CH_2), 23.5 (CH_3), 23.4 (CH_3), 23.1 (2 CH_3), 23.0 (2 CH_3), 22.0 (CH_3), 21.9 (CH_3), 21.7 (CH_3), 21.6 (CH_3), 20.3 (4 CH_3), 19.0 (2 CH_3), 18.9 (2 CH_3), 17.6 (2 CH_3).

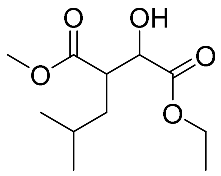

d.r. = 60:40
$C_{11}H_{20}O_5$
MW = 232,28

Compound **4cak**: Following **GP1** performed with ethyl glyoxylate (88 μL, 0.43 mmol, 1.0 equiv), methyl acrylate (39 μL, 0.43 mmol, 1.0 equiv) and organozinc reagent **1c** (3.0 mL, C = 0.43 M, 1.3 mmol, 3.0 equiv), the desired product was obtained after purification by FC [V(SiO_2) = 20 mL, Cy/AE: 80:20 (100 mL), 60:40 (100 mL)] as a colorless oil (149 mg, 69%, d.r: 60:40).

R_f (Cy/AE: 95/5, UV+*p*-Anis): 0.38.

HRMS (ESI$^+$): m/z [M+Na]$^+$ Calcd. for $C_{11}H_{20}NaO_5$: 255.1203. Found: 255.1209.

^{1}H NMR (400 MHz, $CDCl_3$): δ 4.38–4.18 (m, 5H), 3.66 (s, 3H), 3.61 (s, 3H), 3.08 (m, 1H), 2.86–2.76 (m, 1H), 2.11 (d, *J* = 2.0 Hz, 1H), 1.98 (d, *J* = 2.0 Hz, 1H), 1.85–1.56 (m, 3H), 1.63–1.40 (m, 3H), 1.29–1.10 (m, 7H), 0.87–0.80 (m, 12H).

^{13}C{^{1}H} NMR (100 MHz, $CDCl_3$): δ 173.8 (C), 173.7 (C), 173.5 (C), 173.4 (C), 71.6 (CH), 71.3 (CH), 62.3 (CH_2), 62.0 (CH_2), 52.1 (CH_3), 52.0 (CH_3), 47.5 (CH), 46.7 (CH), 36.9 (CH_2), 36.0 (CH_2), 26.2 (CH), 25.9 (CH), 23.3 (CH_3), 22.5 (CH_3), 22.4 (CH_3), 21.8 (CH_3), 14.2 (2 CH_3).

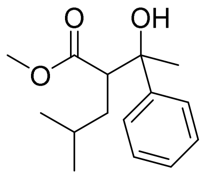

d.r. = 61:39
$C_{15}H_{22}O_3$
MW = 250,34

Compound **6caa**: Following **GP1** performed with acetophenone (50 μL, 0.43 mmol, 1.0 equiv), methyl acrylate (39 μL, 0.43 mmol, 1.0 equiv) and organozinc reagent **1c** (3.0 mL, C = 0.43 M, 1.3 mmol, 3.0 equiv), the desired product was obtained after purification by FC [V(SiO_2) = 20 mL, Cy/AE: 95:5 (100 mL), 90:10 (100 mL)] as a pale yellow oil (108 mg, 74%, d.r: 61:39).

R_f (Cy/AE: 90/10, UV+*p*-Anis): 0.60.

HRMS (ESI$^+$): m/z [M+Na]$^+$ Calcd. for $C_{15}H_{22}NaO_3$: 273.1461. Found: 273.1462.

^{1}H NMR (400 MHz, $CDCl_3$): δ 7.33 (t, *J* = 8.1 Hz, 3H), 7.31–7.20 (m, 4H), 7.20–7.07 (m, 3H), 3.87–3.55 (m, 4H), 3.27 (d, *J* = 2.0 Hz, 4H), 2.88 (d, *J* = 11.7, 1H), 2.80 (d, *J* = 11.7 Hz, 1H), 1.86–1.76 (m, 1H), 1.76–1.54 (m, 1H), 1.41 (m, 6H), 1.19 (m, 1H), 0.88–0.78 (m, 9H), 0.72–0.66 (m, 3H), 0.66–0.59 (m, 3H).

^{13}C{^{1}H} NMR (100 MHz, $CDCl_3$): δ 177.4 (C), 176.8 (C), 147.4 (C), 145.3 (C), 128.1 (2 CH), 128.0 (2 CH), 126.8 (CH), 126.6 (CH), 124.8 (2 CH), 124.7 (2 CH), 75.1 (C), 74.6 (C), 53.8 (CH_3), 53.4 (CH_3), 51.7 (CH), 51.3 (CH), 36.6 (CH_2), 36.5 (CH_2), 30.3 (CH_3), 26.9 (CH_3), 26.7 (CH), 26.3 (CH), 23.8 (CH_3), 23.5 (CH_3), 21.4 (CH_3), 21.1 (CH_3).

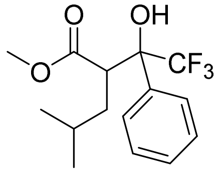

d.r. = 59:41
$C_{15}H_{19}F_3O_3$
MW = 304,31

Compound **6cab**: Following **GP1** performed with 2,2,2-trifluoroacetophenone (49 μL, 0.36 mmol, 1.0 equiv), methyl acrylate (32 μL, 0.36 mmol, 1.0 equiv) and organozinc reagent **1c** (3.0 mL, C = 0.36 M, 1.1 mmol, 3.0 equiv), the desired product was obtained after purification by FC [V(SiO_2) = 20 mL, Cy/AE: 95:5 (100 mL), 90:10 (100 mL)]as a colorless oil (110 mg, 99%, d.r: 59:41).

R*f* (Cy/AE: 90/10 , UV+*p*-Anis): 0.60.

HRMS (ESI$^+$): m/z [M+H]$^+$ Calcd. for $C_{15}H_{19}F_3O_3$: 305.1518. Found: 305.1520.

^{1}H NMR (400 MHz, CDCl$_3$): δ 7.58 (d, *J* = 7.5 Hz, 4H), 7.50–7.31 (m, 6H), 5.11 (s, 1H), 4.53 (s, 1H), 3.85 (s, 3H), 3.79–3.59 (m, 2H), 3.53 (m, 1H), 3.38 (s, 3H), 2.02 (m, 1H), 1.81–1.64 (m, 1H), 1.50–1.20 (m, 3H), 1.02 (d, *J* = 6.5 Hz, 2H), 0.99 (d, *J* = 6.6 Hz, 2H), 0.83 (d, *J* = 6.5 Hz, 4H), 0.79 (d, *J* = 6.6 Hz, 4H).

^{13}C{^{1}H} NMR (100 MHz, CDCl$_3$): δ 176.6 (C), 175.4 (C), 138.1 (C), 135.4 (C), 128.6 (2 CH), 128.6 (2 CH), 128.4 (CH), 128.2 (CH), 126.2 (2 CH), 126.1 (2 CH), 78.0 (q, *J* = 27.5 Hz, 2 C), 52.7 (CH_3), 51.9 (CH_3), 48.9 (CH), 45.5 (CH), 36.6 (CH_2), 35.8 (CH_2), 26.6 (CH), 25.8 (CH), 23.9 (CH_3), 23.5 (CH_3), 21.1 (CH_3), 20.8 (CH_3). [$\underline{C}F_3$ not detected]

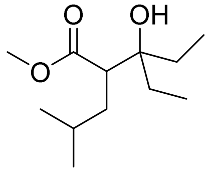

$C_{12}H_{24}O_3$
MW = 216,32

Compound **6cac**: Following **GP1** performed with 3-pentanone (30 μL, 0.32 mmol, 1.0 equiv), methyl acrylate (29 μL, 0.32 mmol, 1.0 equiv) and organozinc reagent **1c** (3.0 mL, C = 0.32 M, 0.96 mmol, 3.0 equiv), the desired product was obtained after purification by FC [V(SiO_2) = 20 mL, Cy/AE: 90:10 (100 mL), 80:20 (50 mL), 70:30 (50 mL)] as a colorless oil (69 mg, 59%).

R*f* (Cy/AE: 90/10, UV+*p*-Anis): 0.48.

HRMS (ESI$^+$): m/z [M+H]$^+$ Calcd. for $C_{12}H_{25}O_3$: 217.1013. Found: 217.1015.

^{1}H NMR (400 MHz, CDCl$_3$): δ 3.69 (s, 3H), 3.66–3.60 (m, 1H), 2.85 (s, 1H), 2.62–2.56 (m, 1H), 1.78 (t, *J* = 12.5 Hz, 1H), 1.59 (m, 1H), 1.51–1.29 (m, 3H), 1.24 (t, *J* = 11.8 Hz, 1H), 0.99–0.80 (m, 9H), 0.80–0.71 (m, 3H).

^{13}C{^{1}H} NMR (100 MHz, CDCl$_3$): δ 177.4 (C), 75.0 (C), 51.4 (CH_3), 49.5 (CH), 35.8 (CH_2), 30.2 (CH_2), 26.5 (CH), 26.4 (CH_2), 23.9 (CH_3), 21.3 (CH_3), 7.6 (2 CH_3).

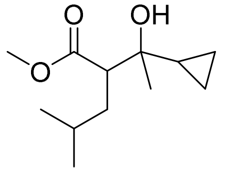

d.r. = 79:21
$C_{12}H_{22}O_3$
MW = 214,31

Compound **6cad**: Following **GP1** performed with cyclopropyl methyl ketone (43 µL, 0.43 mmol, 1.0 equiv), methyl acrylate (39 µL, 0.43 mmol, 1.0 equiv) and organozinc reagent **1c** (3.0 mL, C = 0.43 M, 1.3 mmol, 3.0 equiv), the desired product was obtained after purification by FC [V(SiO_2) = 20 mL, Cy/AE: 90:10 (100 mL), 80:20 (100 mL)] as a pale yellow oil (91 mg, 99%, d.r: 79:21).

R_f (Cy/AE: 90/10, UV+*p*-Anis): 0.60.

HRMS (ESI$^+$): m/z $[M+Na]^+$ Calcd. for $C_{12}H_{22}NaO_3$: 237.1461. Found: 237.1466.

^{1}H NMR (400 MHz, $CDCl_3$): δ 3.67 (s, 6H), 2.57–2.50 (m, 4H), 1.79–1.73 (m, 2H), 1.43–1.31 (m, 4H), 1.16 (s, 5H), 1.08 (s, 1H), 0.88–0.80 (m, 15H), 0.31–0.26 (m, 7H).

^{13}C{^{1}H} NMR (100 MHz, $CDCl_3$): δ 176.9 (C), 176.4 (C), 71.4 (C), 70.8 (C), 54.5 (CH_3), 53.7 (CH_3), 51.6 (CH), 51.5 (CH), 36.5 (CH_2), 36.3 (CH_2), 26.8 (CH), 26.6 (CH), 25.5 (CH_3), 25.0 (CH_3), 23.9 (CH_3), 23.9 (CH_3), 21.5 (CH_3), 21.5 (CH_3), 21.1 (2 CH), 1.4 (CH_2), 1.1 (CH_2), 0.2 (CH_2), –0.3 (CH_2).

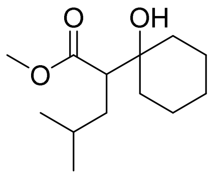

$C_{13}H_{24}O_3$
MW = 228,33

Compound **6cae**: Following **GP1** performed with cyclohexanone (35 µL, 0.34 mmol, 1.0 equiv), methyl acrylate (31 µL, 0.34 mmol, 1.0 equiv) and organozinc reagent **1c** (3.0 mL, C = 0.34 M, 1.0 mmol, 3.0 equiv), the desired product was obtained after purification by FC [V(SiO_2) = 20 mL, Cy/AE: 95:5 (100 mL), 90:10 (100 mL)] as a colorless oil (78 mg, 99%).

R_f (Cy/AE: 90/10, UV+*p*-Anis): 0.38.

HRMS (ESI$^+$): m/z $[M+Na]^+$ Calcd. for $C_{13}H_{24}NaO_3$: 251.1618. Found: 251.1621.

^{1}H NMR (400 MHz, $CDCl_3$) δ 3.67 (s, 3H), 2.68 (br s, 1H), 2.48 (d, *J* = 11.8 Hz, 1H), 1.73–1.70 (m, 2H), 1.62–1.54 (m, 3H), 1.43–1.30 (m, 6H), 1.26–1.16 (m, 2H), 0.88 (s, 6H).

^{13}C NMR (100 MHz, $CDCl_3$) δ 177.1 (C), 71.7 (C), 52.7 (CH_3), 51.4 (CH), 37.3 (CH_2), 35.6 (CH_2), 34.4 (CH_2), 26.6 (CH), 25.7 (CH_3), 23.8 (CH_3), 21.9 (CH_2), 21.7 (CH_2), 21.4 (CH_2).

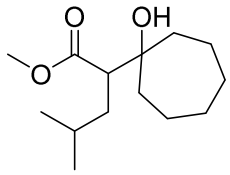

$C_{14}H_{26}O_3$
MW = 242,36

Compound **6caf**: Following **GP1** performed with cycloheptanone (38 µL, 0.34 mmol, 1.0 equiv), methyl acrylate (31 µL, 0.34 mmol, 1.0 equiv) and organozinc reagent **1c** (3.0 mL, C = 0.34 M, 1.0 mmol, 3.0 equiv), the desired product was obtained after purification by FC [V(SiO_2) = 20 mL, Cy/AE: 95:5 (100 mL), 90:10 (100 mL), 70:30 (50 mL)] as a pale yellow oil (40 mg, 48%).

R_f (Cy/AE: 90/10, UV+*p*-Anis): 0.25.

HRMS (ESI$^+$): m/z $[M+Na]^+$ Calcd. for $C_{14}H_{26}NaO_3$: 265.1774. Found: 265.1780

^{1}H NMR (400 MHz, $CDCl_3$): δ 3.62 (s, 3H), 2.73 (s, 1H), 2.47 (d, *J* = 11.7 Hz, 1H), 1.77–1.42 (m, 10H), 1.37–1.24 (m, 5H), 0.83 (s, 6H).

^{13}C{^{1}H} NMR (100 MHz, $CDCl_3$): δ 177.0 (C), 75.7 (C), 52.9 (CH_3), 51.3 (CH), 40.9 (CH_2), 37.4 (CH_2), 36.1 (CH_2), 29.7 (CH_2), 29.5 (CH_2), 26.6 (CH), 23.8 (CH_3), 22.2 (CH_2), 21.9 (CH_2), 21.4 (CH_3).

3.3. CR with sulfonyl imines

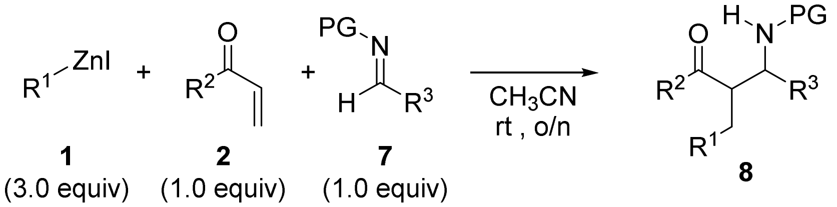

General procedure 2 (GP2): A 10 mL sealable tube equipped with a stir bar and filled with an argon atmosphere was charged with imine **7** (0.4 mmol, 1.0 equiv), acrylate **2** (2.0 equiv) and acetonitrile (2 mL). Under stirring, a solution of organozinc reagent **1** in CH_3CN (3.0 equiv) was added in one portion. The reaction was stirred at room temperature overnight. Then, the reaction mixture was poured into sat aq NH_4Cl (20 mL). The resulting solution was extracted twice with EtOAc (15+15 mL) and the combined organic layers were washed brine, dried over anhydrous Na_2SO_4 and evaporated. The resulting crude material was purified on silica gel to give product **7**.

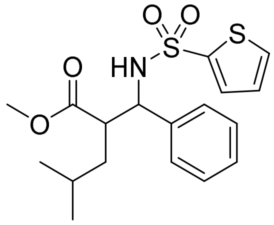

d.r. = 68:32
$C_{18}H_{23}NO_4S_2$
MW = 381,51

Compound **8caa**: Following **GP2** performed with imine **7a** (251 mg, 1.0 mmol, 1.0 equiv), methyl acrylate (181 µL, 2.0 mmol, 2.0 equiv) and organozinc reagent (3.2 mL, C = 0.93 M, 3.0 mmol, 3.0 equiv), the desired product was obtained after purification by FC [V(SiO_2) = 50 mL, Cy/AE: 90:10 (250 mL), 80:20 (250 mL)] as a white solid (242 mg, 63%, d.r. = 68:32).

Melting Point (°C): 97.0

R*f* (Cy/AE: 80/20, UV+$KMnO_4$): 0.32, 0.22.

HRMS (ESI⁺): m/z [M+H]⁺ Calcd. for $C_{18}H_{24}NO_4S_2$: 382.1147. Found: 382.1145.

^{1}H NMR (400 MHz, $CDCl_3$) δ 7.38 (d, *J* = 4.6 Hz, 1H), 7.35–7.31 (m, 2H), 7.26–7.24 (m, 2H), 7.14 (br s, 5H), 7.02–7.01 (m, 4H), 6.84–6.82 (m, 1H), 6.79–6.77 (m, 1H), 6.35 (d, *J* = 9.3 Hz, 1H), 5.86 (d, *J* = 8.9 Hz, 1H), 4.62–4.60 (m, 1H), 4.54–4.50 (m, 1H), 3.53 (s, 3H), 3.39 (s, 3H), 2.83–2.78 (m, 2H), 1.66–1.62 (m, 2H), 1.55–1.44 (m, 3H), 1.30 –1.25 (m, 1H), 0.84 (d, *J* = 6.4 Hz, 12H).

^{13}C NMR (100 MHz, $CDCl_3$) δ 175.1 (C), 173.4 (C), 142.1 (C), 141.4 (C), 139.0 (C), 138.2 (C), 132.4 (CH), 132.0 (CH), 131.8 (CH), 131.5 (CH), 128.5 (2 CH), 128.4 (2 CH), 127.9 (CH), 127.7 (CH), 127.2 (CH), 127.0 (CH), 126.8 (2 CH), 126.2 (2 CH), 60.1 (CH), 59.3 (CH), 51.9 (CH_3), 51.7 (CH_3), 50.6 (CH), 50.1 (CH), 39.2 (CH_2), 37.7 (CH_2), 26.2 (CH), 25.9 (CH), 23.4 (CH_3), 22.8 (CH_3), 22.0 (CH_3), 21.6 (CH_3).

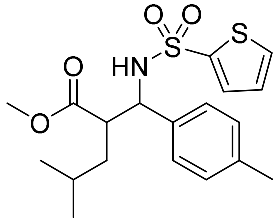

Compound **8cab**: Following **GP2** performed with imine **7b** (265 mg, 1.0 mmol, 1.0 equiv), methyl acrylate (181 µL, 2.0 mmol, 2.0 equiv) and organozinc reagent (3.2 mL, C = 0.93 M, 3.0 mmol, 3.0 equiv), the desired product was obtained after purification by FC [V(SiO_2) = 50 mL, Cy/AE: 90:10 (250 mL), 80:20 (250 mL)] as a white solid (346 mg, 87%, d.r. = 58:42).

Melting Point (°C): 96.5

R*f* (Cy/AE: 80/20, UV+KMnO4): 0.35, 0.27.

HRMS (ESI+): m/z [M+H]+ Calcd. for $C_{19}H_{26}NO_4S_2$: 396.1297. Found: 396.1298.

^{1}H NMR (400 MHz, CDCl3) δ 7.33 (d, *J* = 4.7 Hz, 1H), 7.29 (d, *J* = 4.7 Hz, 1H), 7.26–7.25 (m, 1H), 7.20–7.19 (m, 1H), 6.90–6.84 (m, 8H), 6.78–6.77 (m, 1H), 6.74–6.72 (m, 1H), 6.24 (d, *J* = 9.0 Hz, 1H), 5.80 (d, *J* = 8.9 Hz, 1H), 4.59 (t, *J* = 6.9 Hz, 1H), 4.42 (t, *J* = 8.3 Hz, 1H), 3.48 (s, 3H), 3.34 (s, 3H), 2.75–2.70 (m, 2H), 2.18 (s, 6H), 1.58–1.51 (m, 2H), 1.45–1.38 (m, 3H), 1.20–1.15 (m, 1H), 0.76 (d, *J* = 6.3 Hz, 12H).

^{13}C NMR (100 MHz, CDCl3) δ 174.1 (C), 173.5 (C), 142.1 (C), 141.5 (C), 137.5 (C), 137.3 (C), 136.1 (C), 135.2 (C), 132.4 (CH), 132.0 (CH), 121.7 (CH), 131.4 (CH), 129.2 (2 CH), 129.0 (2 CH), 127.1 (CH), 127.0 (CH), 126.7 (2 CH), 126.1 (2 CH), 59.8 (CH), 59.1 (CH), 51.9 (CH_3), 51.7 (CH_3), 50.5 (CH), 50.2 (CH), 39.1 (CH_2), 37.8 (CH_2), 26.2 (CH), 25.9 (CH), 23.4 (CH_3), 22.8 (CH_3), 22.0 (CH_3), 21.6 (CH_3), 21.1 (CH_3), 21.1 (CH_3).

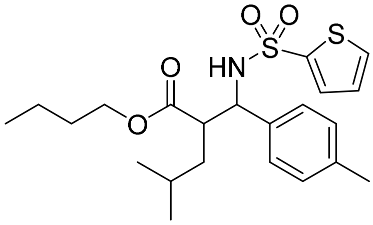

Compound **8ccb**: Following **GP2** performed with imine **7b** (265 mg, 1.0 mmol, 1.0 equiv), *n*-butyl acrylate (285 µL, 2.0 mmol, 2.0 equiv) and organozinc reagent (3.2 mL, C = 0.93 M, 3.0 mmol, 3.0 equiv), the desired product was obtained after purification by FC [V(SiO_2) = 50 mL, Cy/AE: 90:10 (250 mL), 80:20 (250 mL)] as a white solid (414 mg, 95%, d.r. = 53:47).

Melting Point (°C): 89.5

R*f* (Cy/AE: 80/20, UV+KMnO4): 0.48.

HRMS (ESI+): m/z [M+H]+ Calcd. for $C_{22}H_{32}NO_4S_2$: 438.1773. Found: 438.1776.

^{1}H NMR (400 MHz, CDCl3) δ 7.33 (d, *J* = 4.6 Hz, 1H), 7.30 (d, *J* = 4.7 Hz, 1H), 7.24–7.20 (m, 3H), 6.88–6.84 (m, 7H), 6.78–6.74 (m, 2H), 6.27 (d, *J* = 9.1 Hz, 1H), 5.72 (d, *J* = 8.9 Hz, 1H), 4.52–4.49 (m, 1H), 4.38 (t, *J* = 8.4 Hz, 1H), 3.87 (q, *J* = 5.5 Hz, 2H), 3.72 (q, *J* = 5.9 Hz, 2H), 2.72–2.68 (m, 2H), 2.17 (s, 6H), 1.59–1.52 (m, 2H), 1.56–1.33 (m, 4H), 1.27–1.15 (m, 3H), 1.10–1.03 (m, 3H), 0.88–0.83 (m, 2H), 0.78–0.73 (m, 18H).

^{13}C NMR (100 MHz, CDCl$_3$) δ 174.9 (C), 173.2 (C), 142.3 (C), 141.6 (C), 137.5 (C), 137.2 (C), 136.1 (C), 135.4 (C), 132.4 (CH), 132.0 (CH), 131.7 (CH), 131.3 (CH), 129.1 (2 CH), 129.0 (2 CH), 127.1 (CH), 126.9 (CH), 126.8 (2 CH), 126.1 (2 CH), 64.7 (CH), 64.5 (CH), 60.0 (CH$_2$), 59.1 (CH$_2$), 50.7 (CH), 50.1 (CH), 39.3 (CH$_2$), 38.1 (CH$_2$), 30.5 (CH$_2$), 30.4 (CH$_2$), 26.3 (CH), 25.9 (CH), 23.5 (CH$_2$), 22.8 (CH$_2$), 22.0 (CH$_2$), 21.5 (CH$_2$), 21.1 (CH$_3$), 21.0 (CH$_3$), 19.0 (CH$_3$), 18.9 (CH$_3$), 13.7 (2 CH$_3$).

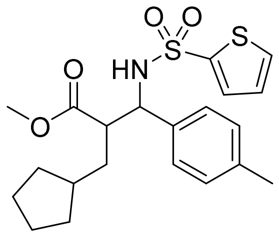

d.r. = 52:48
$C_{21}H_{27}NO_4S_2$
MW = 421,57

Compound **8dab**: Following **GP2** performed with imine **7b** (265 mg, 1.0 mmol, 1.0 equiv), methyl acrylate (181 µL, 2.0 mmol, 2.0 equiv) and organozinc reagent (3.2 mL, C = 0.95 M, 3.0 mmol, 3.0 equiv), the desired product was obtained after purification by FC [V(SiO$_2$) = 50 mL, Cy/AE: 90:10 (250 mL), 80:20 (250 mL)] as a colorless oil (153 mg, 36%, d.r. = 52:48).

R_f (Cy/AE: 80/20, UV+KMnO$_4$): 0.30.

HRMS (ESI$^+$): m/z [M+H]$^+$ Calcd. for $C_{21}H_{28}NO_4S_2$: 422.1460. Found: 422.1462.

^{1}H NMR (400 MHz, CDCl$_3$) δ 7.35 –7.29 (m, 2H), 7.26–7.19 (m, 2H), 6.88–6.82 (m, 8H), 6.80–6.73 (m, 2H), 6.21 (d, *J* = 9.2 Hz, 1H), 5.66 (d, *J* = 8.6 Hz, 1H), 4.52 (t, *J* = 7.3 Hz, 1H), 4.43 (t, *J* = 8.4 Hz, 1H), 3.48 (s, 3H), 3.36 (s, 3H), 2.68 (t, *J* = 6.7 Hz, 2H), 2.18 (s, 6H), 1.76–1.31 (m, 20H), 1.06 –0.91 (m, 2H).

^{13}C NMR (100 MHz, CDCl$_3$) δ 175.2 (C), 173.6 (C), 142.2 (C), 141.5 (C), 137.5 (C), 137.3 (C), 136.2 (C), 135.3 (C), 132.3 (CH), 132.0 (CH), 131.8 (CH), 131.4 (CH), 129.2 (2 CH), 129.1 (2 CH), 127.1 (CH), 127.0 (CH), 126.7 (2 CH), 126.1 (2 CH), 59.7 (CH), 59.1 (CH), 51.9 (CH$_3$), 51.8 (CH$_3$), 51.7 (CH), 51.5 (CH), 38.1 (CH$_2$), 37.8 (CH$_2$), 36.6 (CH$_2$), 35.0 (CH$_2$), 33.1 (CH$_2$), 32.6 (CH$_2$), 32.5 (CH$_2$), 32.1 (CH$_2$), 25.1 (CH$_2$), 25.0 (CH$_2$), 21.2 (CH), 21.1 (CH).

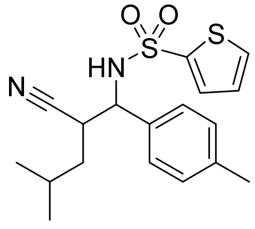

d.r. = 53:47
$C_{18}H_{22}N_2O_2S_2$
MW = 362,51

Compound **8cib**: Following **GP2** performed with imine **7b** (265 mg, 1.0 mmol, 1.0 equiv), acrylonitrile (131 µL, 2.0 mmol, 2.0 equiv) and organozinc reagent (3.7 mL, C = 0.80 M, 3.0 mmol, 3.0 equiv), the desired product was obtained after purification by FC [V(SiO$_2$) = 50 mL, Cy/AE: 90:10 (250 mL), 80:20 (250 mL)] as a colorless oil (269 mg, 74%, d.r. = 53:47).

R_f (Cy/AE: 80/20, UV+KMnO$_4$): 0.20.

HRMS (ESI$^+$): m/z [M+H]$^+$ Calcd. for $C_{18}H_{23}N_2O_2S_2$: 363.1201. Found: 363.1203.

^{1}H NMR (400 MHz, CDCl$_3$) δ 7.45 (d, *J* = 4.8 Hz, 1H), 7.41–7.36 (m, 3H), 7.04–7.01 (m, 8H), 6.90–6.82 (m, 2H), 6.14 (br s, 1H), 6.03 (br s, 1H), 4.55 (br s, 1H), 4.40 (br s, 1H), 3.19 (q, *J* = 6.6 Hz, 1H), 2.91 (dt, *J* = 9.1, 4.1 Hz, 1H), 2.27 (s, 3H), 2.26 (s, 3H), 1.85–1.68 (m, 3H), 1.42–1.28 (m, 3H), 0.93 –0.86 (m, 12H).

^{13}C NMR (100 MHz, $CDCl_3$) δ 141.0 (C), 140.9 (C), 138.7 (C), 138.3 (C), 134.1 (C), 132.8 (C), 132.6 (CH), 132.5 (CH), 132.2 (CH), 132.05 (CH), 129.5 (2 CH), 129.4 (2 CH), 127.3 (CH), 127.2 (CH), 127.0 (2 CH), 126.2 (2 CH), 119.7 (C), 119.5 (C), 58.8 (CH), 58.7 (CH), 38.6 (CH), 38.2 (CH_2), 38.1 (CH_2), 37.7 (CH), 26.0 (CH_3), 25.9 (CH_3), 22.9 (CH), 22.8 (CH), 21.4 (CH_3), 21.3 (CH_3), 21.2 (CH_3), 21.1 (CH_3).

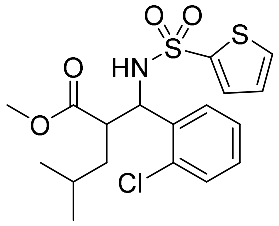

d.r. = 69:31
$C_{18}H_{22}ClNO_4S_2$
MW = 415,95

Compound **8cac**: Following **GP2** performed with imine **7c** (286 mg, 1.0 mmol, 1.0 equiv), methyl acrylate (181 µL, 2.0 mmol, 2.0 equiv) and organozinc reagent (3.8 mL, C = 0.79 M, 3.0 mmol, 3.0 equiv), the desired product was obtained after purification by FC [V(SiO_2) = 50 mL, Cy/AE: 90:10 (250 mL), 80:20 (250 mL)] as a colorless oil (297 mg, 72%, d.r. = 69:31).

R*f* (Cy/AE: 80/20, UV+$KMnO_4$): 0.23.

HRMS (ESI$^+$): m/z [M+H]$^+$ Calcd. for $C_{18}H_{23}ClNO_4S_2$: 416.0752. Found: 416.0752.

^{1}H NMR (400 MHz, $CDCl_3$) δ 7.33–7.28 (m, 4H), 7.21–7.11 (m, 3H), 7.04–7.01 (m, 4H), 6.97–6.94 (m, 1H), 6.77–6.73 (m, 2H), 6.53 (d, *J* = 9.3 Hz, 1H), 6.00 (d, *J* = 8.6 Hz, 1H), 4.98 (dd, *J* = 9.0, 4.5 Hz, 2H), 3.43 (s, 3H), 3.33 (s, 3H), 2.90–2.85 (m, 2H), 1.64–1.47 (m, 4H), 1.37–1.28 (m, 2H), 0.82–0.75 (m, 12H).

^{13}C NMR (100 MHz, $CDCl_3$) δ 174.9 (C), 173.3 (C), 141.4 (C), 140.7 (C), 136.3 (2 C), 132.5 (C), 132.2 (C), 132.1 (2 CH), 131.7 (2 CH), 129.9 (CH), 129.6 (CH), 129.1 (CH), 128.9 (CH), 127.5 (2 CH), 127.1 (2 CH), 126.9 (CH), 126.8 (CH), 55.6 (2 CH), 51.9 (CH_3), 51.8 (CH_3), 48.2 (CH), 47.3 (CH), 39.2 (2 CH_2), 26.3 (CH), 25.7 (CH), 23.4 (CH_3), 22.7 (CH_3), 22.2 (CH_3), 21.3 (CH_3).

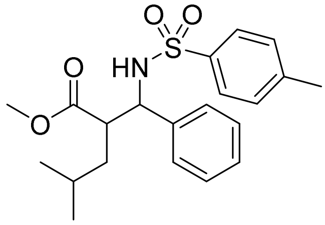

d.r. = 56:44
$C_{21}H_{27}NO_4S$
MW = 389,51

Compound **8cad**: Following **GP2** performed with imine **7d** (259 mg, 1.0 mmol, 1.0 equiv), methyl acrylate (181 µL, 2.0 mmol, 2.0 equiv) and organozinc reagent (3.7 mL, C = 0.80 M, 3.0 mmol, 3.0 equiv), the desired product was obtained after purification by FC [V(SiO_2) = 50 mL, Cy/AE: 90:10 (250 mL), 80:20 (250 mL)] as a colorless oil (289 mg, 74%, d.r. = 56:44).

R*f* (Cy/AE: 80/20, UV+$KMnO_4$): 0.23.

HRMS (ESI$^+$): m/z [M+H]$^+$ Calcd. for $C_{21}H_{28}NO_4S$: 390.1013. Found: 390.1010.

^{1}H NMR (400 MHz, $CDCl_3$) δ 7.52–7.46 (m, 4H), 7.09–6.96 (m, 14H), 6.25 (d, *J* = 9.3 Hz, 1H), 6.01 (d, *J* = 9.1 Hz, 1H), 4.52 (t, *J* = 7.7 Hz, 1H), 4.41 (t, *J* = 8.6 Hz, 1H), 3.51 (s, 3H), 3.33 (s, 3H), 2.80–2.73 (m, 2H), 2.29 (s, 3H), 2.28 (s, 3H), 1.61–1.53 (m, 2H), 1.48–1.41 (m, 4H), 0.80–0.74 (m, 12H).

^{13}C NMR (100 MHz, CDCl$_3$) δ 174.9 (C), 173.5 (C), 143.0 (C), 142.8 (C), 139.2 (C), 138.5 (C), 138.0 (C), 137.4 (C), 129.3 (2 CH), 129.1 (2 CH), 128.4 (2 CH), 128.2 (2 CH), 127.5 (CH), 127.4 (CH), 127.2 (2 CH), 126.9 (4 CH), 126.4 (2 CH), 59.9 (CH), 59.2 (CH), 51.8 (CH$_3$), 51.5 (CH$_3$), 50.7 (CH), 50.2 (CH), 39.1 (CH$_2$), 37.8 (CH$_2$), 26.2 (CH$_3$), 25.9 (CH$_3$), 23.3 (CH), 22.8 (CH), 21.8 (2 CH$_3$), 21.4 (2 CH$_3$).–

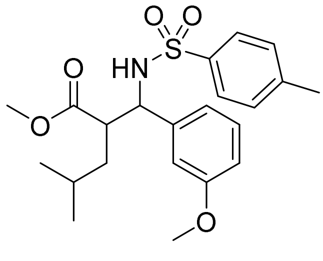

d.r. = 55:45
$C_{22}H_{29}NO_5S$
MW = 419,54

Compound **8cae**: Following **GP2** performed with imine **7e** (289 mg, 1.0 mmol, 1.0 equiv), methyl acrylate (181 µL, 2.0 mmol, 2.0 equiv) and organozinc reagent (3.8 mL, C = 0.79 M, 3.0 mmol, 3.0 equiv), the desired product was obtained after purification by FC [V(SiO$_2$) = 50 mL, Cy/AE : 90:10 (250 mL), 80:20 (250 mL)] as a white solid (318 mg, 76%, d.r. = 55:45).

Melting Point (°C): 91

R*f* (Cy/AE: 80/20, UV+KMnO$_4$): 0.17, 0.10.

HRMS (ESI$^+$): m/z [M+H]$^+$ Calcd. for $C_{22}H_{30}NO_5S$: 420.1839. Found: 420.1838.

^{1}H NMR (400 MHz, CDCl$_3$) δ 7.51–7.46 (m, 4H), 7.07–6.98 (m, 6H), 6.64– 6.57 (m, 4H), 6.43–6.40 (m, 2H), 6.19 (d, *J* = 9.3 Hz, 1H), 5.86 (d, *J* = 9.0 Hz, 1H), 4.48 (t, *J* = 7.5 Hz, 1H), 4.38 (t, *J* = 8.5 Hz, 1H), 3.64 (s, 6H), 3.53 (s, 3H), 3.38 (s, 3H), 2.79 –2.73 (m, 2H), 2.30 (s, 3H), 2.29 (s, 3H), 1.61–1.54 (m, 2H), 1.48–1.42 (m, 4H), 0.81–0.76 (m, 12H).

^{13}C NMR (100 MHz, CDCl$_3$) δ 175.0 (C), 173.5 (C), 159.6 (C), 159.3 (C), 143.1 (C), 142.8 (C), 140.8 (C), 140.0 (C), 138.1 (C), 137.5 (C), 129.5 (CH), 129.3 (CH), 129.3 (2 CH), 129.2 (2 CH), 127.2 (2 CH), 127.0 (2 CH), 199.3 (CH), 118.9 (CH), 113.3 (CH), 113.1 (CH)112.4 (CH), 111.9 (CH), 59.8 (CH), 59.2 (CH), 55.1 (2 CH$_3$), 51.9 (CH$_3$), 51.7 (CH$_3$), 50.5 (CH), 50.1 (CH), 39.2 (CH$_2$), 37.8 (CH$_2$), 26.2 (CH$_3$), 25.9 (CH$_3$), 23.4 (CH), 22.8 (CH), 21.9 (CH$_3$), 21.5 (CH$_3$), 21.5 (CH$_3$), 21.4 (CH$_3$).

4. Copies of NMR spectra

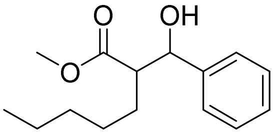

4aaa
d.r. = 74:26

[^{1}H NMR, 400 MHz, $CDCl_3$]

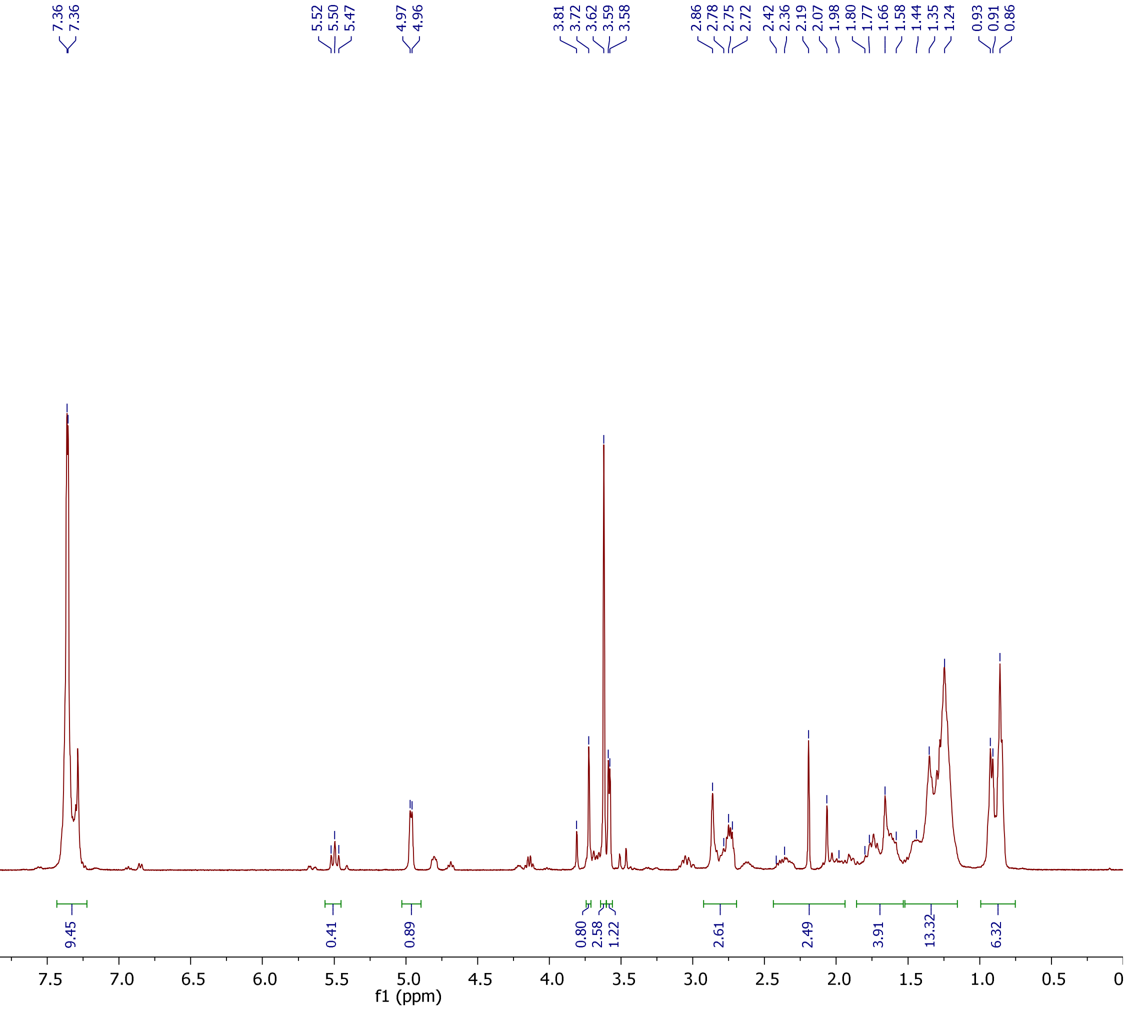

[$^{13}C\{^{1}H\}$ NMR, 100 MHz, $CDCl_3$]

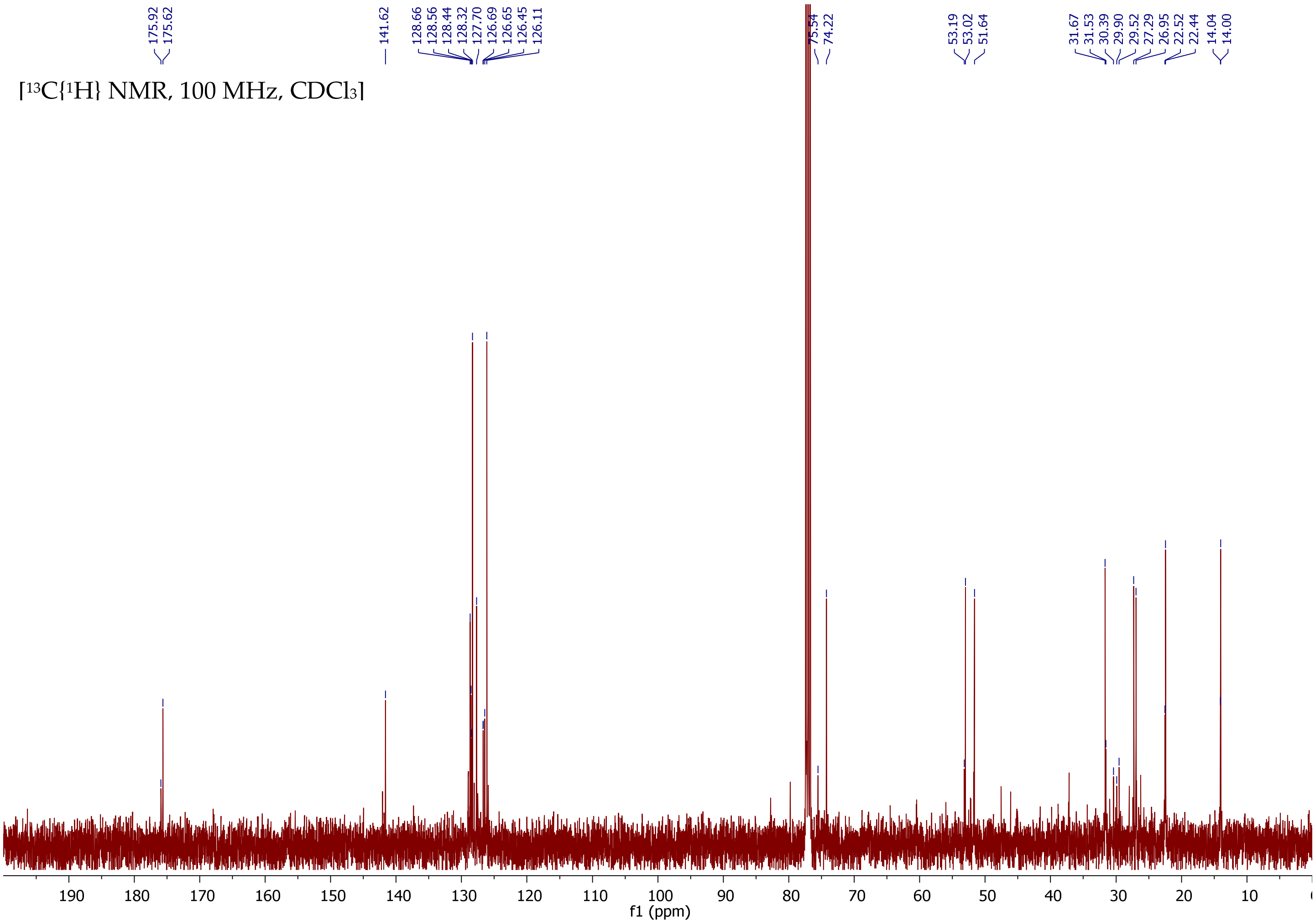

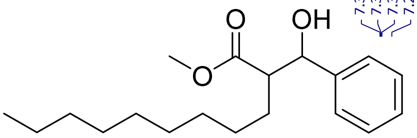

4baa
d.r. = 60:40

[^{1}H NMR, 400 MHz, $CDCl_3$]

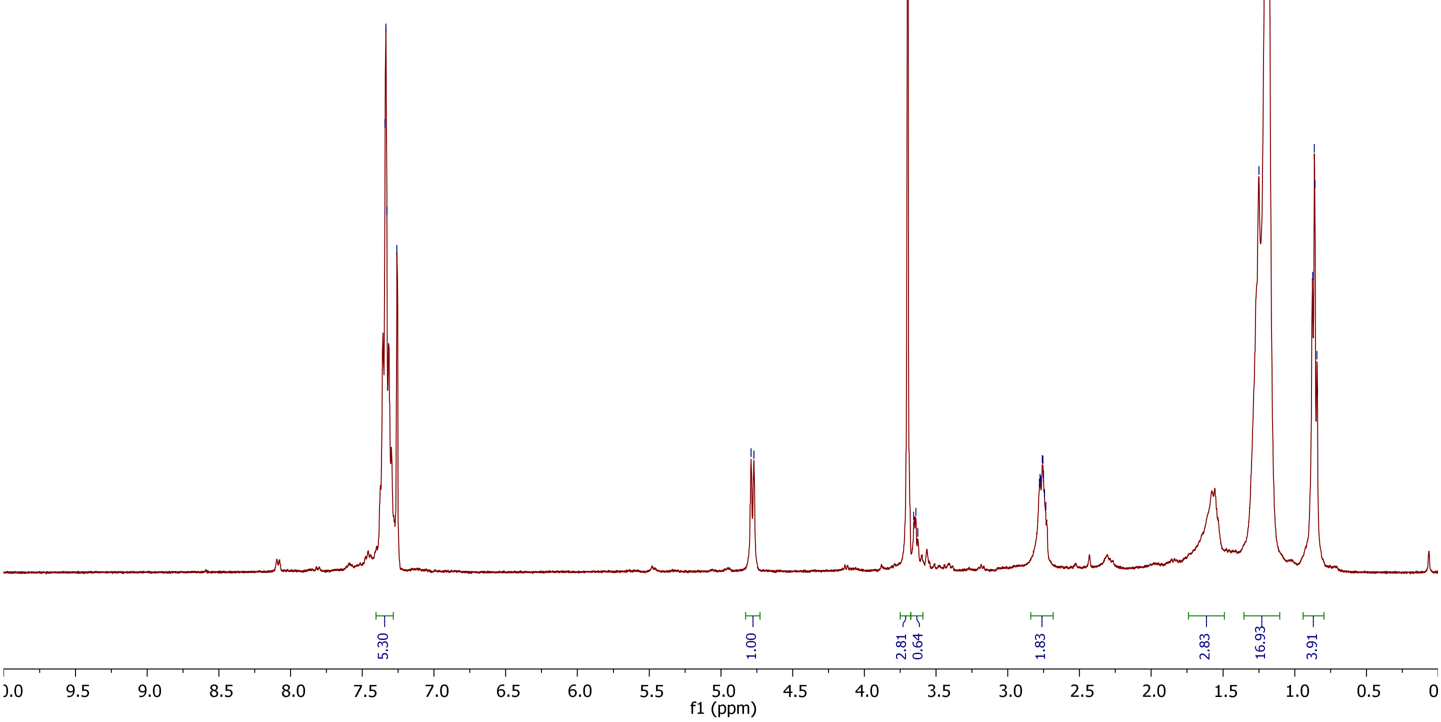

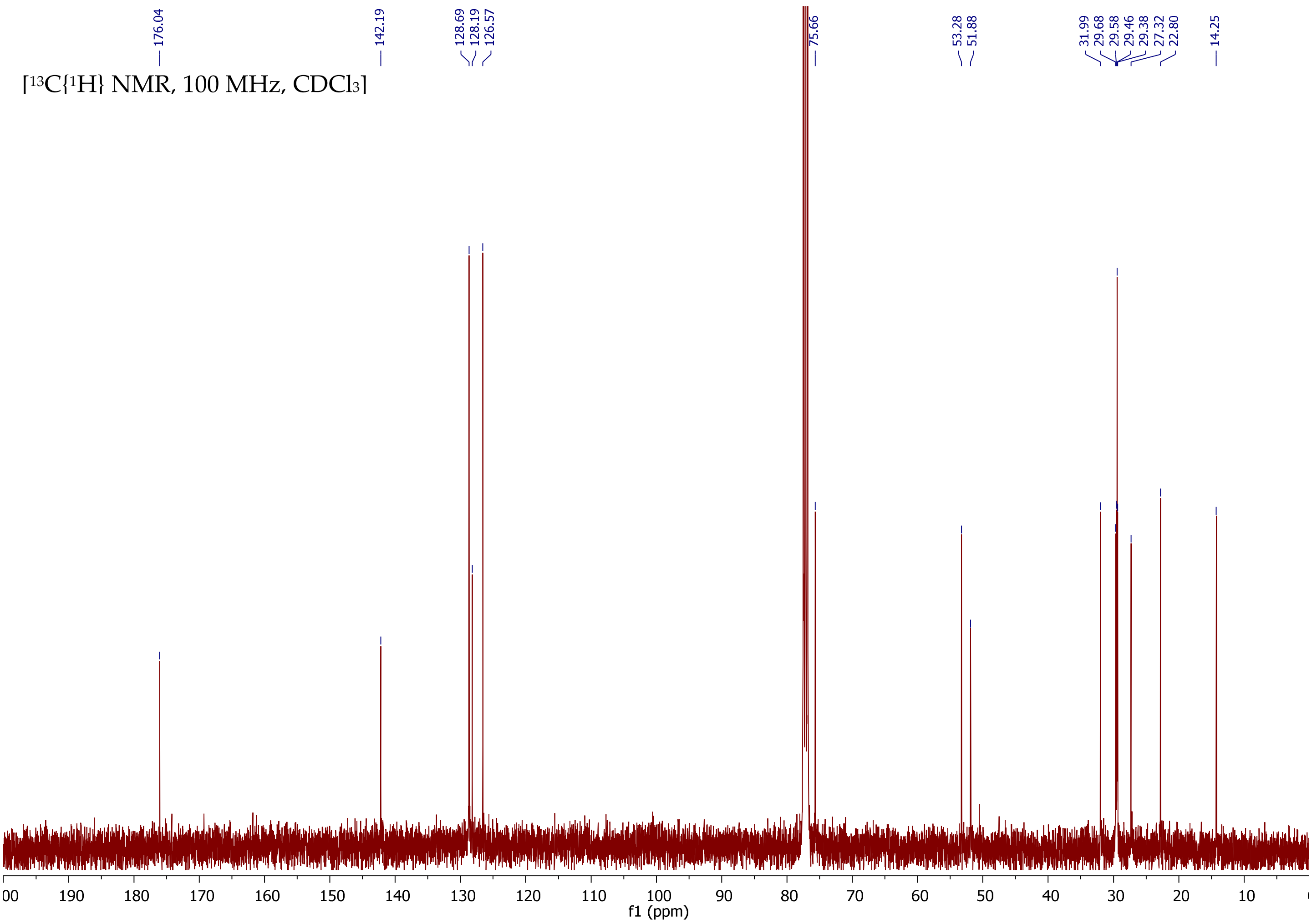
[13C{1H} NMR, 100 MHz, CDCl3]
176.04
142.19
128.69
128.19
126.57
75.66
53.28
51.88
31.99
29.68
29.58
29.46
29.38
27.32
22.80
14.25
f1 (ppm)

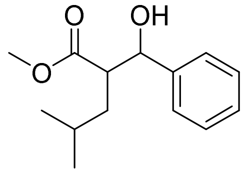

4caa
d.r. = 90:10

[^{1}H NMR, 400 MHz, $CDCl_3$]

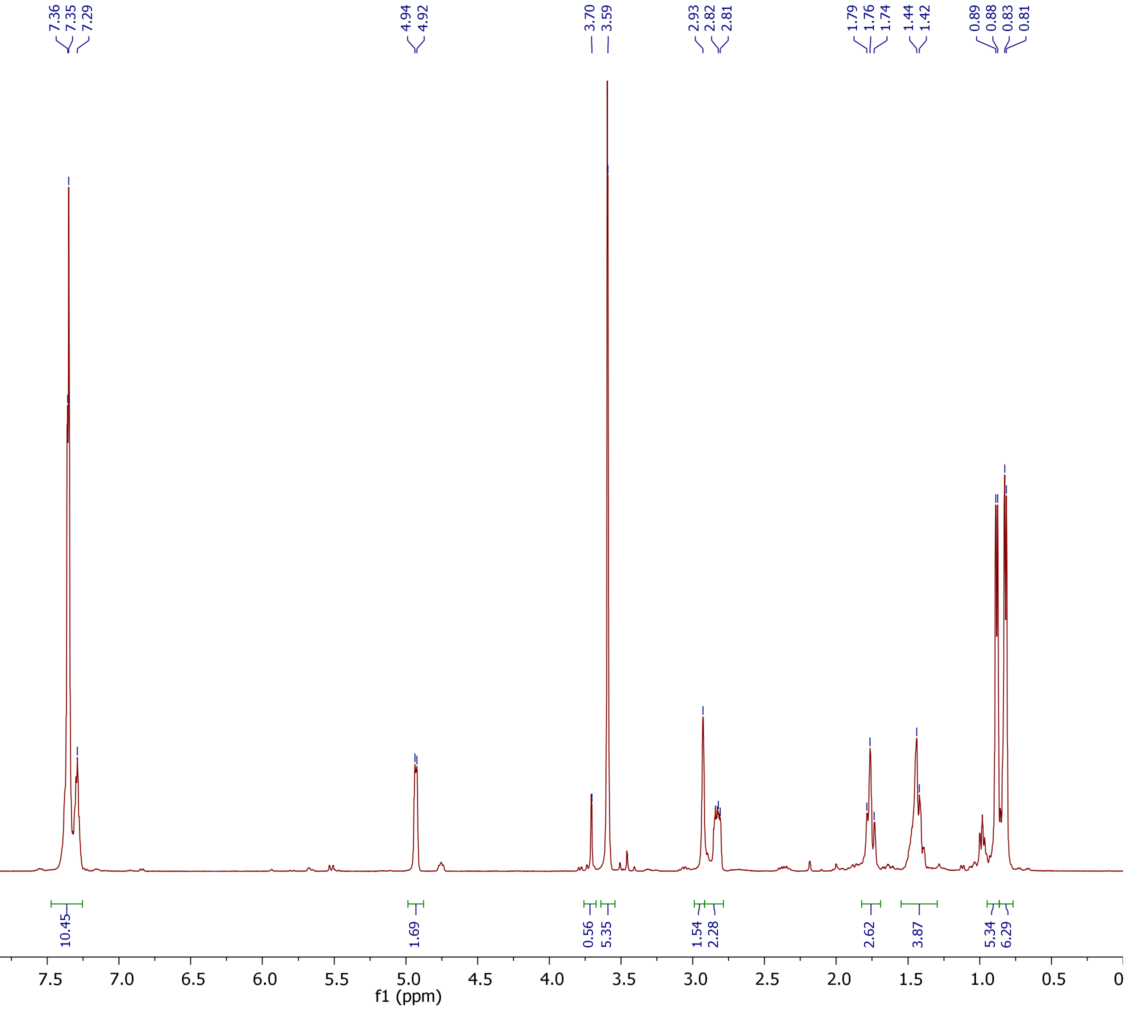

[$^{13}C\{^{1}H\}$ NMR, 100 MHz, $CDCl_3$]

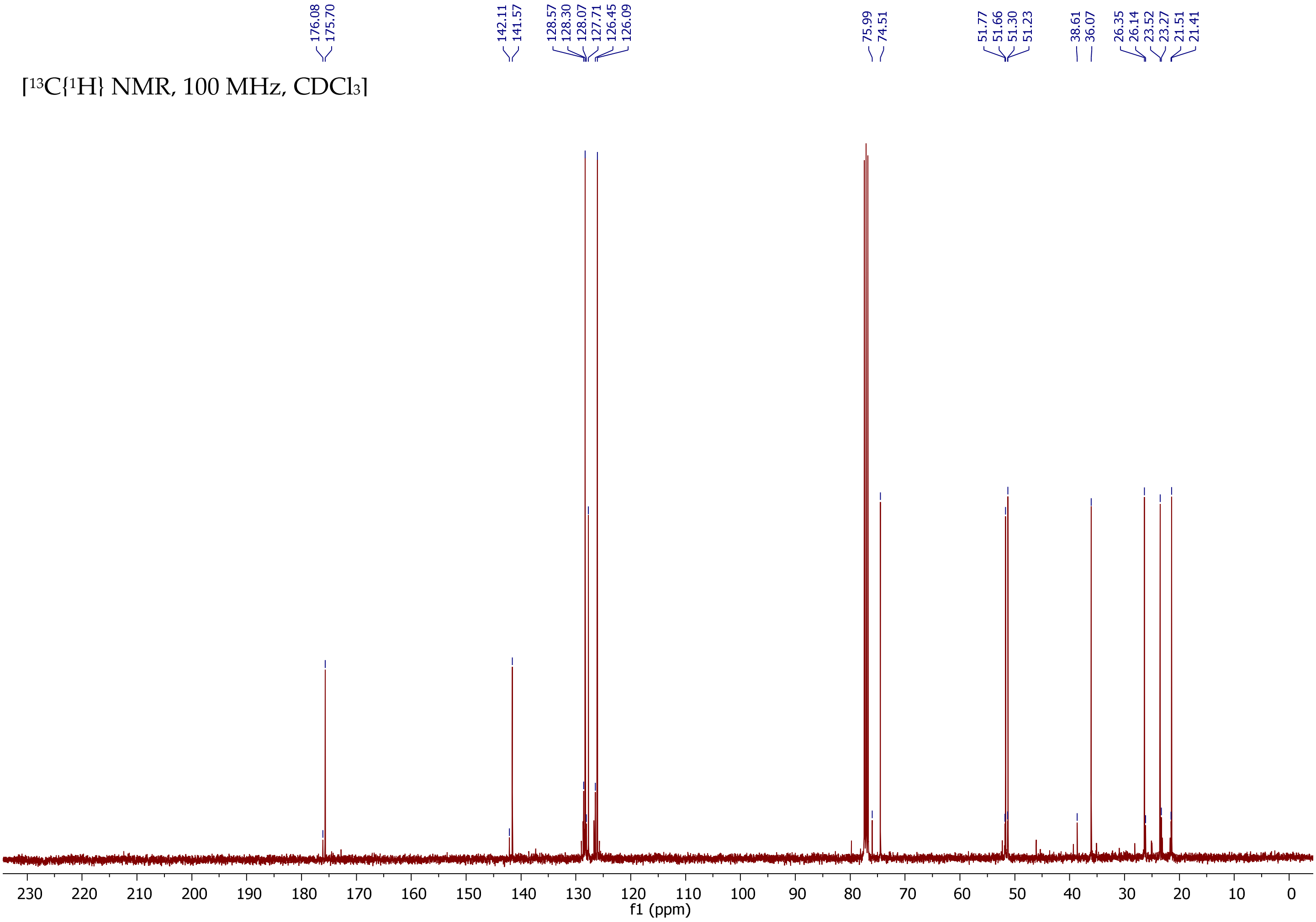

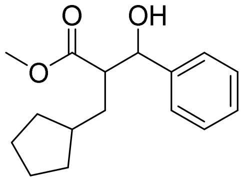

4daa
d.r. = 86:14

[^{1}H NMR, 400 MHz, $CDCl_3$]

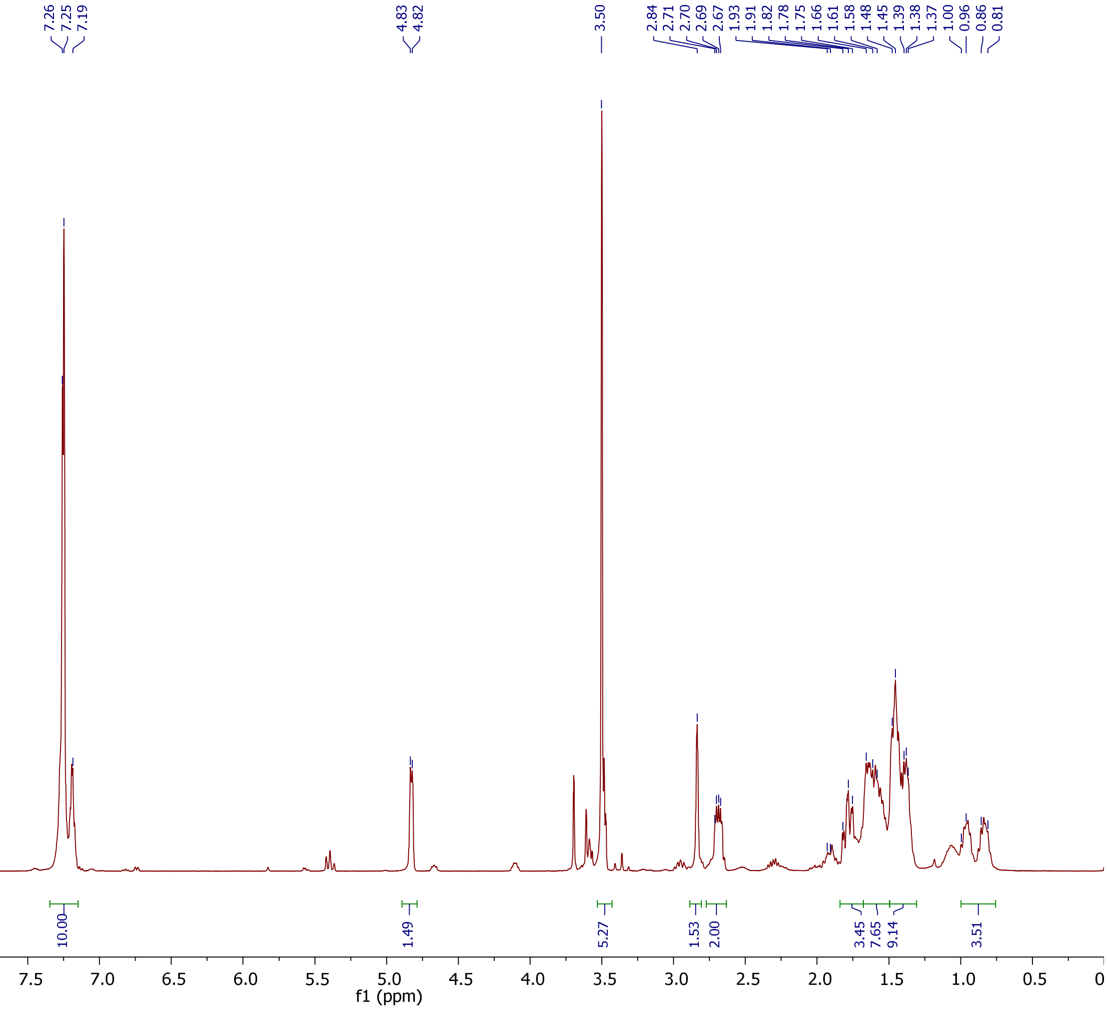

[$^{13}C\{^{1}H\}$ NMR, 100 MHz, $CDCl_3$]

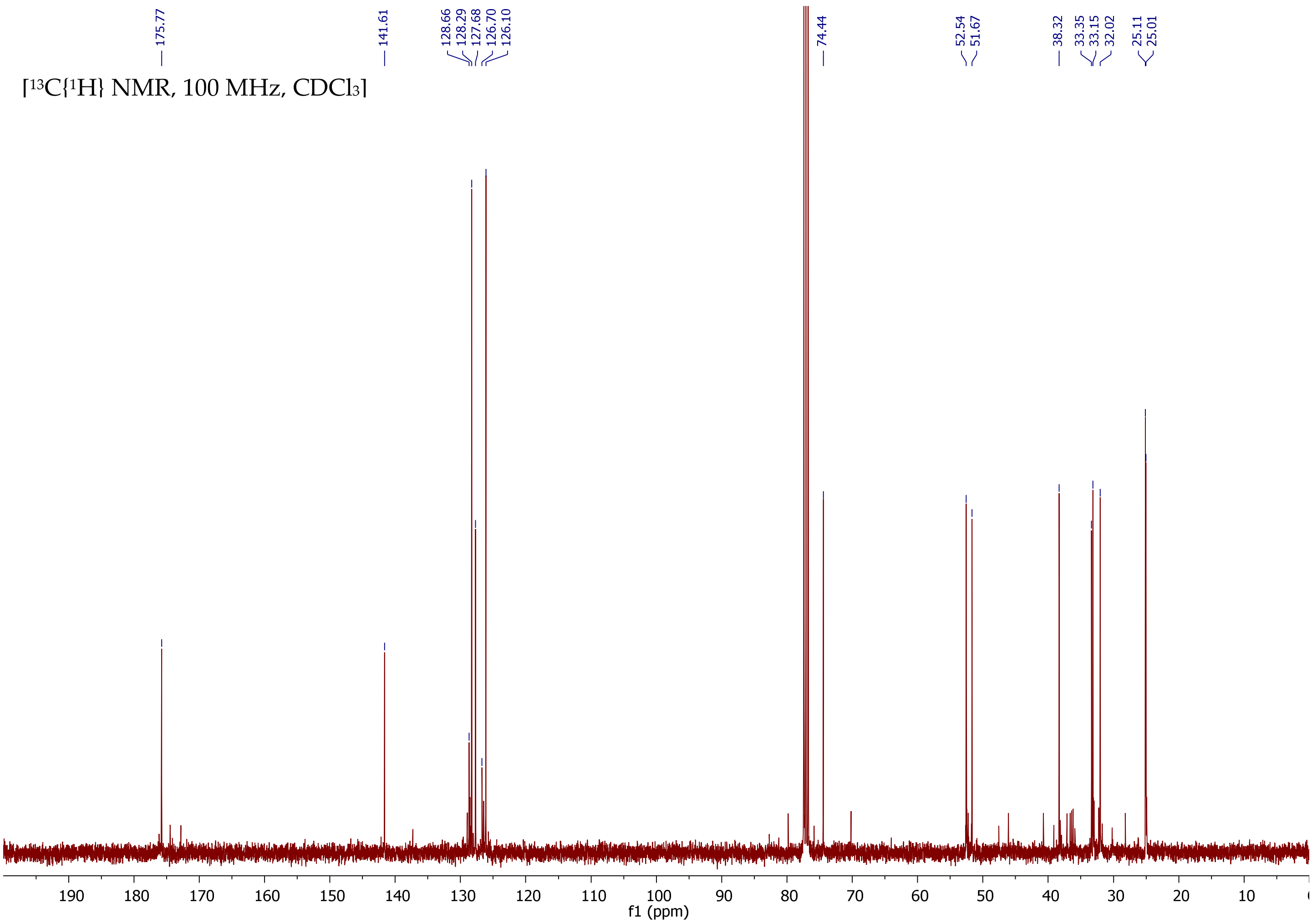

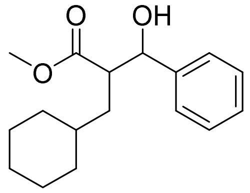

4eaa
d.r. = 88:12

[^{1}H NMR, 400 MHz, $CDCl_3$]

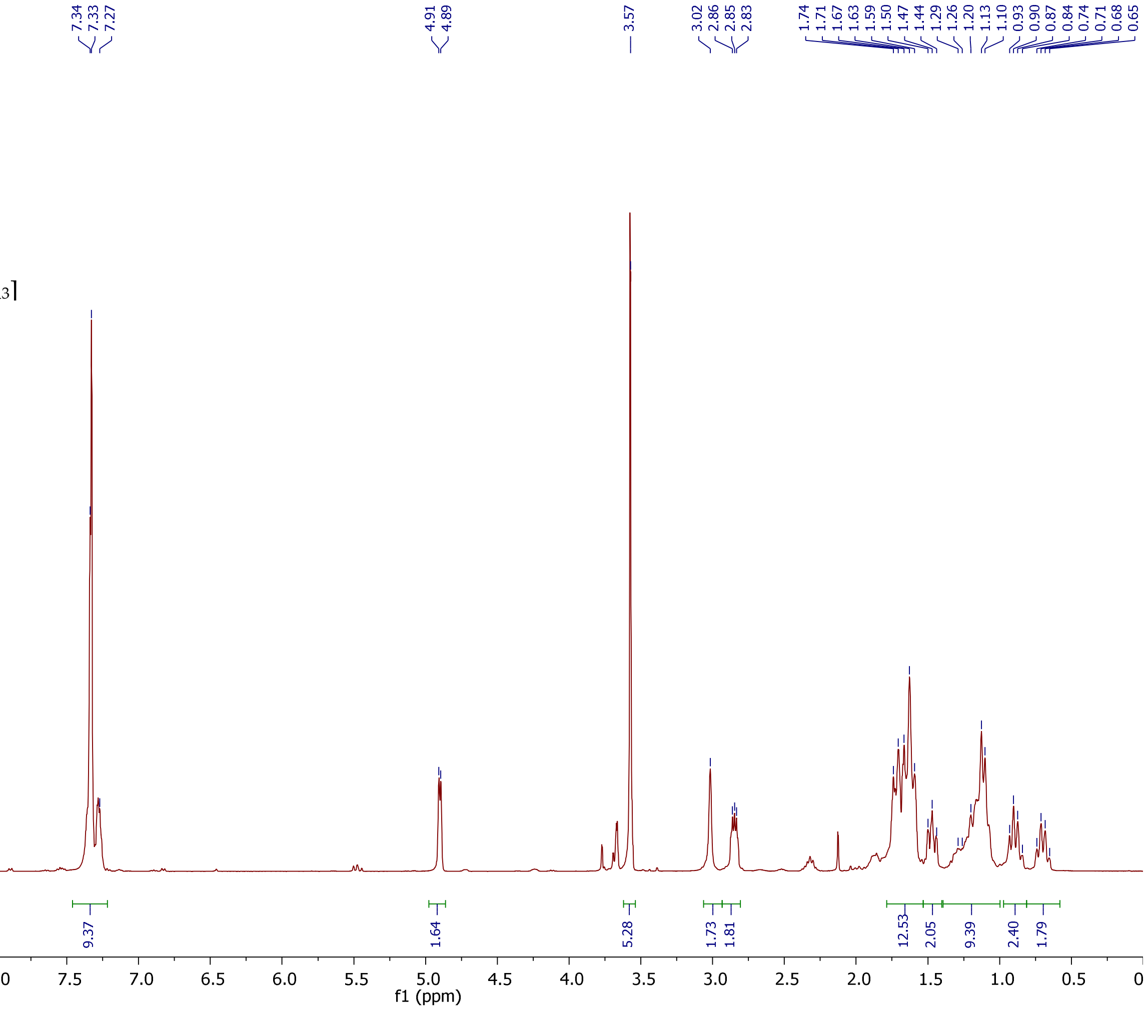

[13C{1H} NMR, 100 MHz, CDCl3]

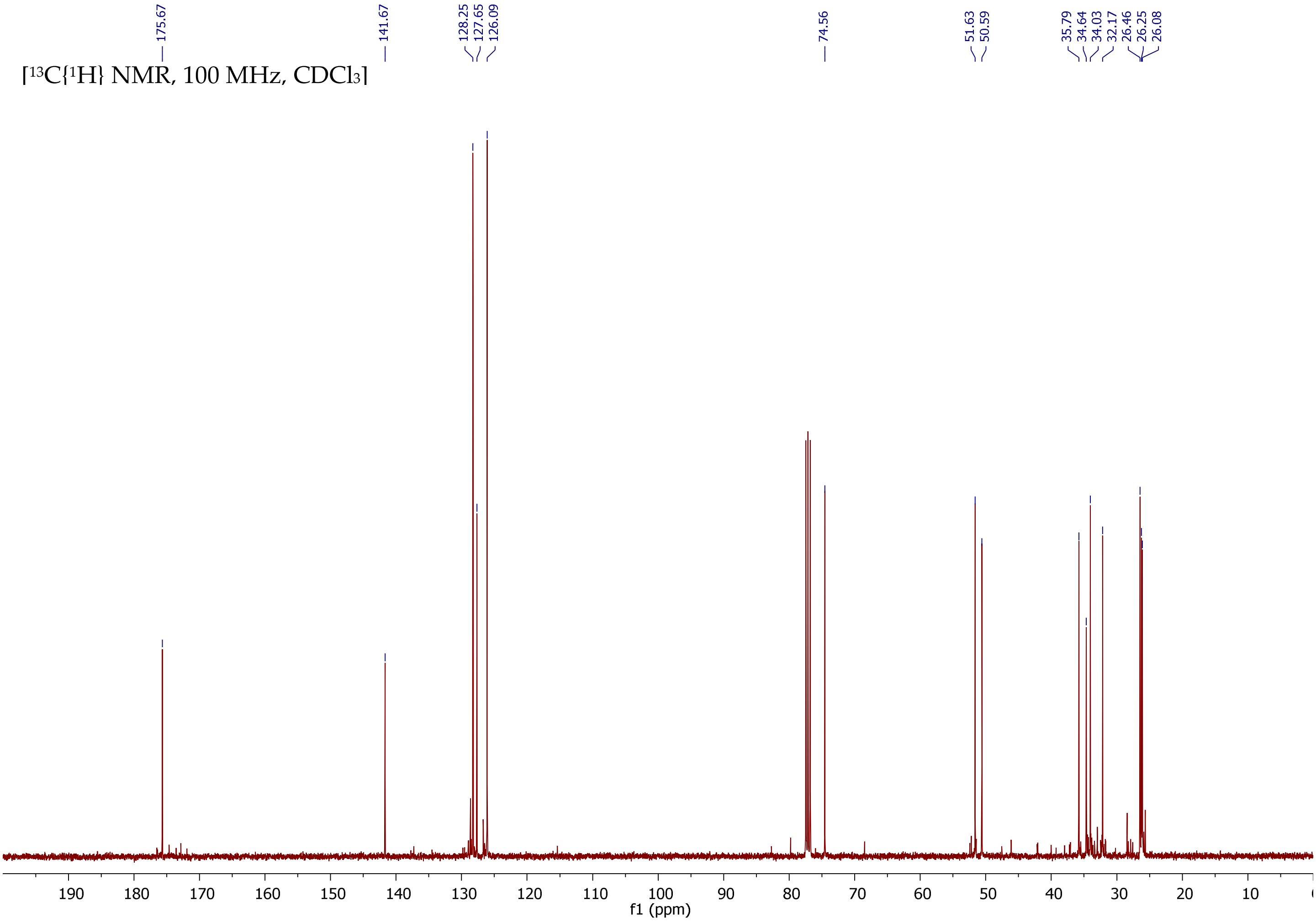

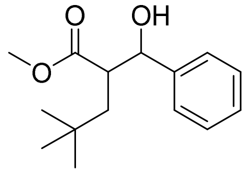

4faa
d.r. = 53:47
[^{1}H NMR, 400 MHz, $CDCl_3$]

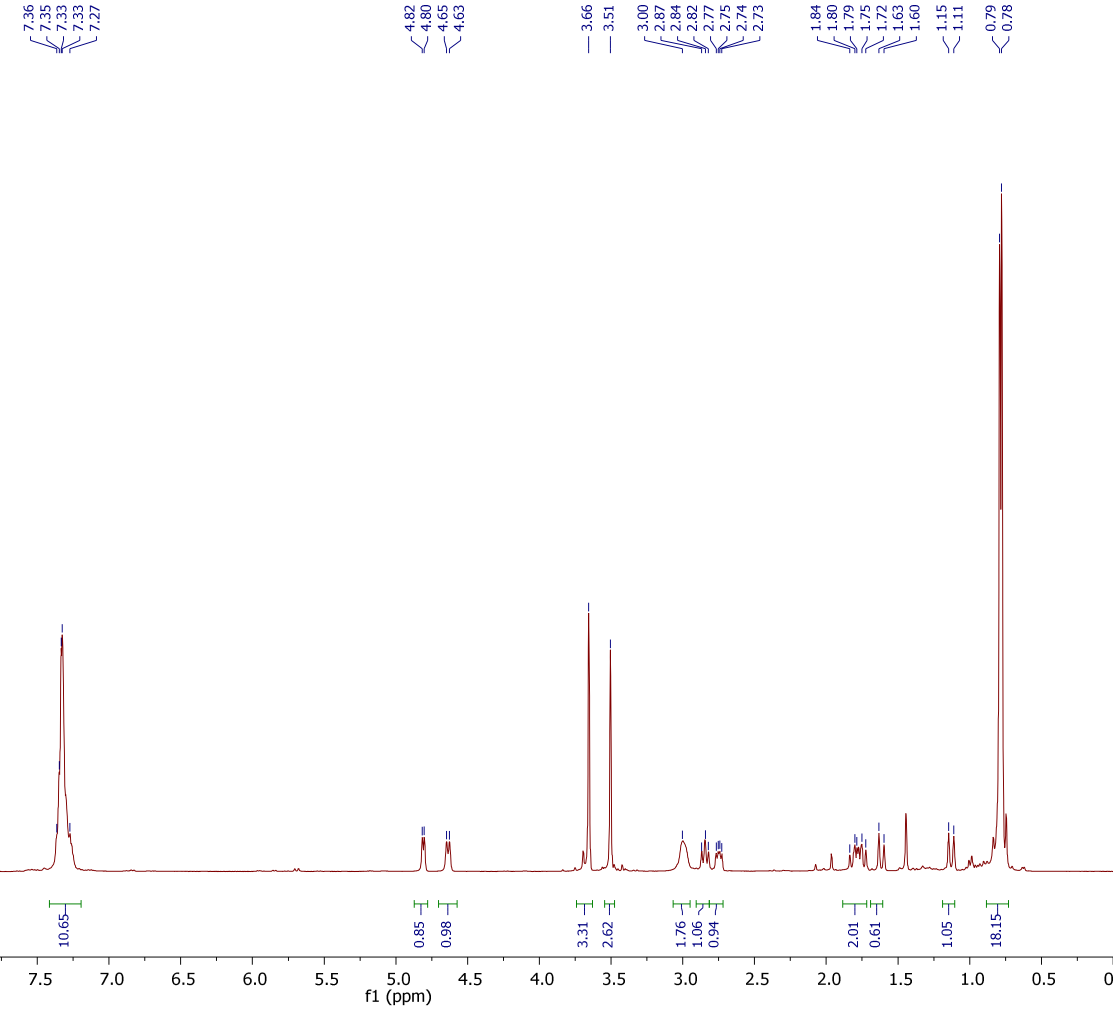

[$^{13}C\{^{1}H\}$ NMR, 100 MHz, $CDCl_3$]

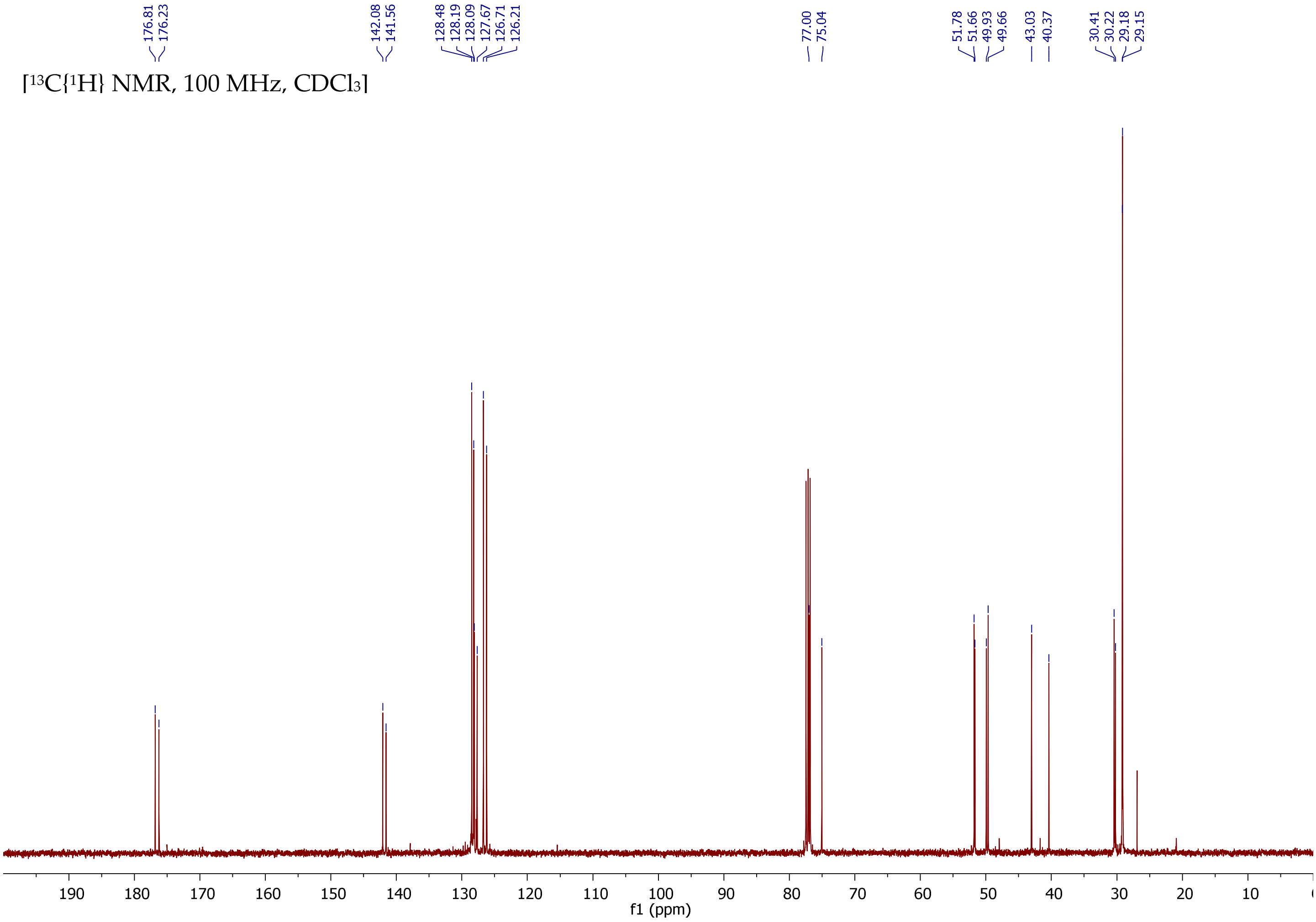

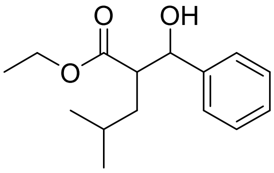

4cba
d.r. = 84:16
[¹H NMR, 400 MHz, CDCl₃]

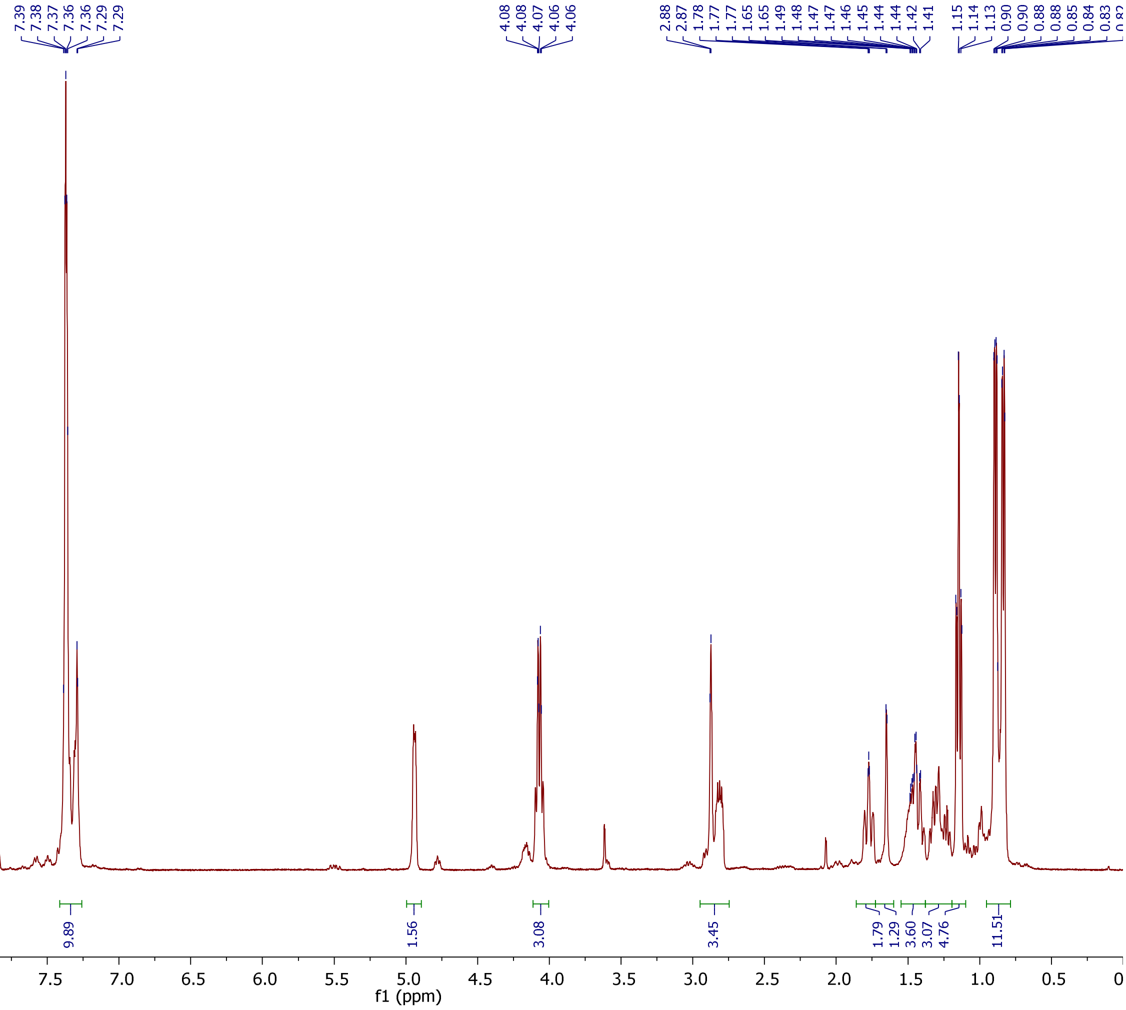

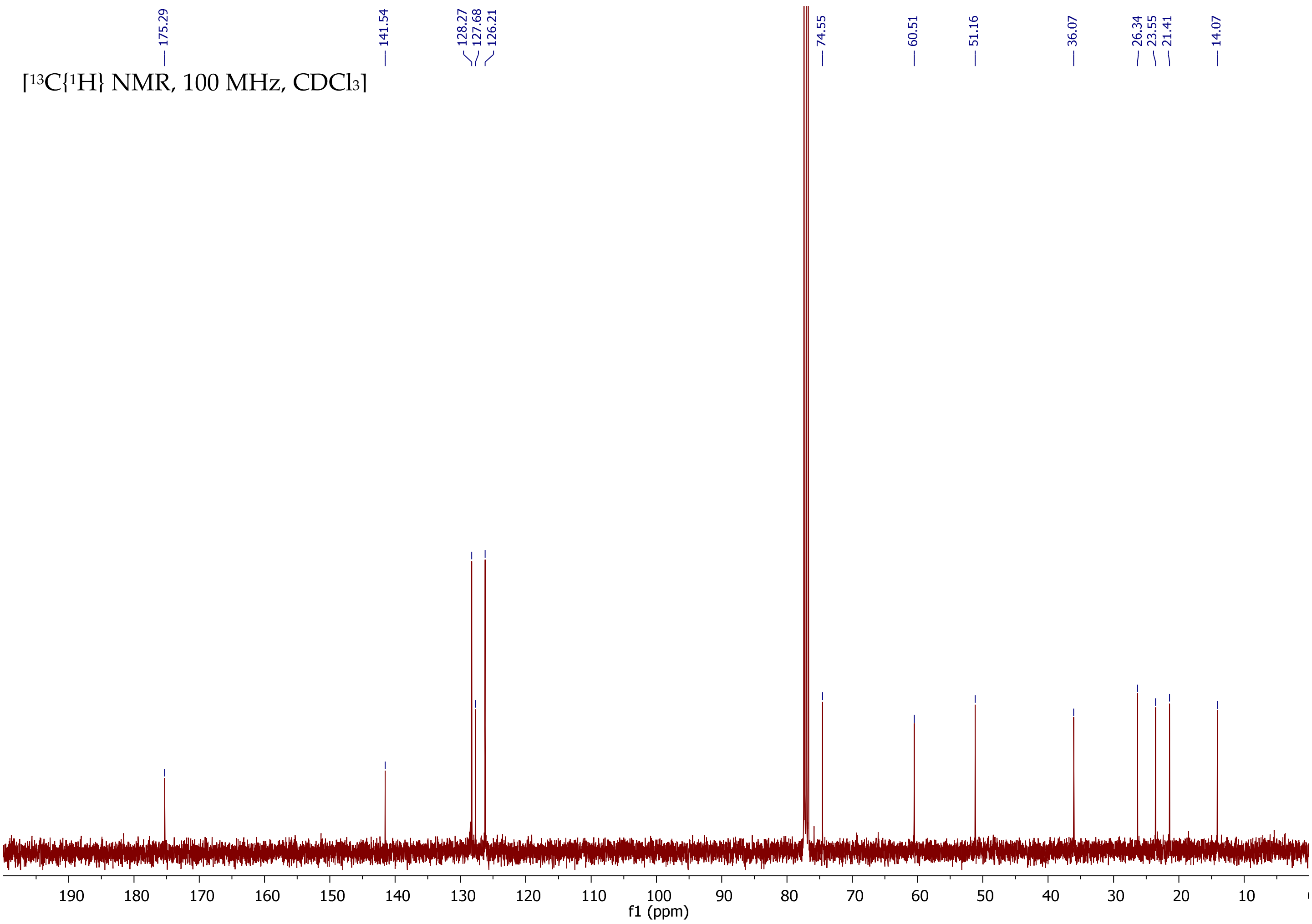
[13C{1H} NMR, 100 MHz, CDCl3]
175.29
141.54
128.27
127.68
126.21
74.55
60.51
51.16
36.07
26.34
23.55
21.41
14.07
f1 (ppm)

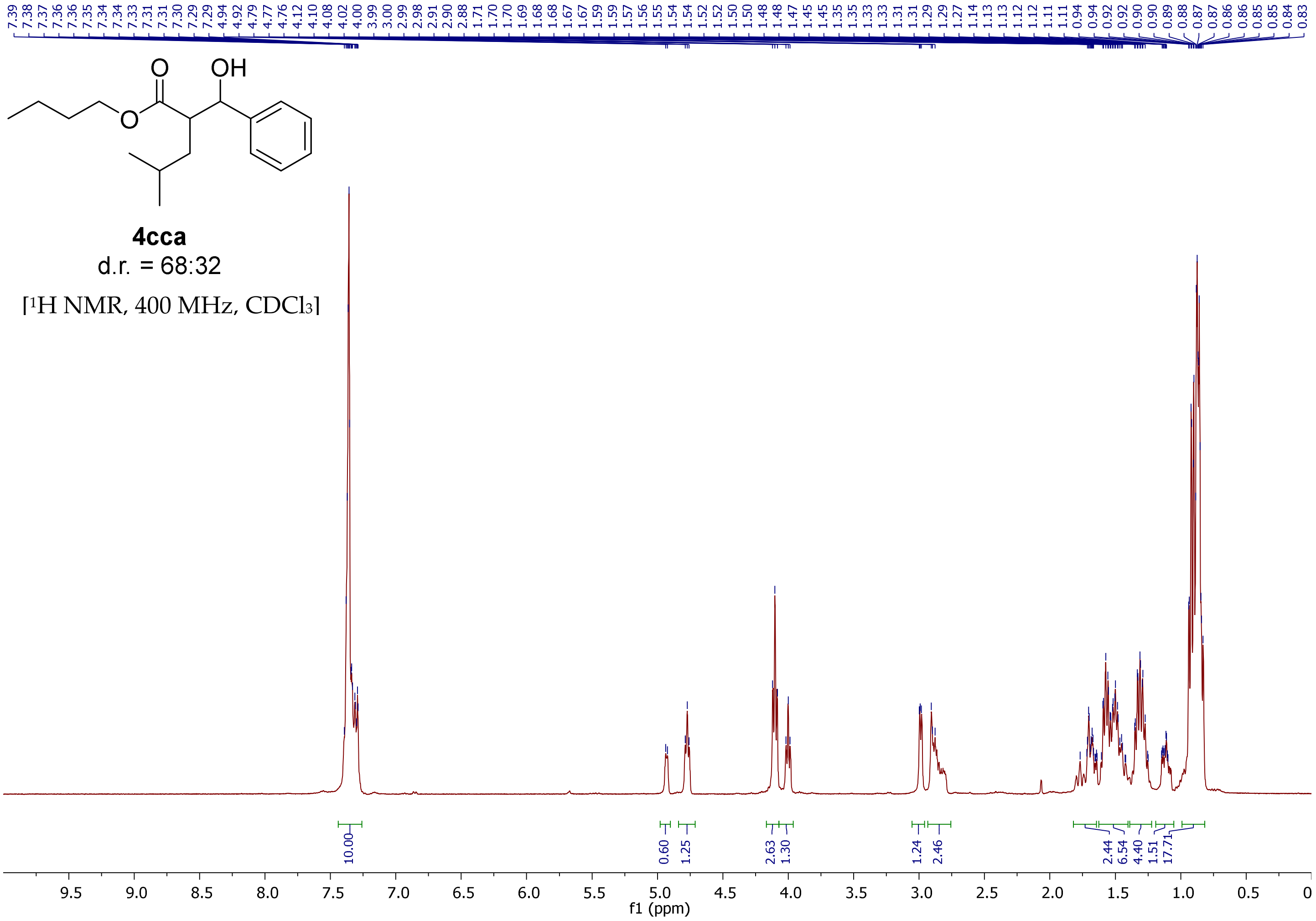
OH
O
O
4cca
d.r. = 68:32
[1H NMR, 400 MHz, CDCl3]
10.00
0.60
1.25
2.63
1.30
1.24
2.46
2.44
6.54
4.40
1.51
17.71
f1 (ppm)

[13C{1H} NMR, 100 MHz, CDCl3]

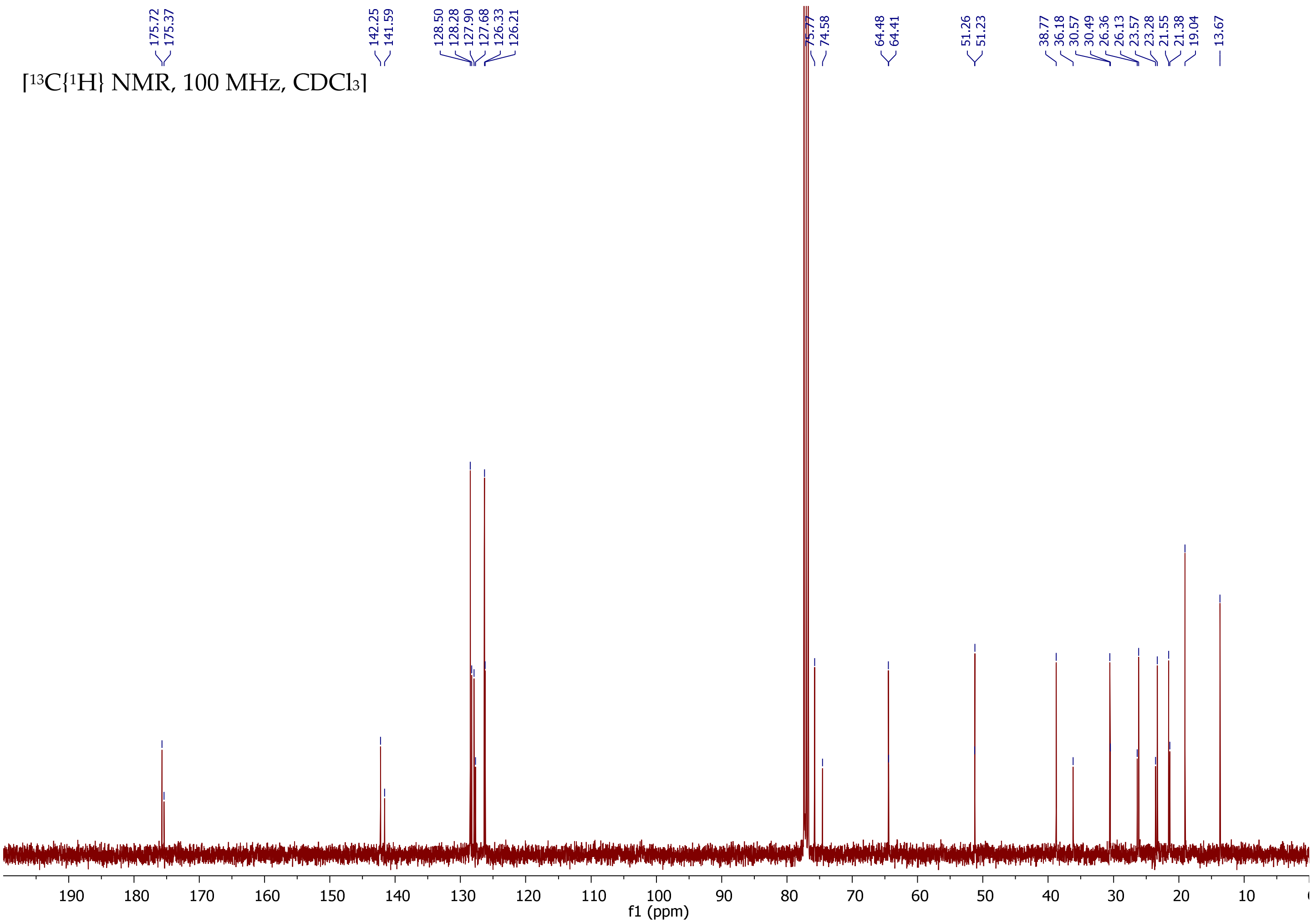

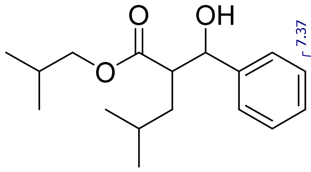

4cda
d.r. = 70:30

[^{1}H NMR, 400 MHz, $CDCl_3$]

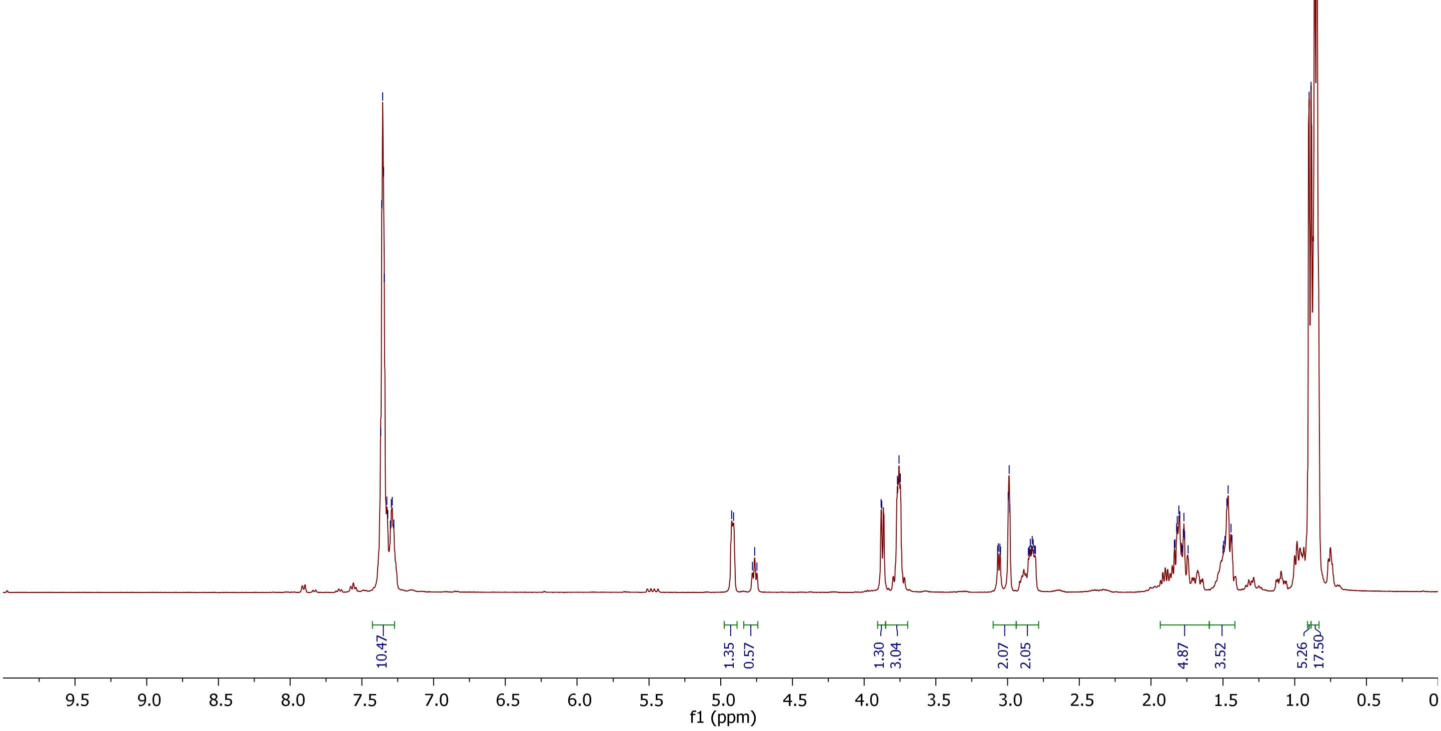

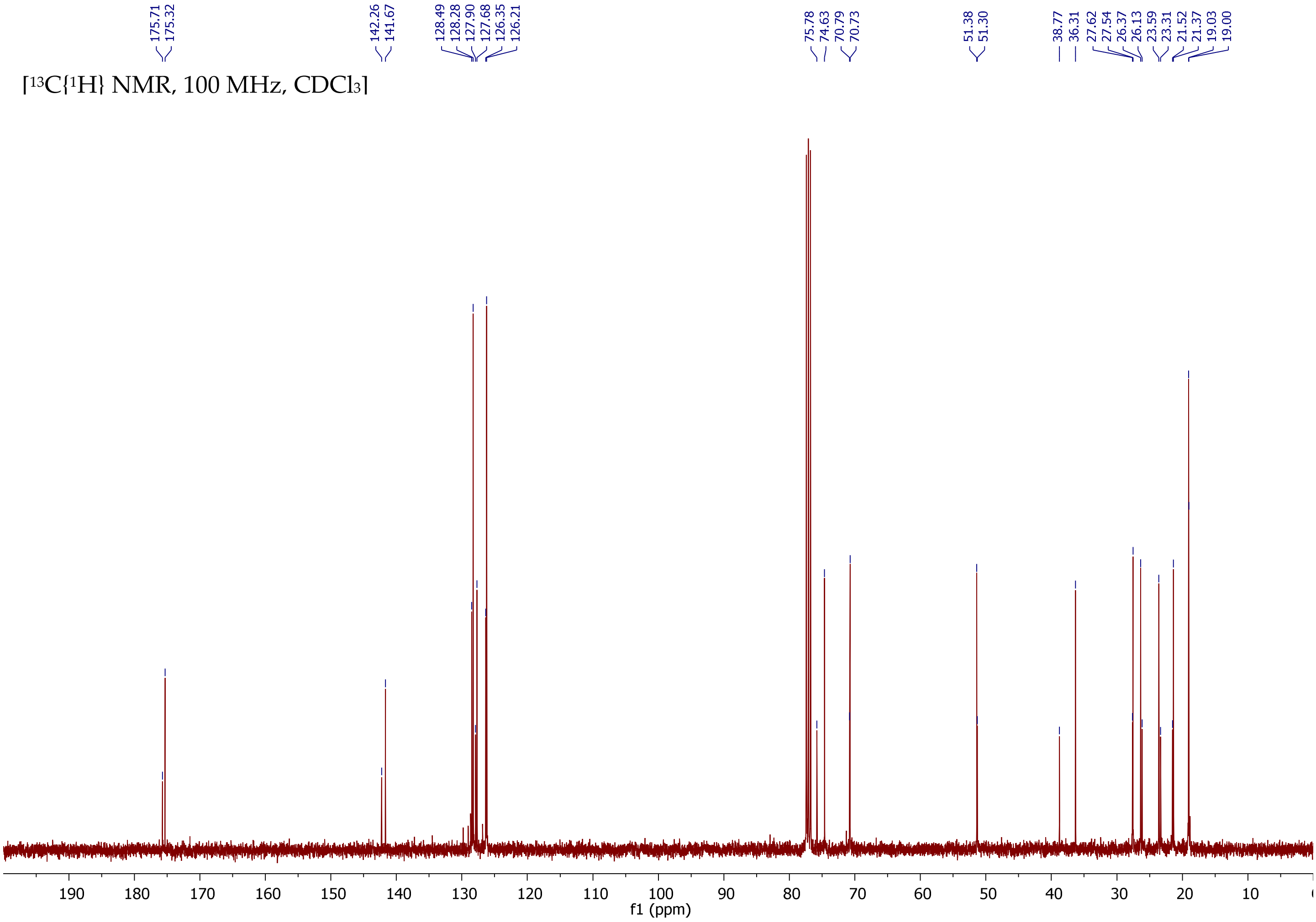
175.71
175.32
142.26
141.67
128.49
128.28
127.90
127.68
126.35
126.21
75.78
74.63
70.79
70.73
51.38
51.30
38.77
36.31
27.62
27.54
26.37
26.13
23.59
23.31
21.52
21.37
19.03
19.00
[13C{1H} NMR, 100 MHz, CDCl3]
190
180
170
160
150
140
130
120
110
100
90
80
70
60
50
40
30
20
10
f1 (ppm)

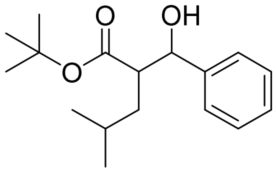

4cea
d.r. = 53:47

[^{1}H NMR, 400 MHz, $CDCl_3$]

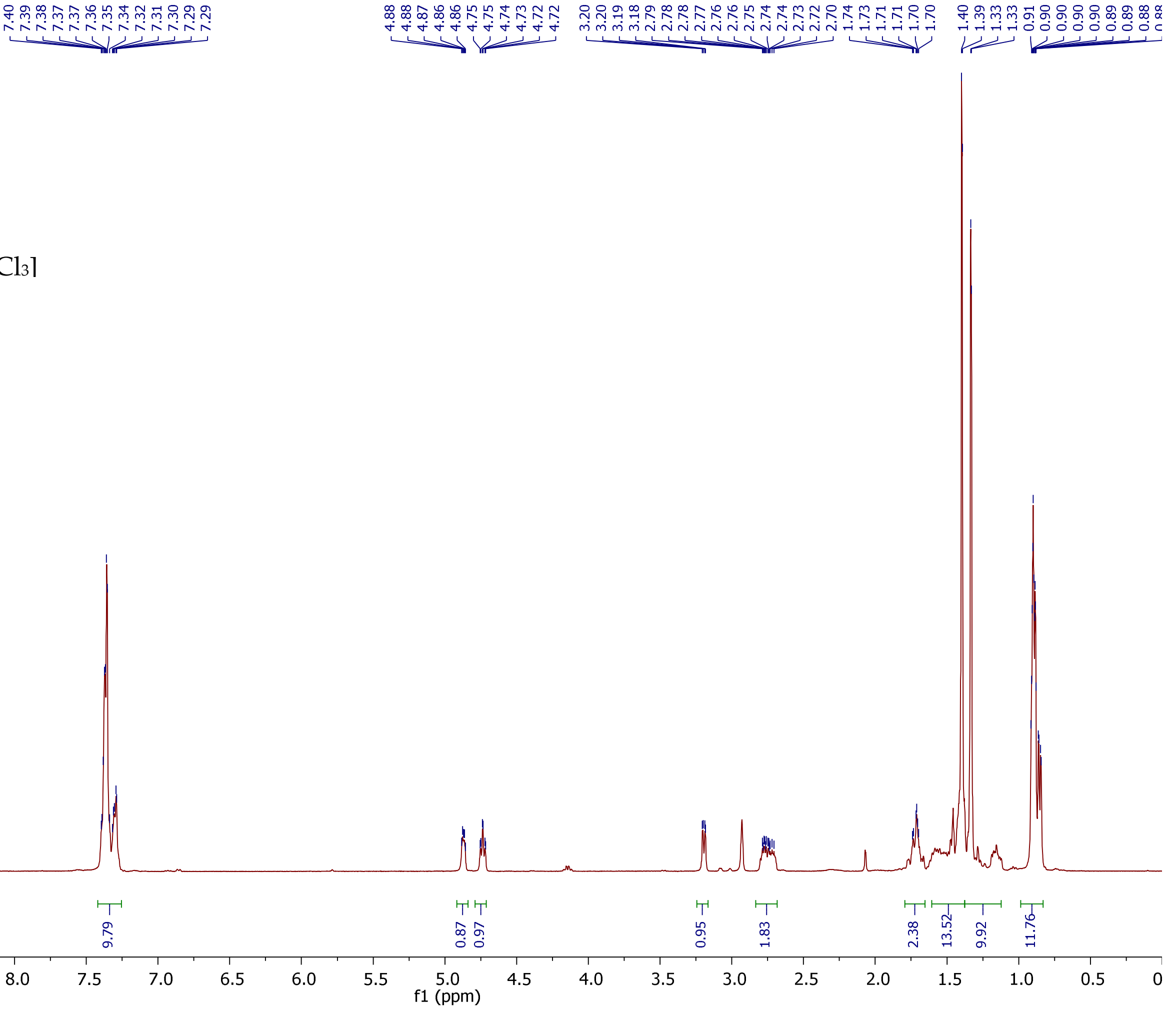

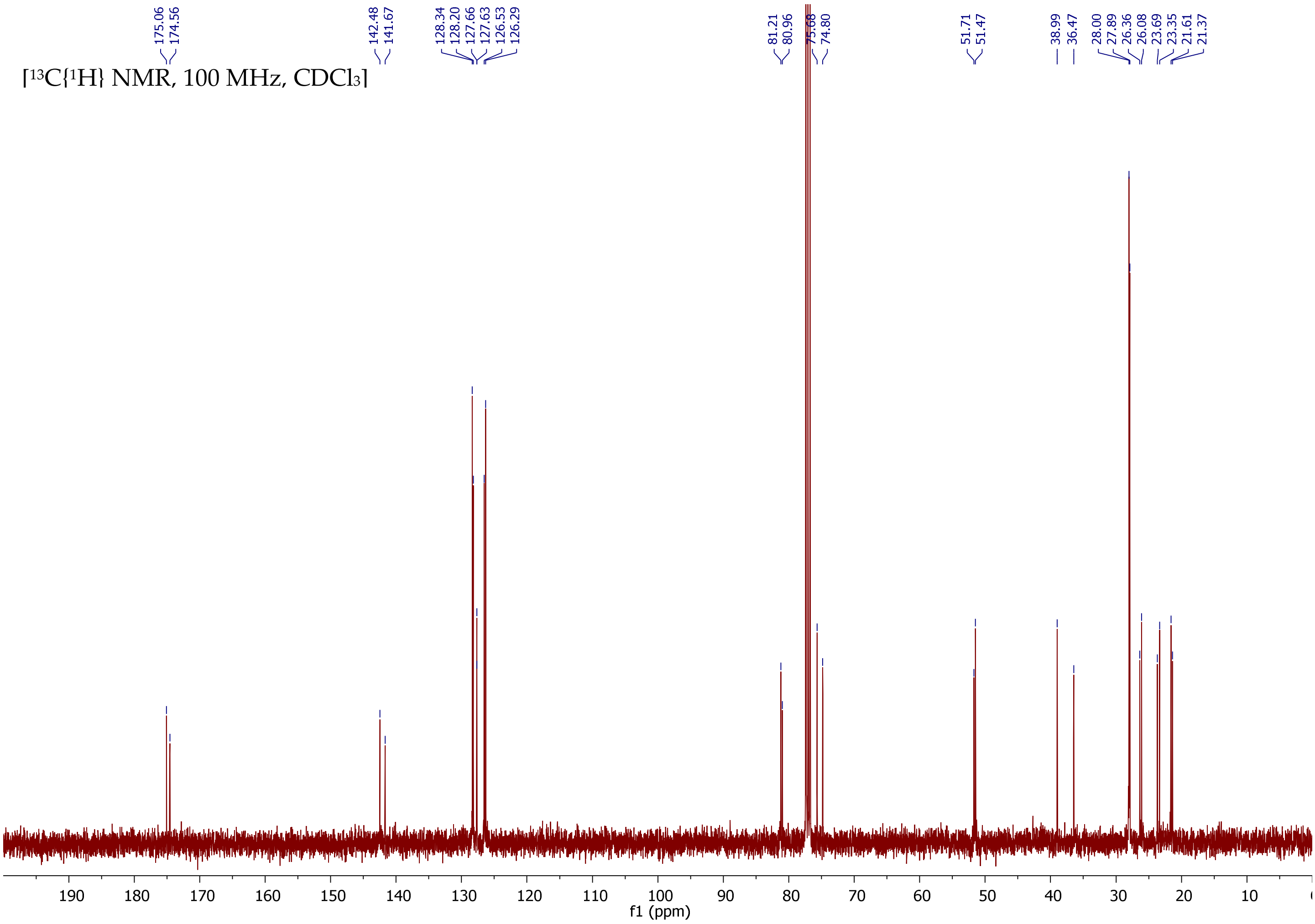
175.06
174.56
142.48
141.67
128.34
128.20
127.66
127.63
126.53
126.29
81.21
80.96
75.68
74.80
51.71
51.47
38.99
36.47
28.00
27.89
26.36
26.08
23.69
23.35
21.61
21.37
[13C{1H} NMR, 100 MHz, CDCl3]
190
180
170
160
150
140
130
120
110
100
90
80
70
60
50
40
30
20
10
f1 (ppm)

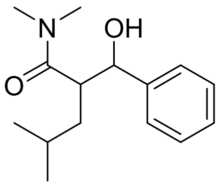

4cga
d.r. > 95:5

[^{1}H NMR, 400 MHz, $CDCl_3$]

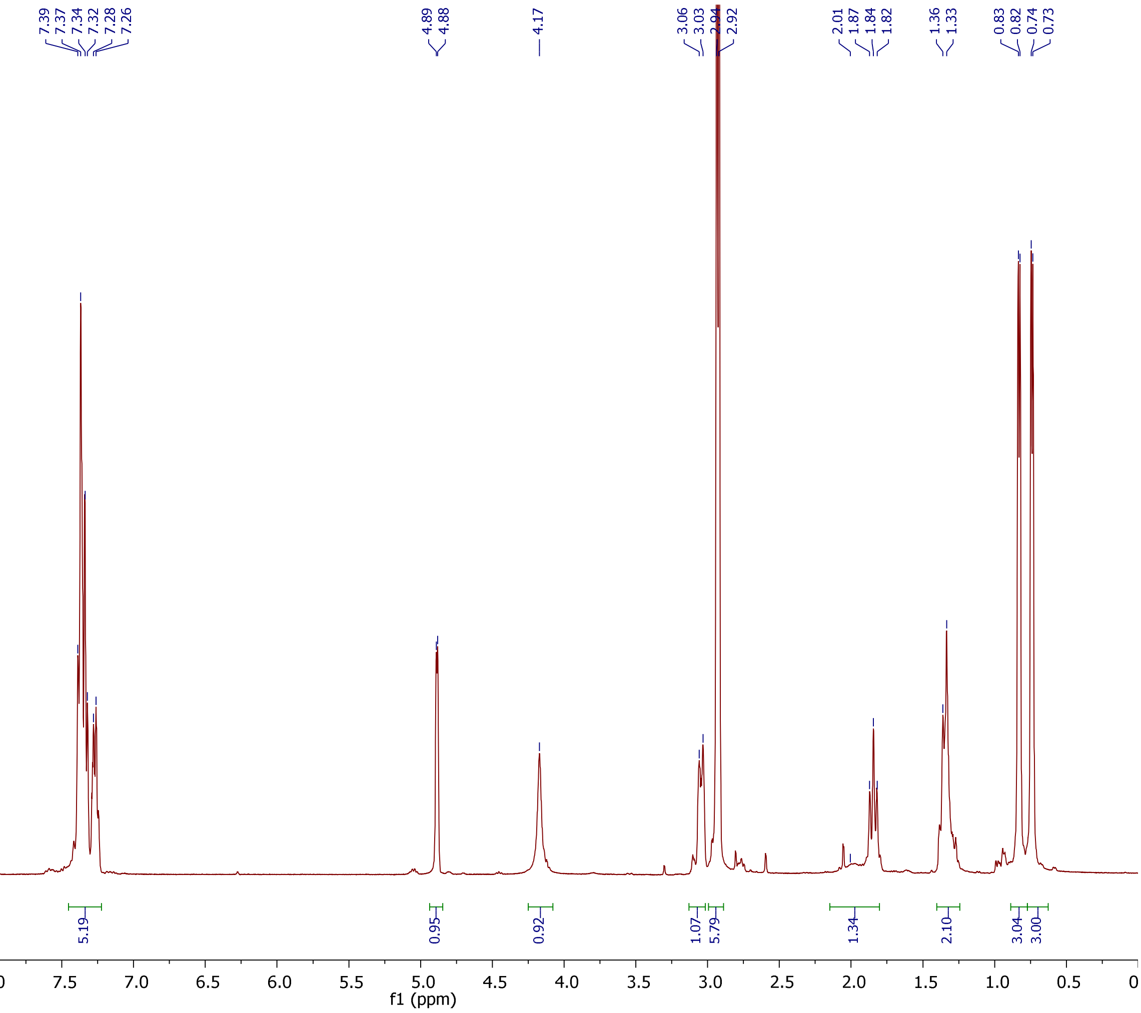

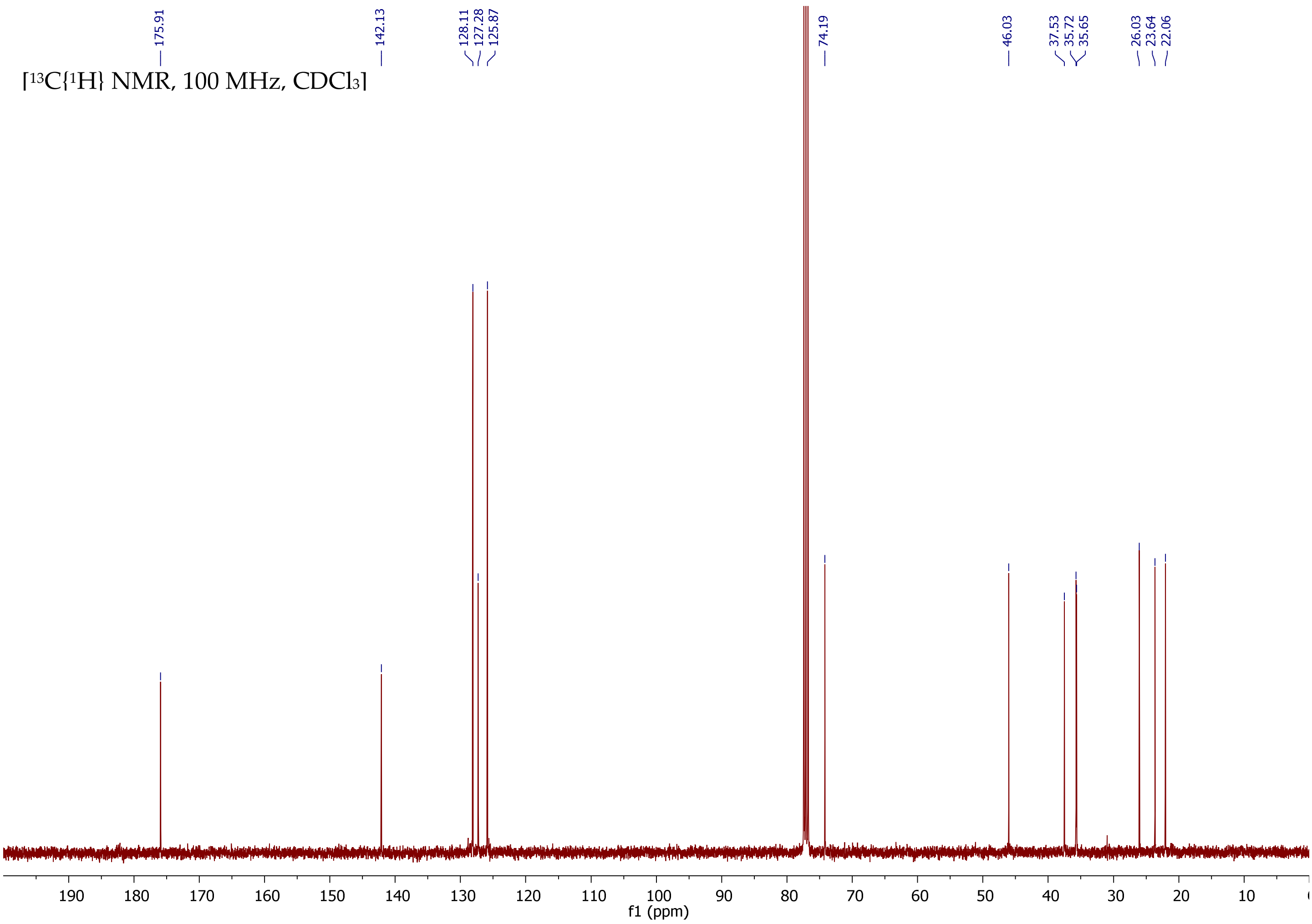
[13C{1H} NMR, 100 MHz, CDCl3]
175.91
142.13
128.11
127.28
125.87
74.19
46.03
37.53
35.72
35.65
26.03
23.64
22.06
190 180 170 160 150 140 130 120 110 100 90 80 70 60 50 40 30 20 10
f1 (ppm)

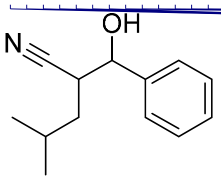

4cha
d.r. = 56:44
[¹H NMR, 400 MHz, $CDCl_3$]

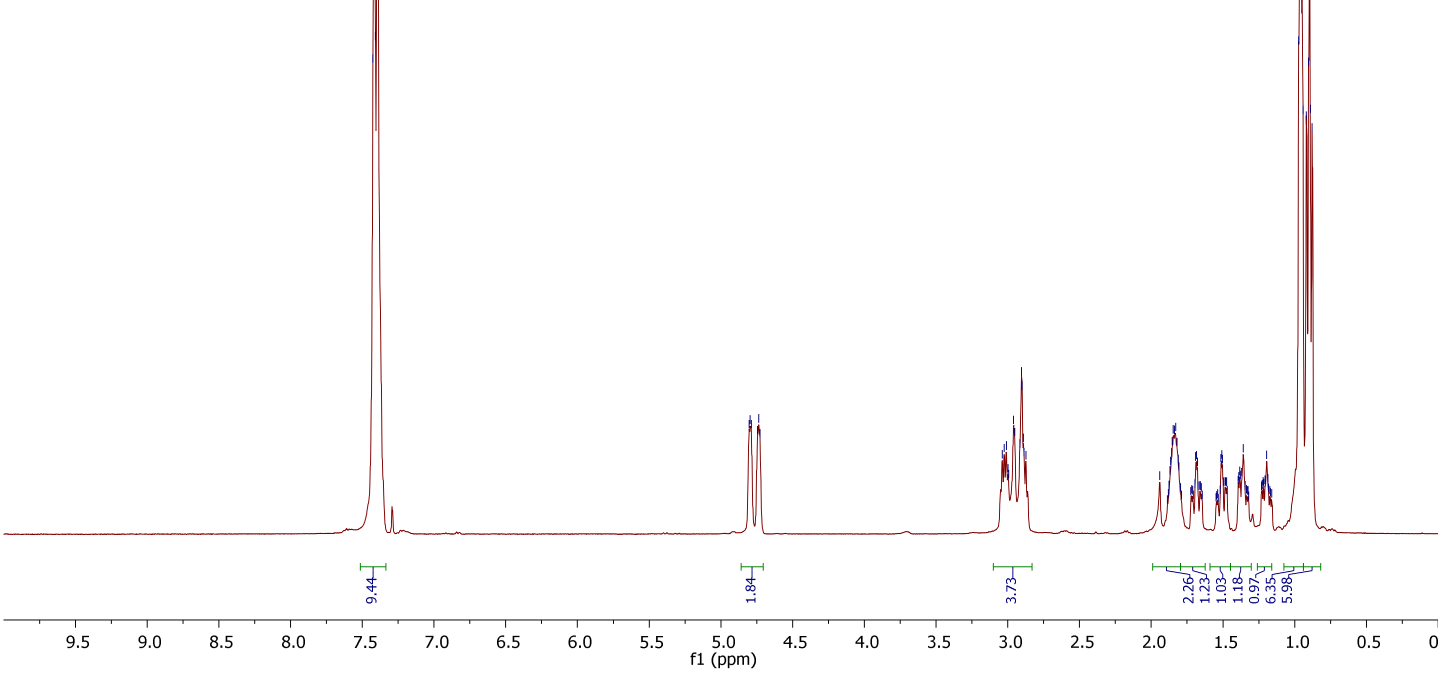

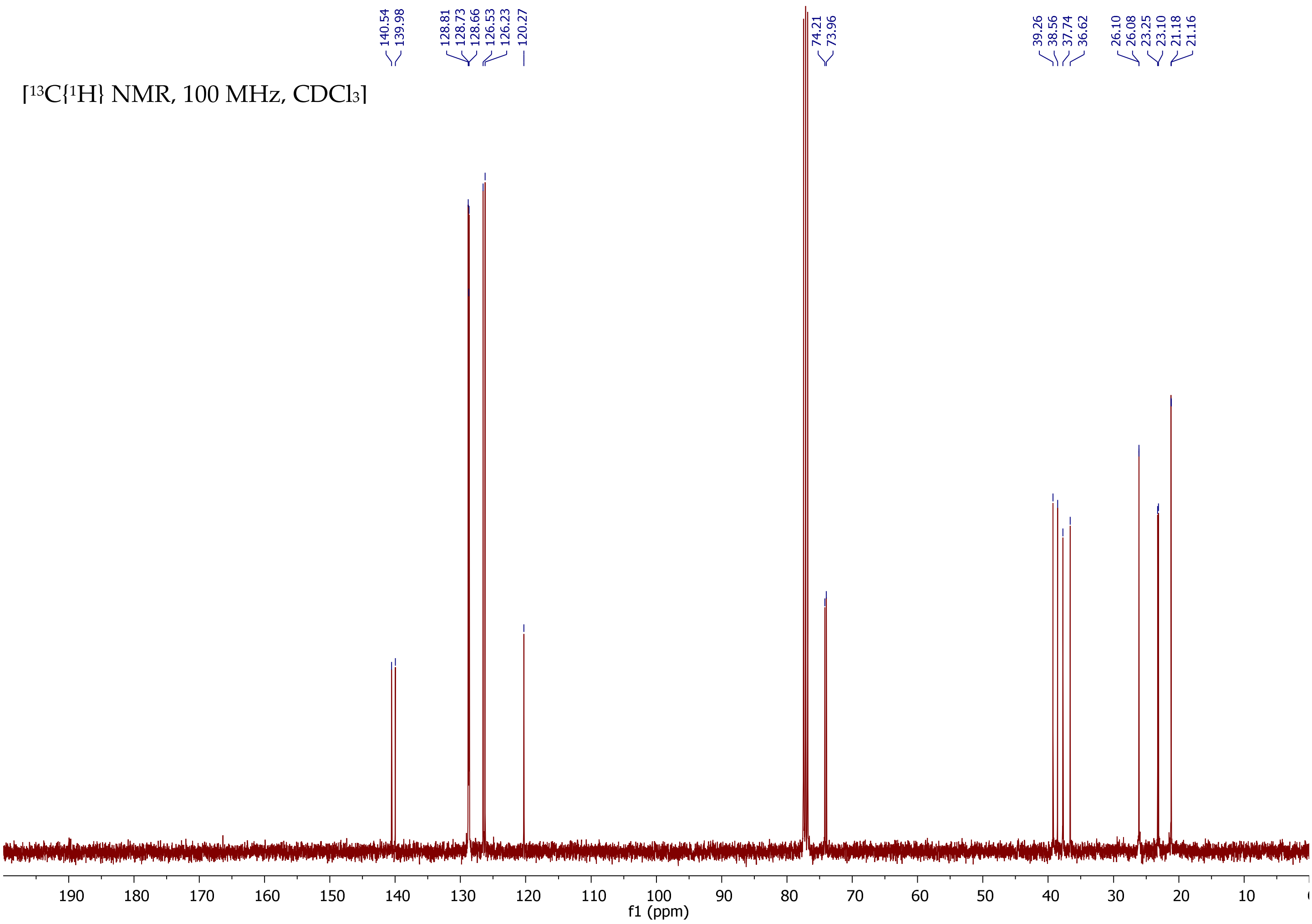
140.54
139.98
128.81
128.73
128.66
126.53
126.23
120.27
74.21
73.96
39.26
38.56
37.74
36.62
26.10
26.08
23.25
23.10
21.18
21.16
[13C{1H} NMR, 100 MHz, CDCl3]
190
180
170
160
150
140
130
120
110
100
90
80
70
60
50
40
30
20
10
f1 (ppm)

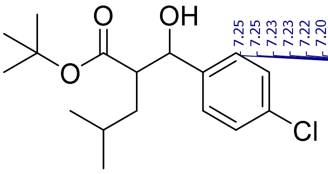

4ceb

d.r. = 72:28

[1H NMR, 400 MHz, CDCl3]

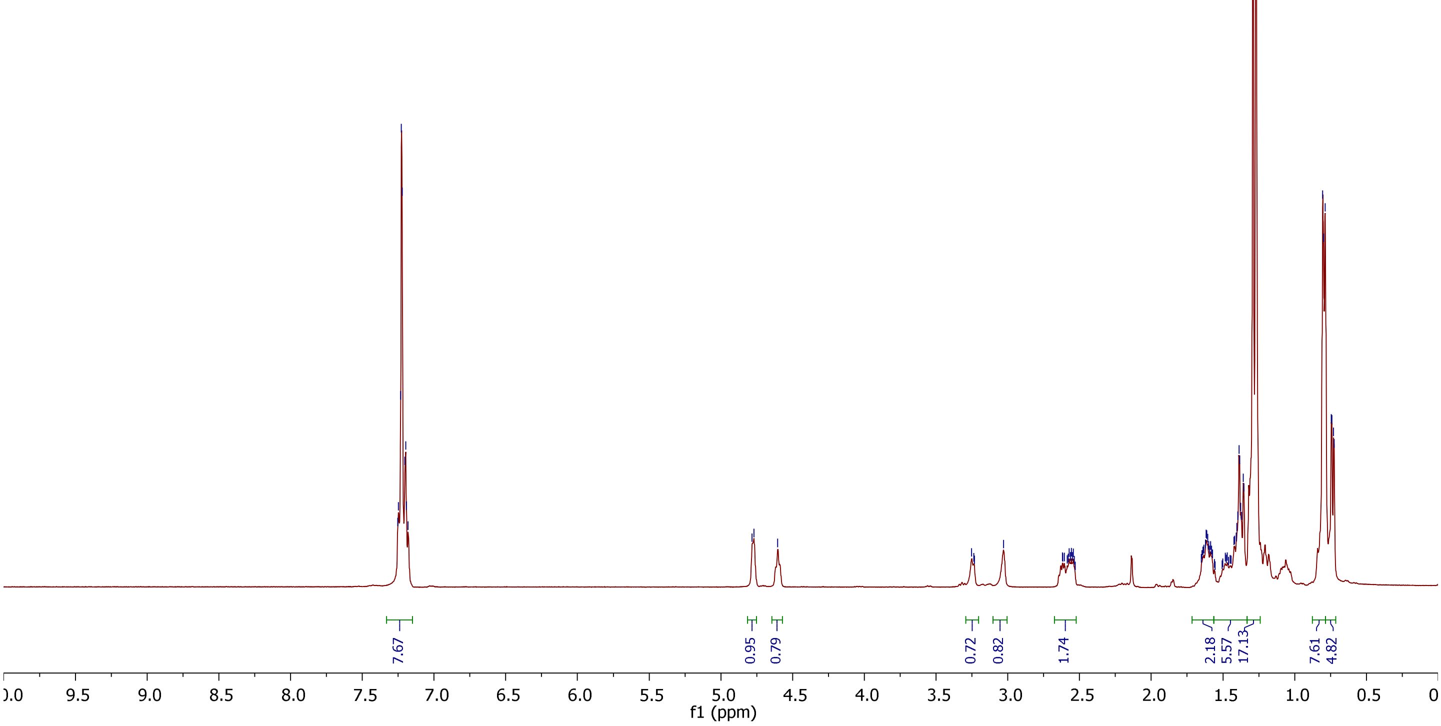

[13C{1H} NMR, 100 MHz, CDCl3]

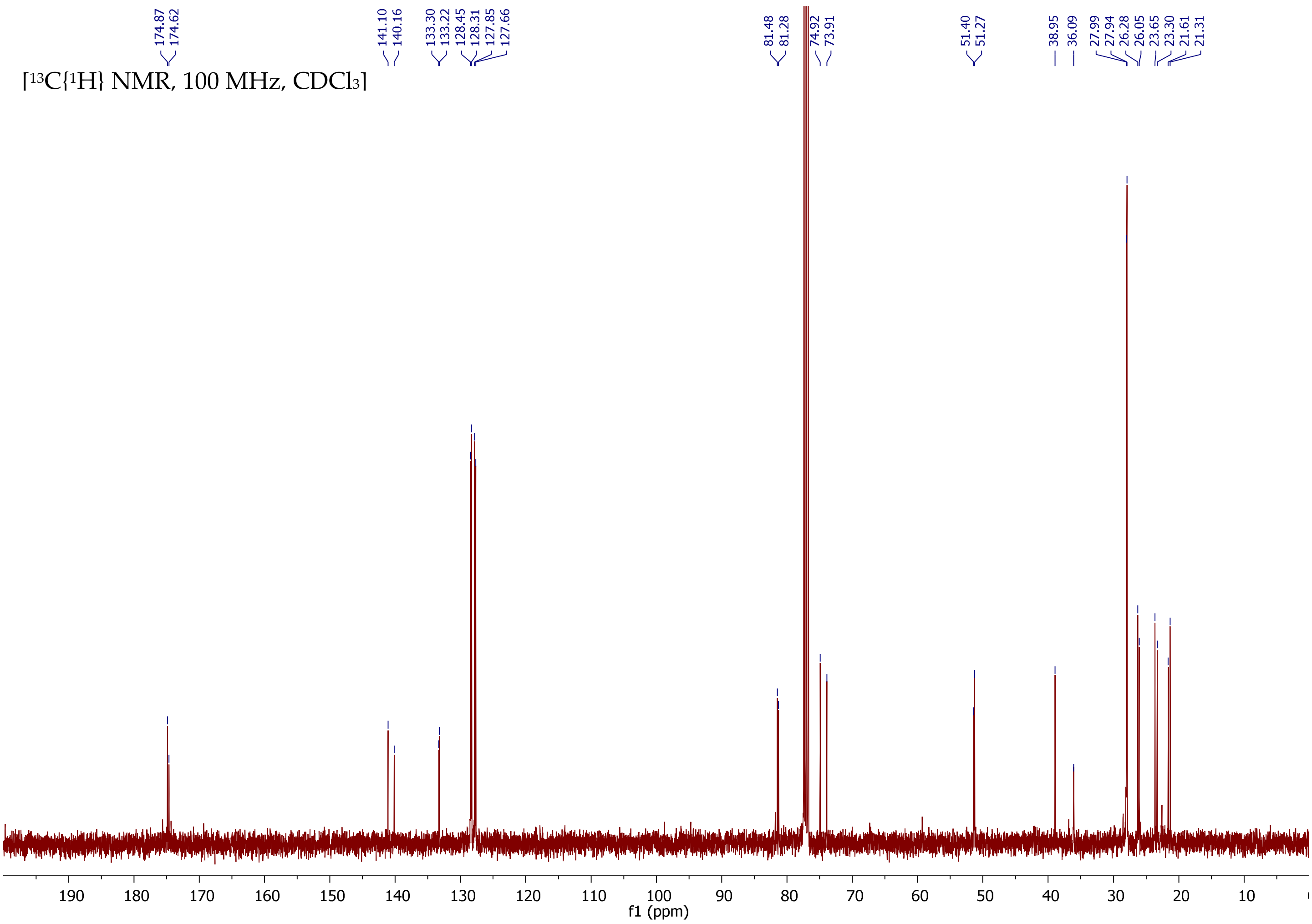

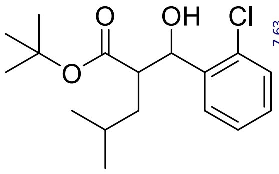

4cec
d.r. = 52:48

[^{1}H NMR, 400 MHz, $CDCl_3$]

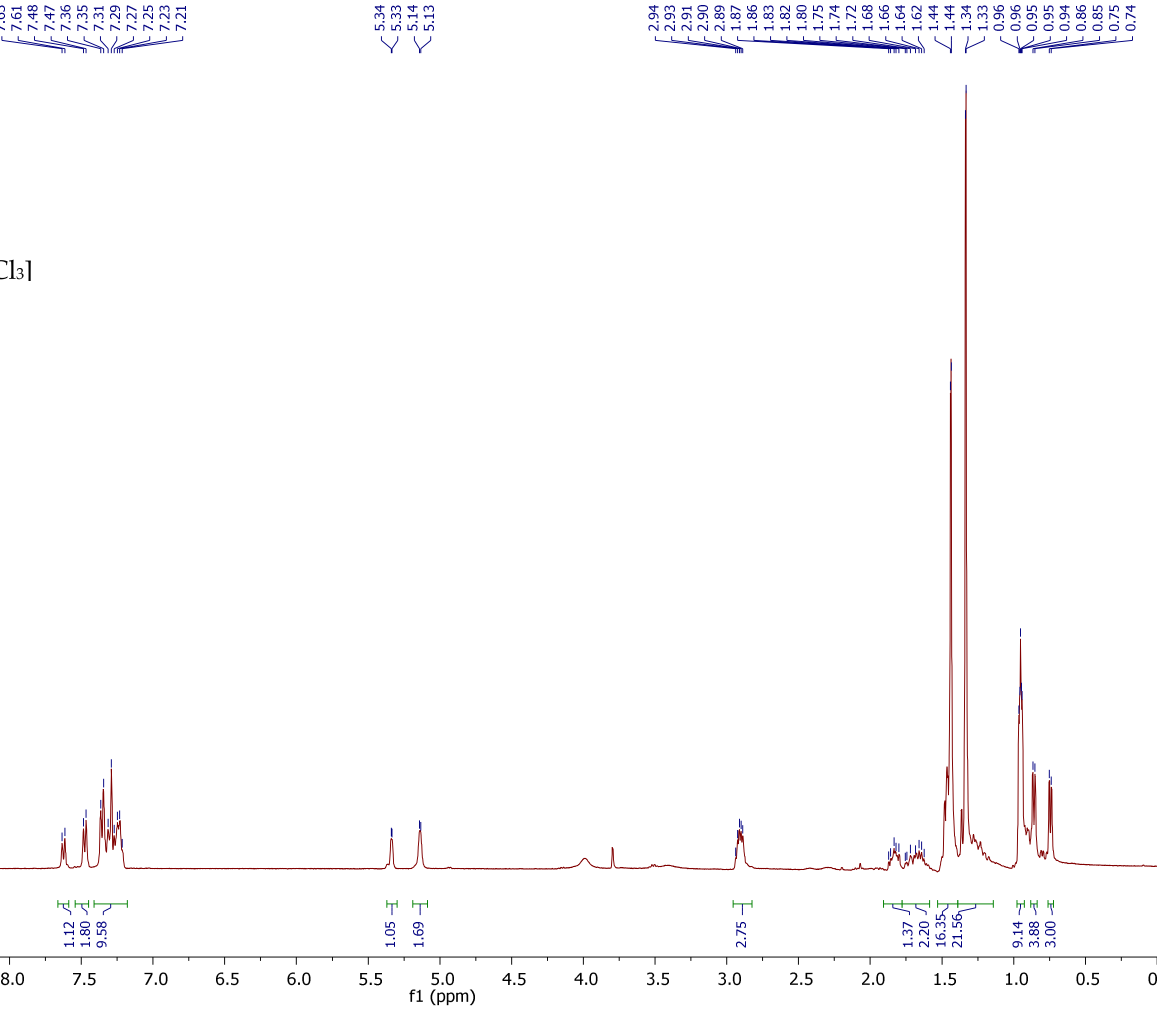

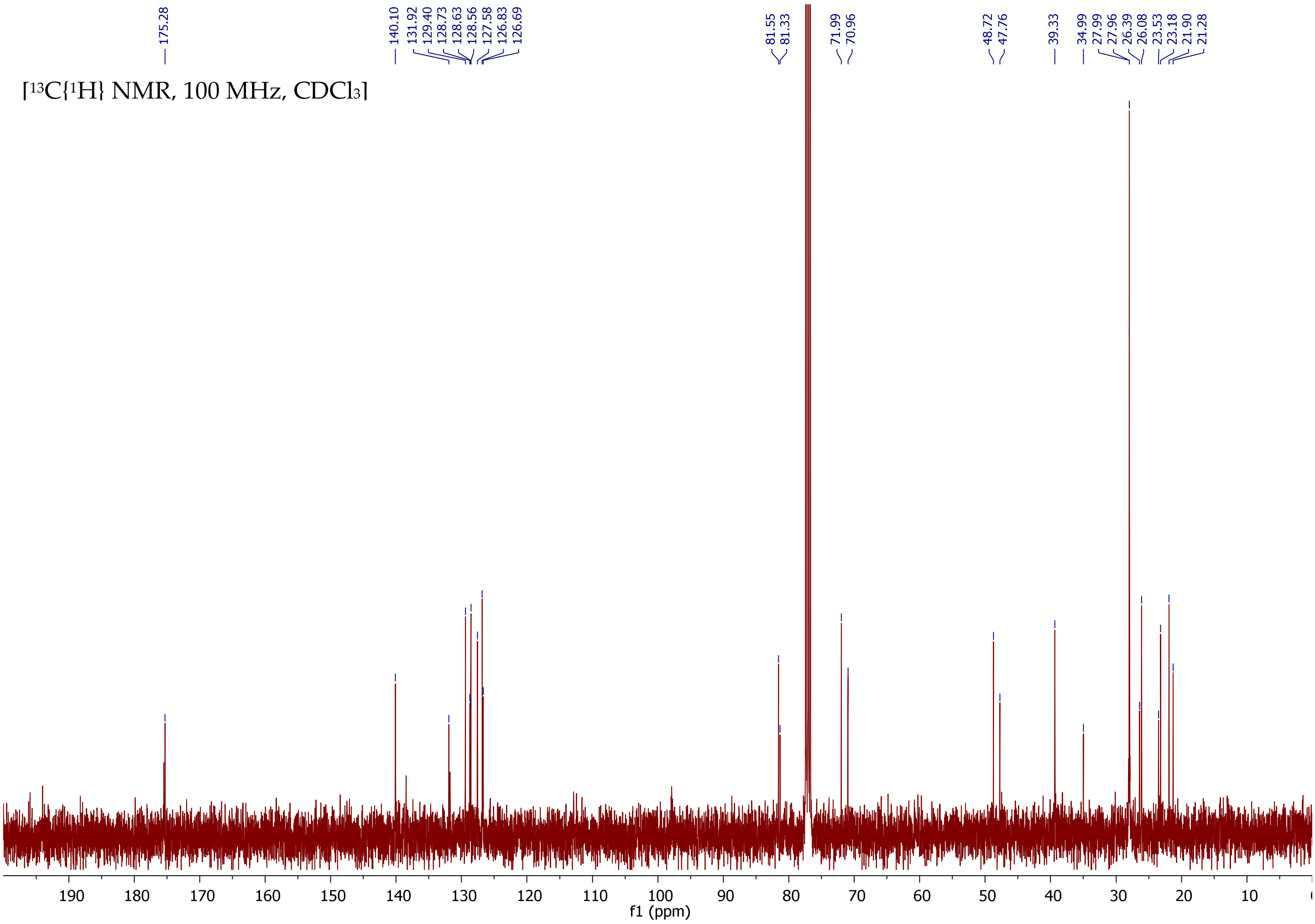

175.28
140.10
131.92
129.40
128.73
128.63
128.56
127.58
126.83
126.69
81.55
81.33
71.99
70.96
48.72
47.76
39.33
34.99
27.99
27.96
26.39
26.08
23.53
23.18
21.90
21.28
[13C{1H} NMR, 100 MHz, CDCl3]
190
180
170
160
150
140
130
120
110
100
90
80
70
60
50
40
30
20
10
f1 (ppm)

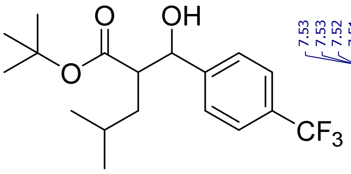

4ced
d.r. = 58:42

[1H NMR, 400 MHz, CDCl3]

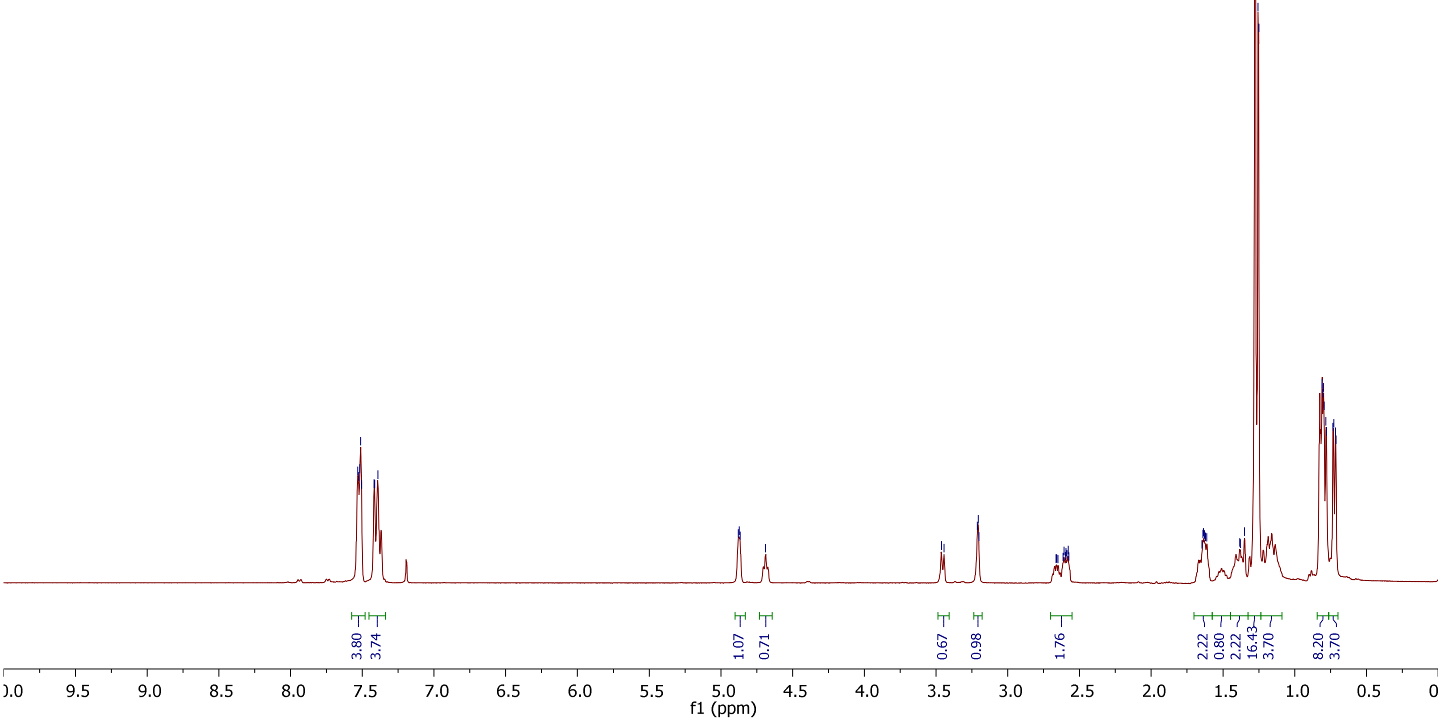

[$^{13}C\{^{1}H\}$ NMR, 100 MHz, $CDCl_3$]

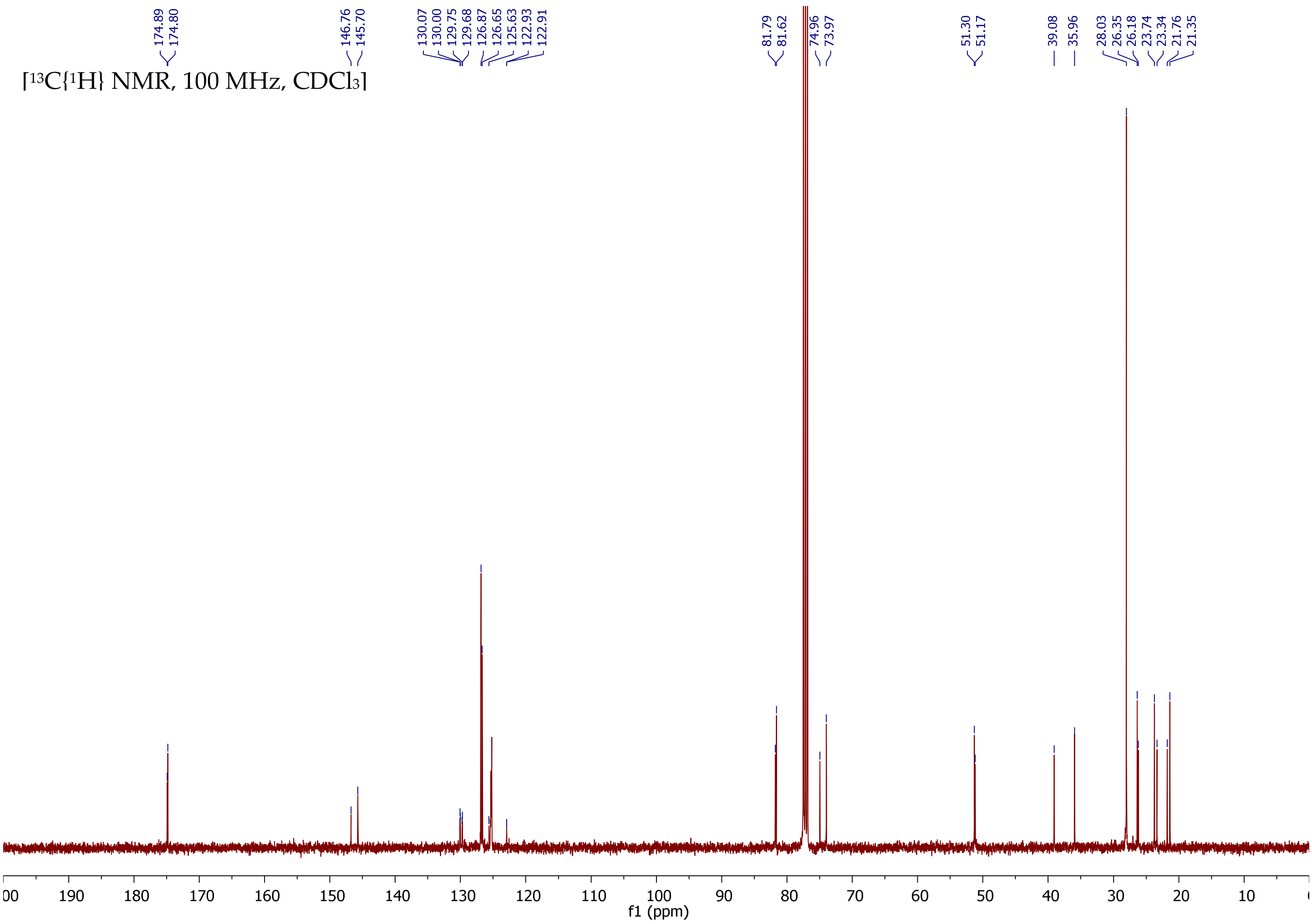

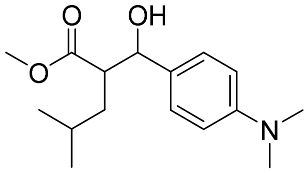

4cae
d.r. = 71:29
[¹H NMR, 400 MHz, CDCl3]

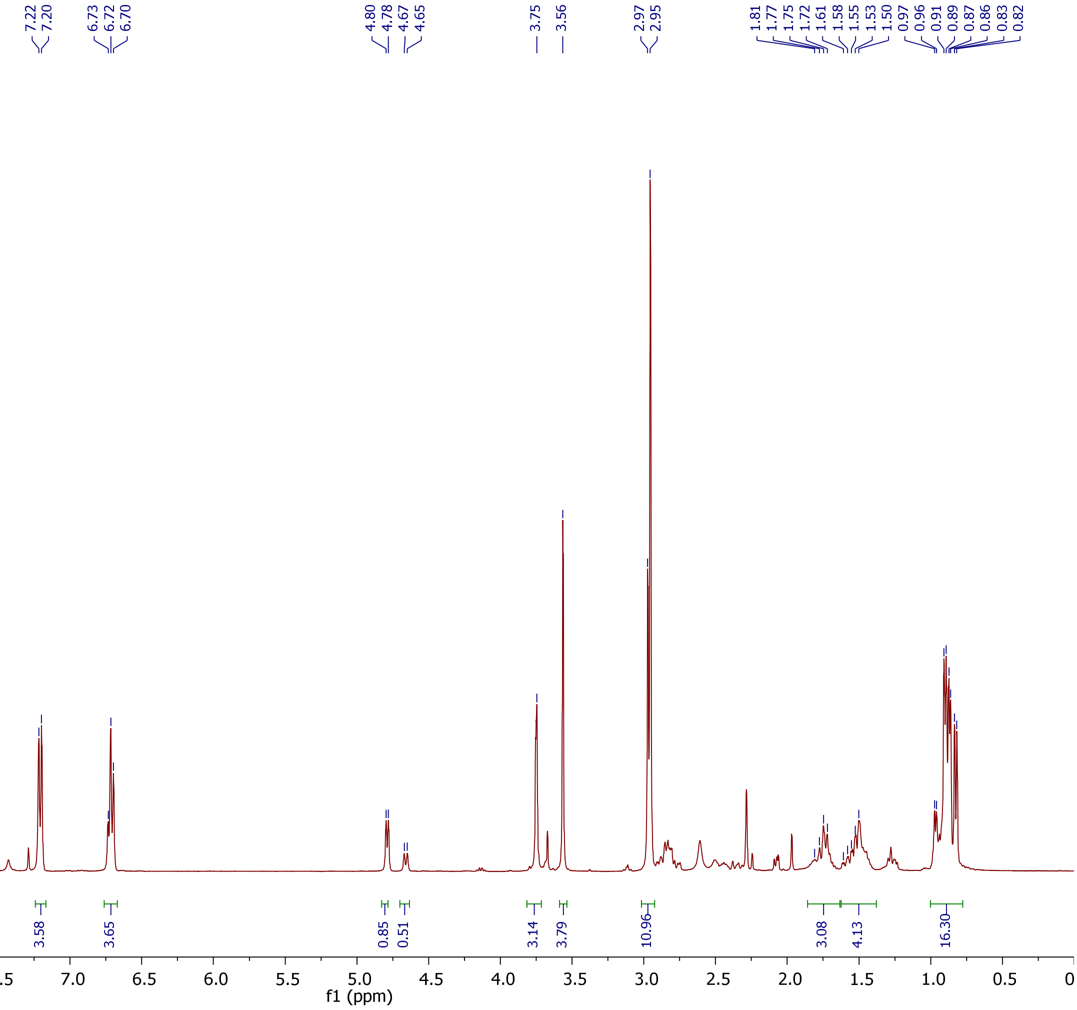

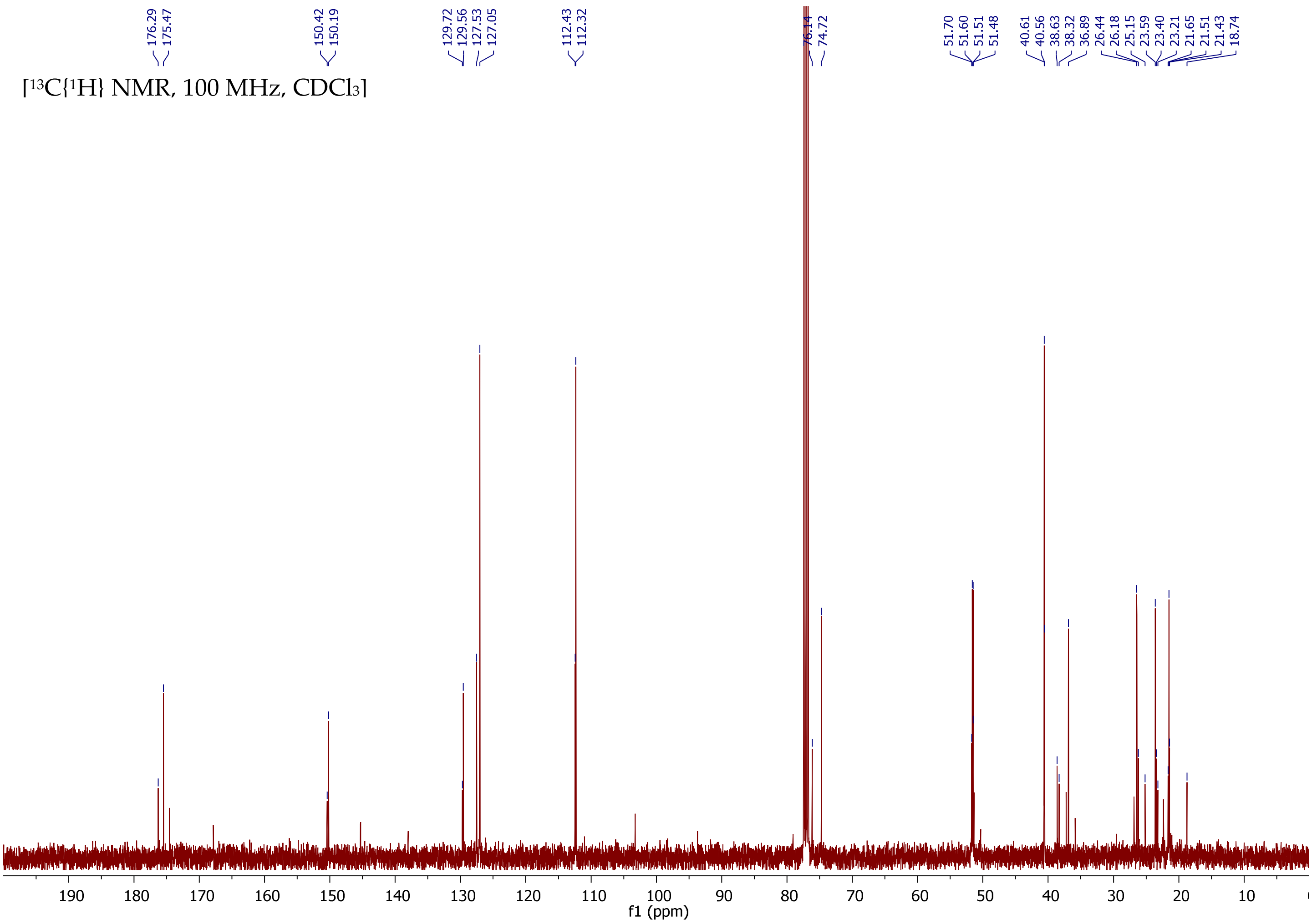
[13C{1H} NMR, 100 MHz, CDCl3]
176.29
175.47
150.42
150.19
129.72
129.56
127.53
127.05
112.43
112.32
76.14
74.72
51.70
51.60
51.51
51.48
40.61
40.56
38.63
38.32
36.89
26.44
26.18
25.15
23.59
23.40
23.21
21.65
21.51
21.43
18.74
190 180 170 160 150 140 130 120 110 100 90 80 70 60 50 40 30 20 10
f1 (ppm)

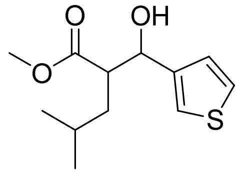

4caf
d.r. = 83:17

[1H NMR, 400 MHz, CDCl3]

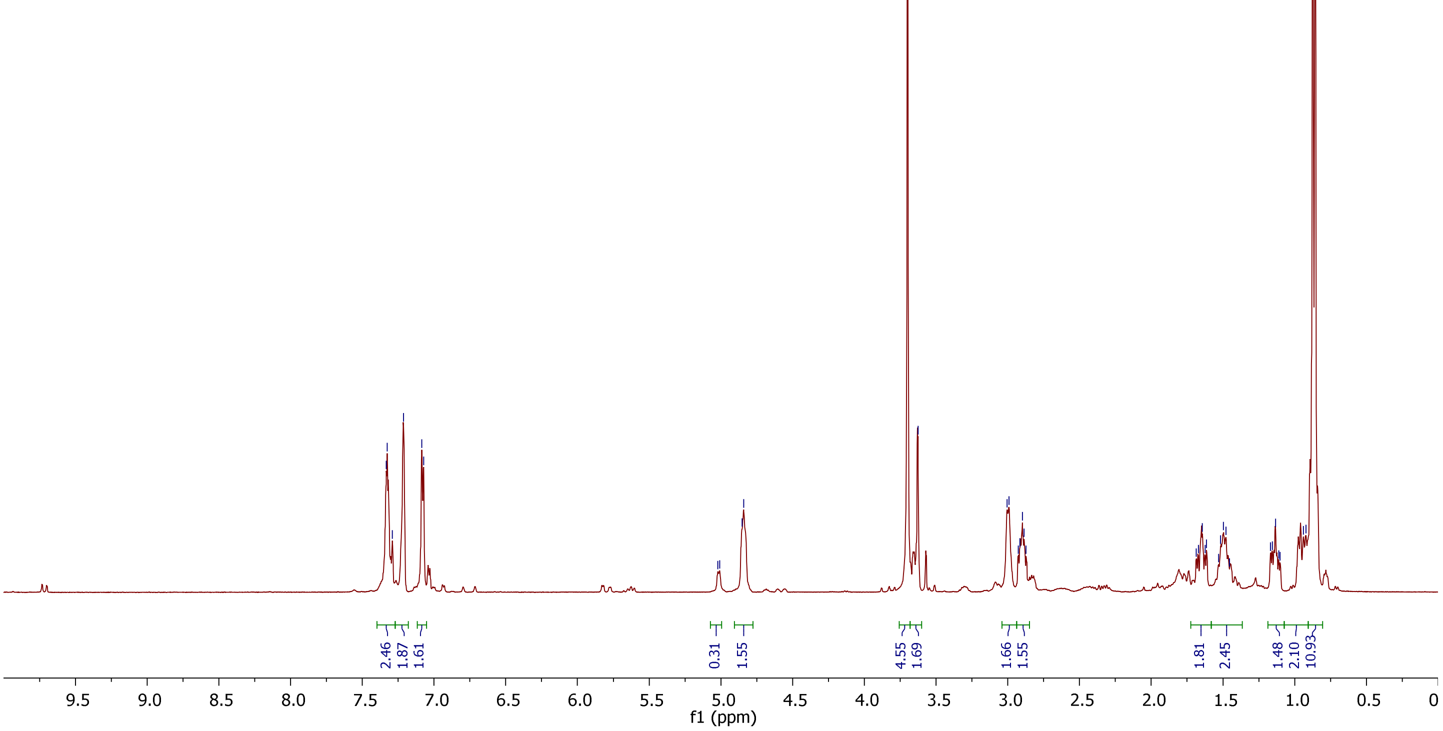

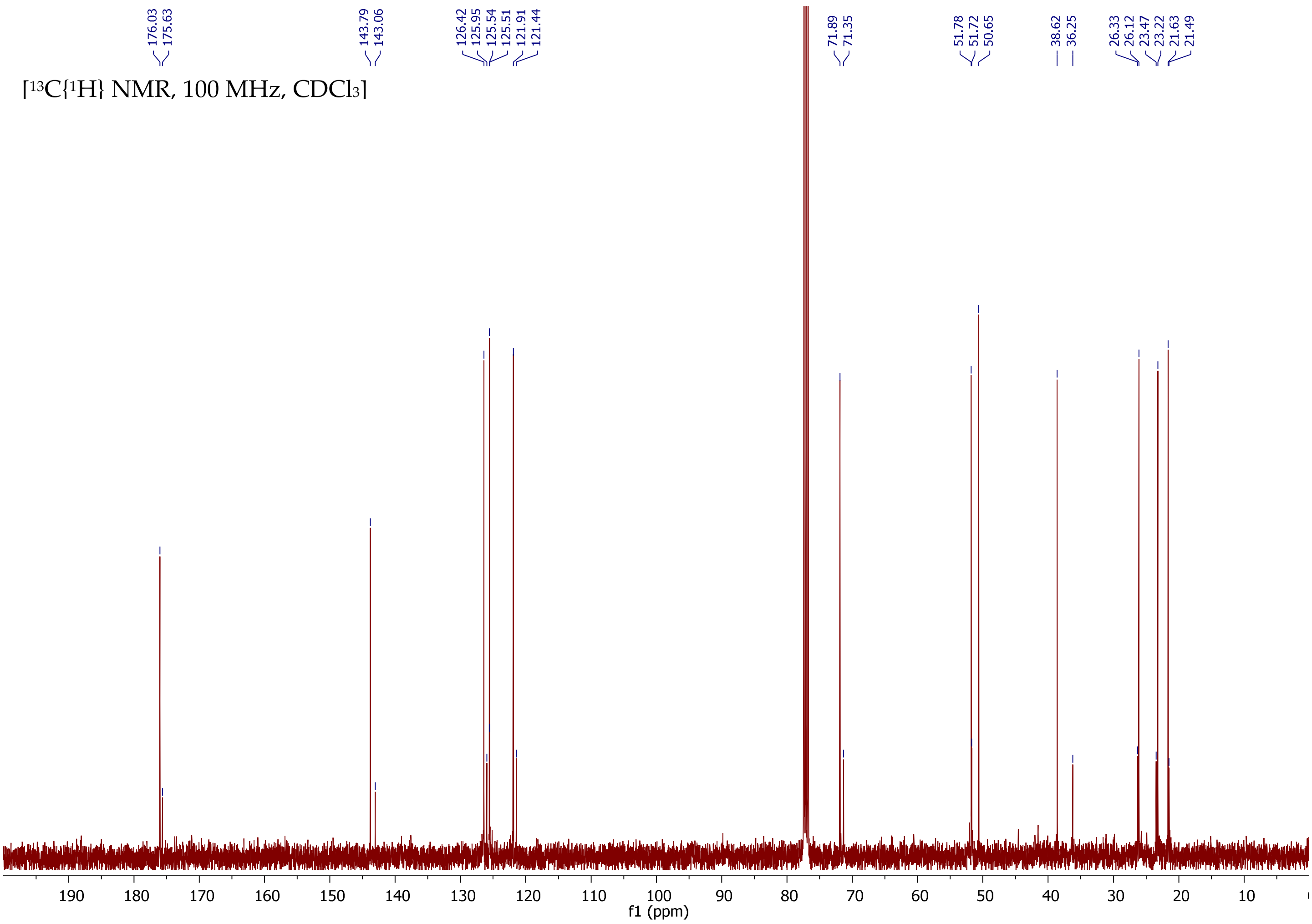

176.03
175.63
143.79
143.06
126.42
125.95
125.54
125.51
121.91
121.44
71.89
71.35
51.78
51.72
50.65
38.62
36.25
26.33
26.12
23.47
23.22
21.63
21.49
[13C{1H} NMR, 100 MHz, CDCl3]
190
180
170
160
150
140
130
120
110
100
90
80
70
60
50
40
30
20
10
f1 (ppm)

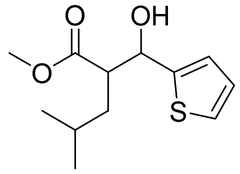

4cag
d.r. = 81:19

[¹H NMR, 400 MHz, CDCl₃]

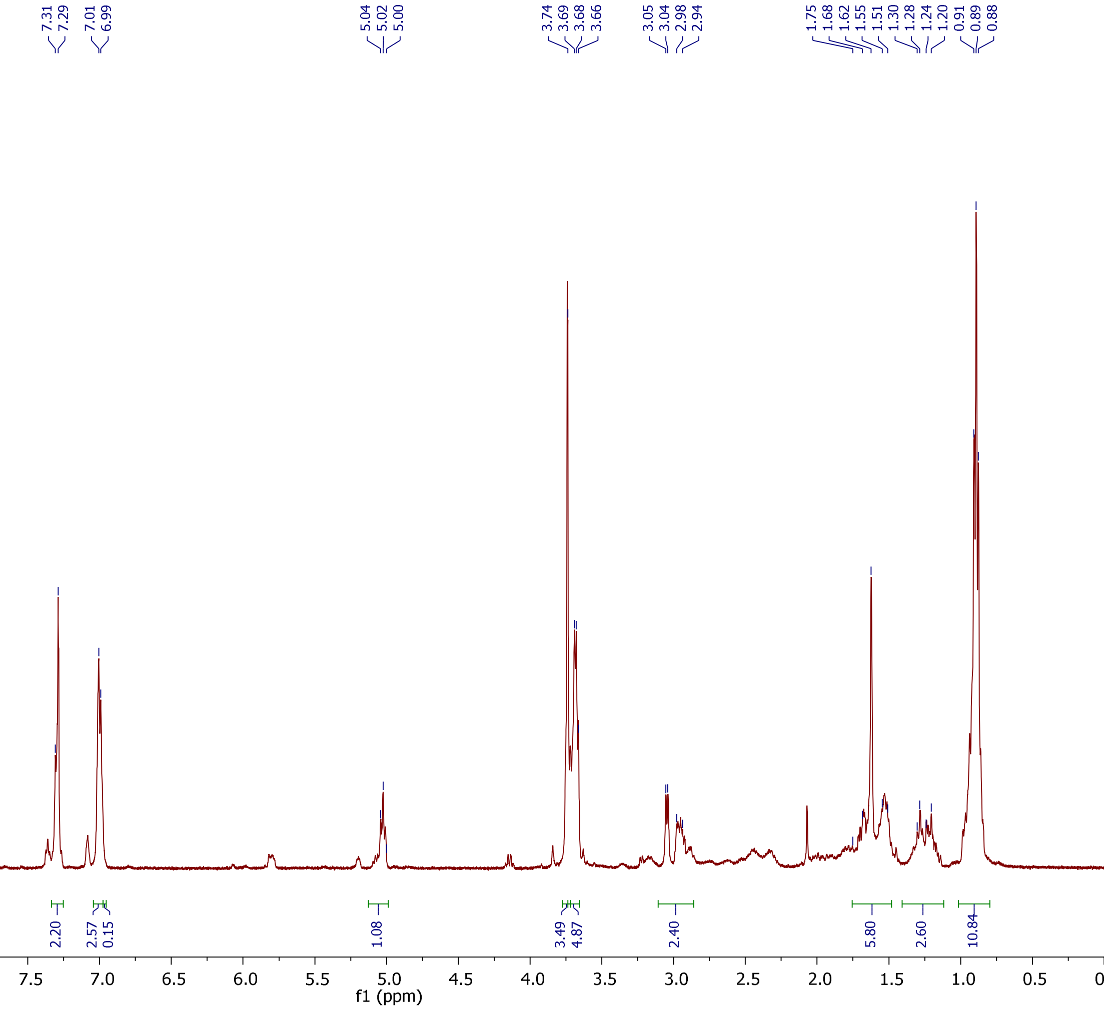

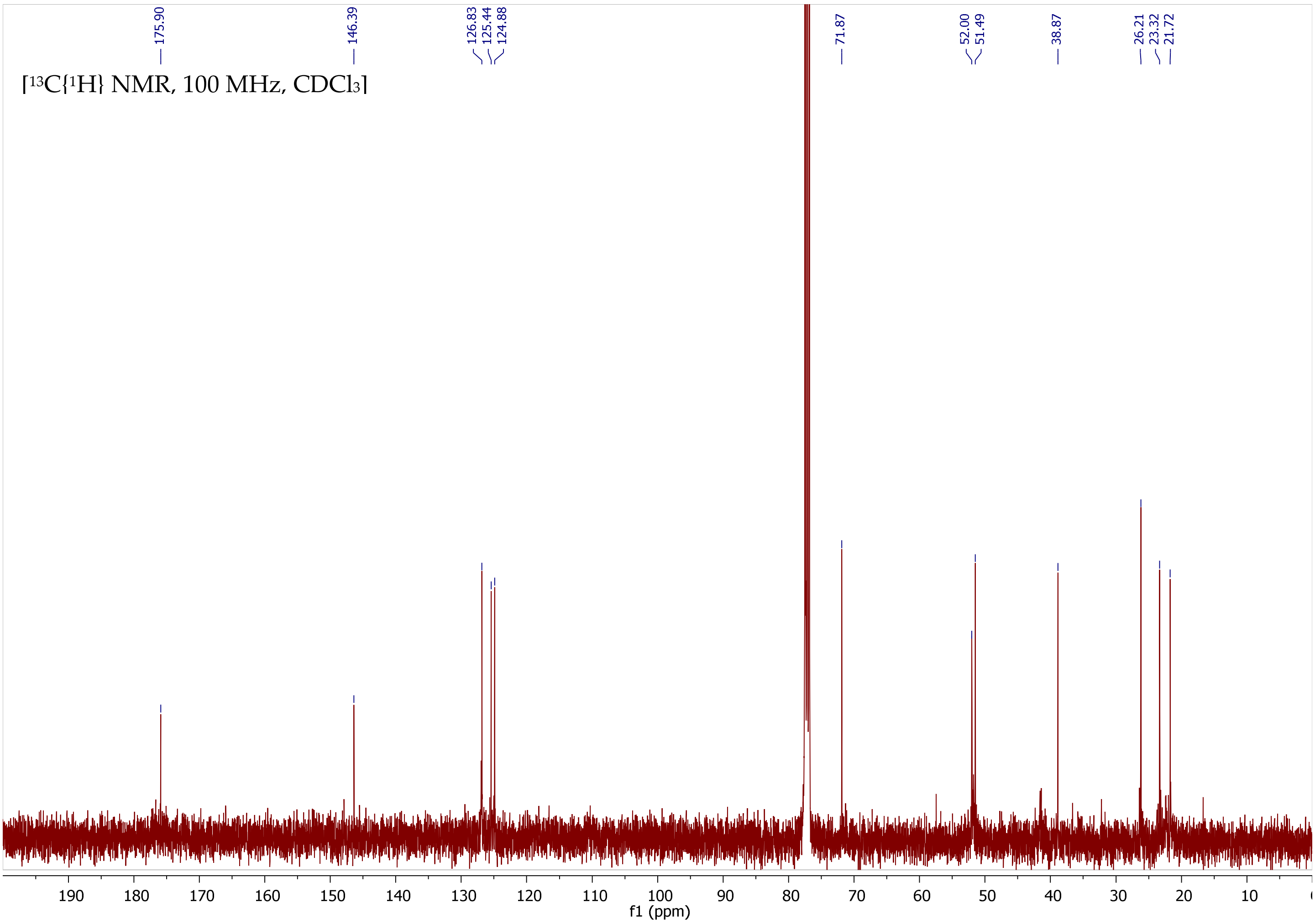

[13C{1H} NMR, 100 MHz, CDCl3]
175.90
146.39
126.83
125.44
124.88
71.87
52.00
51.49
38.87
26.21
23.32
21.72
190
180
170
160
150
140
130
120
110
100
90
80
70
60
50
40
30
20
10
f1 (ppm)

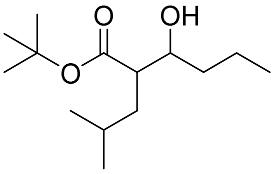

4ceh

d.r. = 51:49

[1H NMR, 400 MHz, $CDCl_3$]

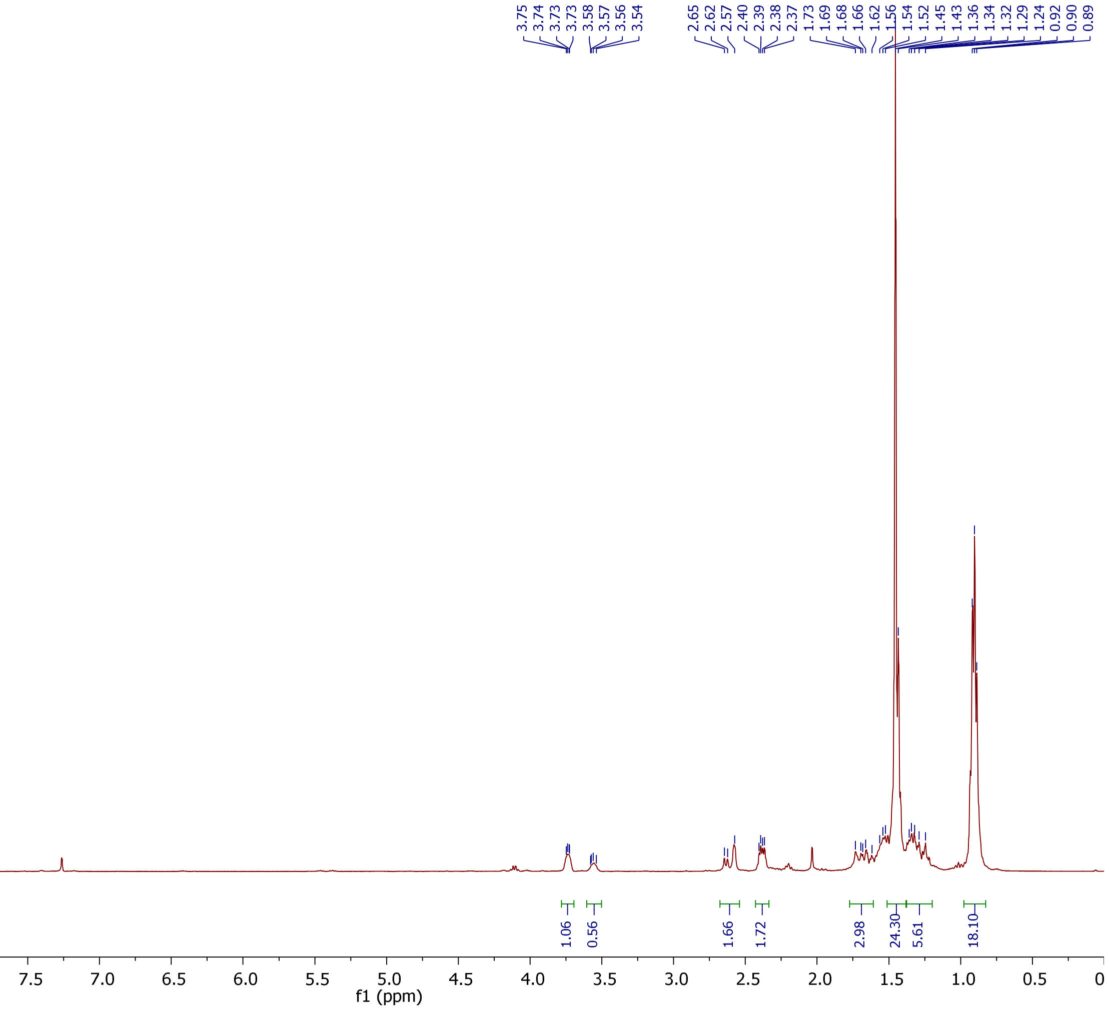

[13C{1H} NMR, 100 MHz, CDCl3]

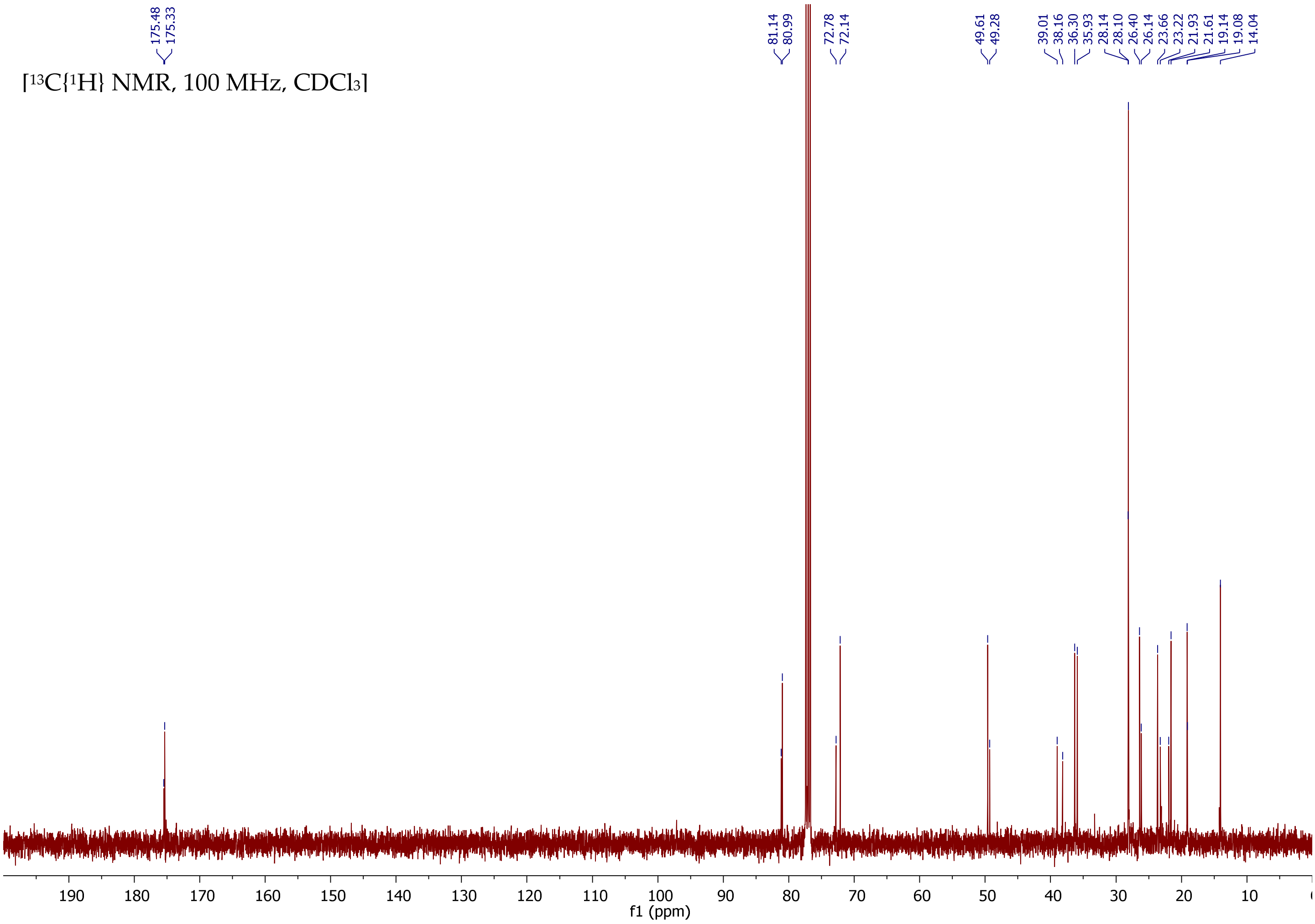

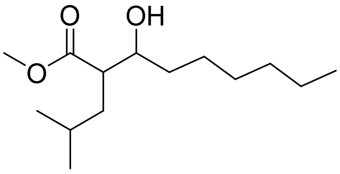

4cai
d.r. = 69:31

[^{1}H NMR, 400 MHz, $CDCl_3$]

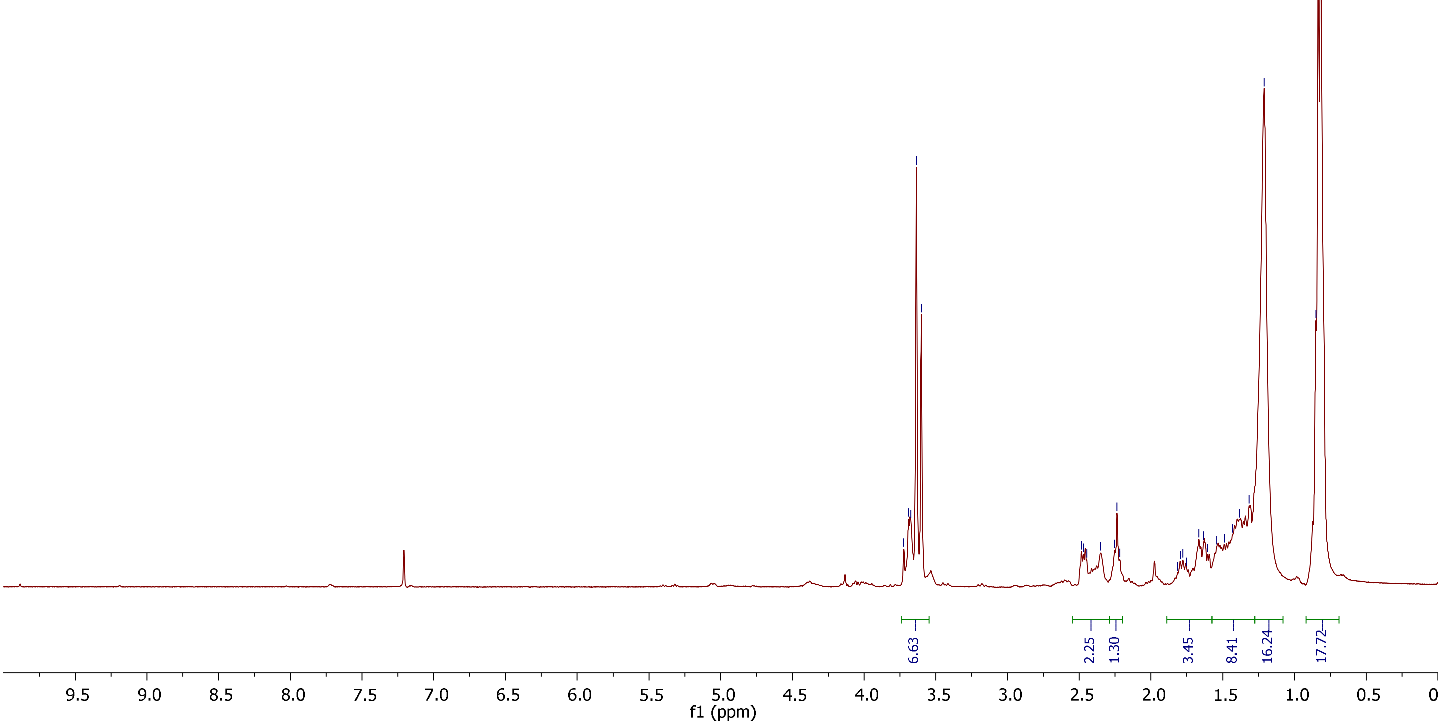

[$^{13}C\{^{1}H\}$ NMR, 100 MHz, $CDCl_3$]

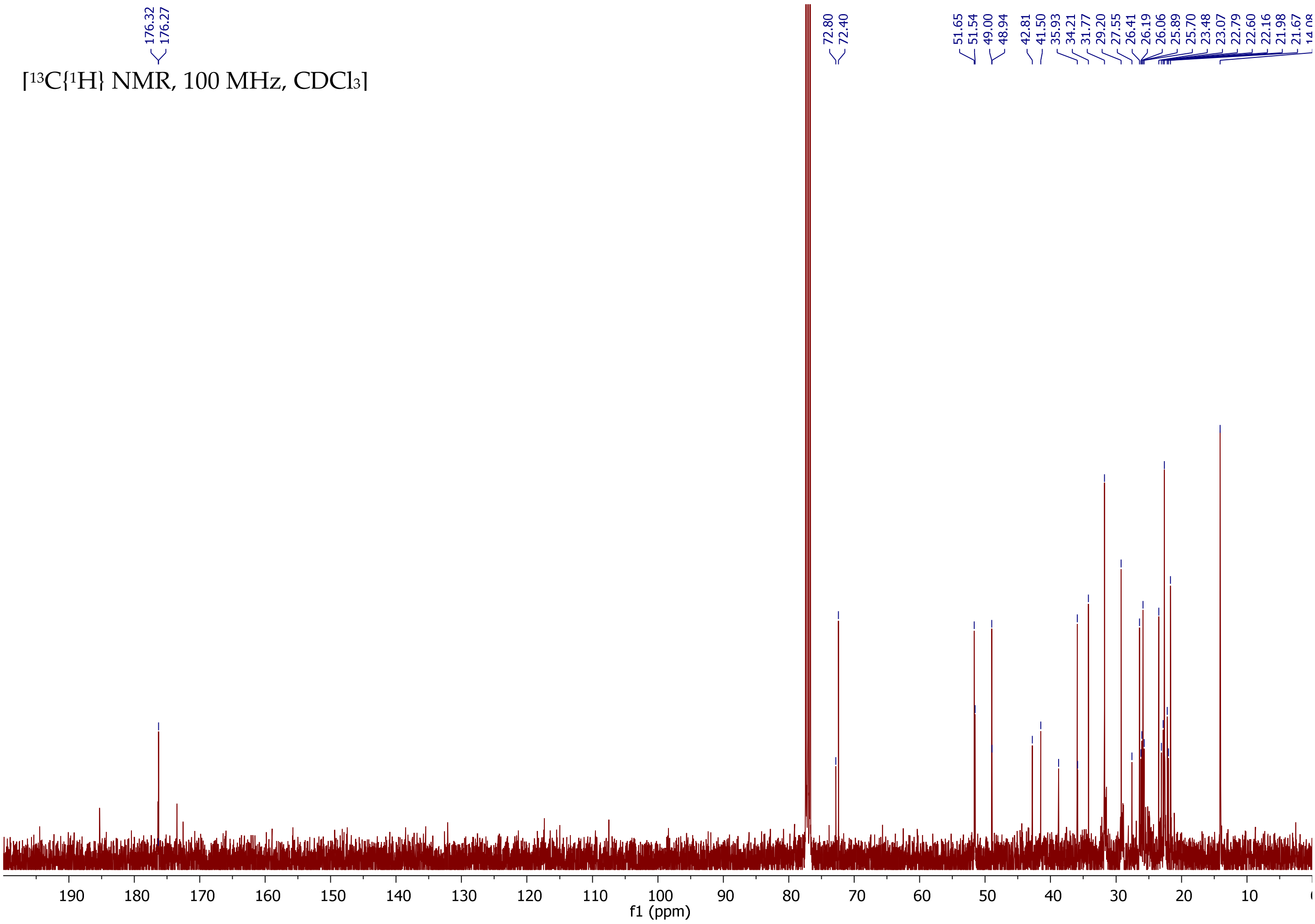

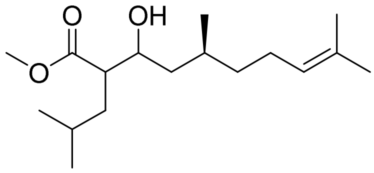

4caj
d.r. = 27:27:27:19

[^{1}H NMR, 400 MHz, $CDCl_3$]

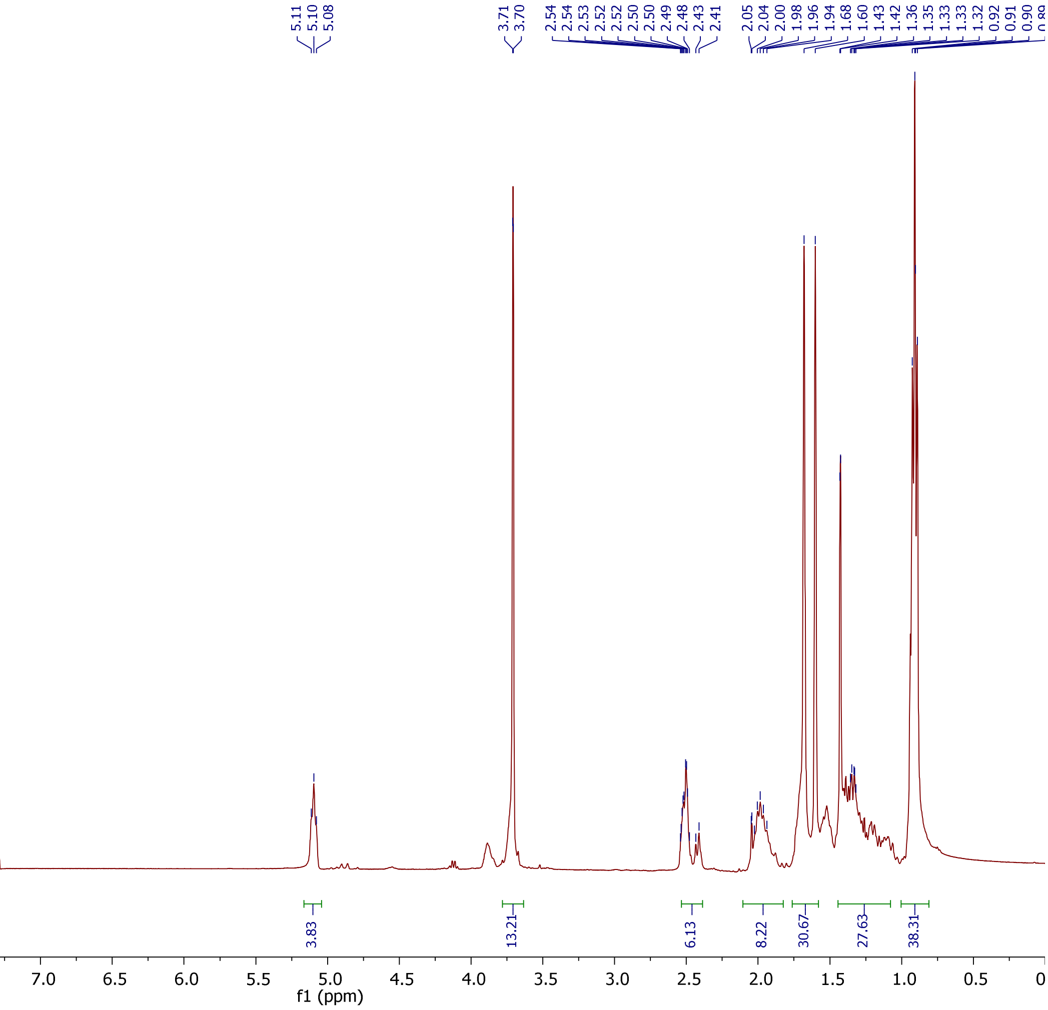

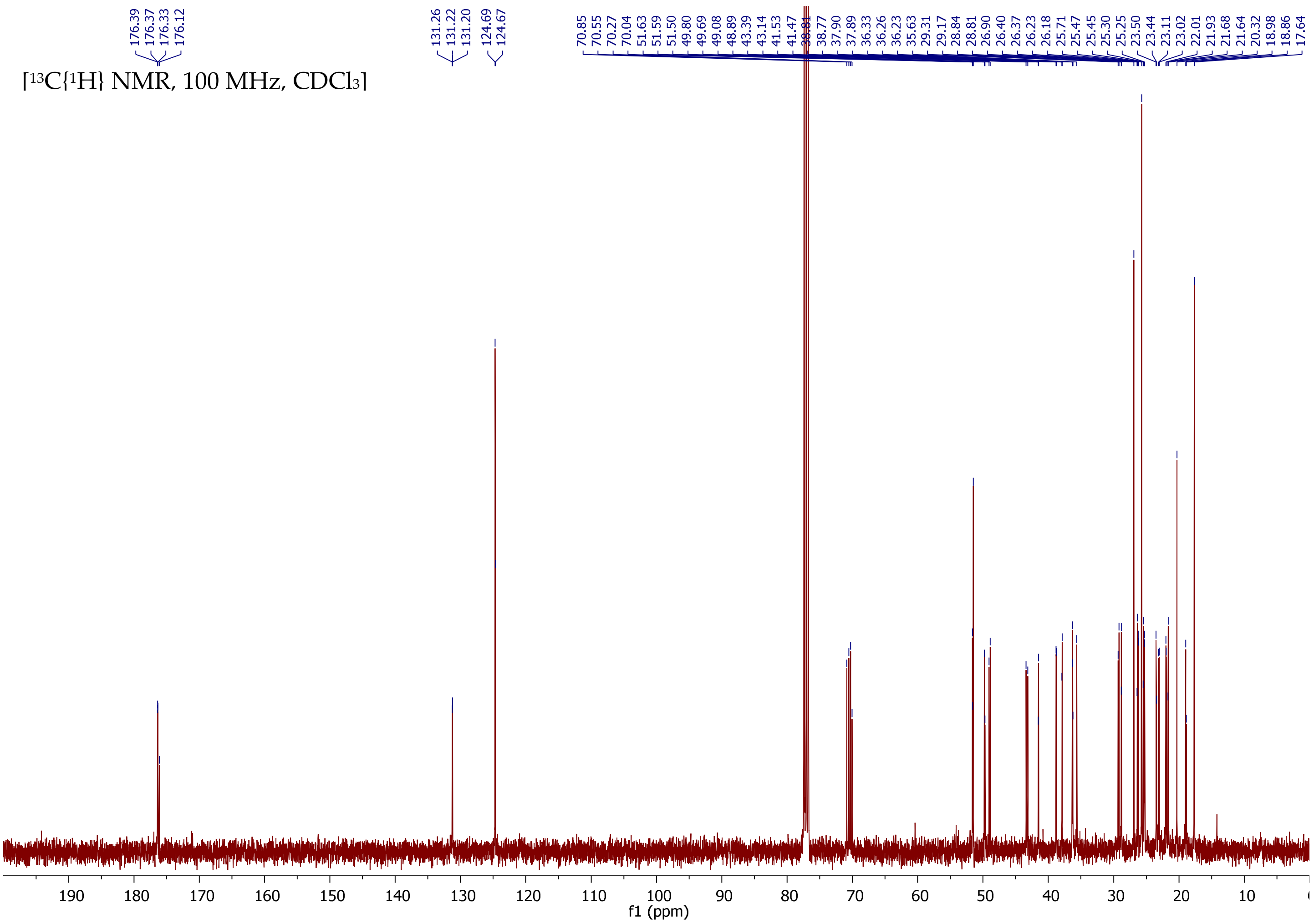
[13C{1H} NMR, 100 MHz, CDCl3]
f1 (ppm)

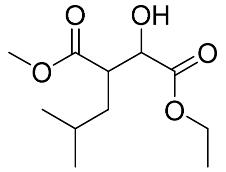

4cak
d.r. = 60:40

[^{1}H NMR, 400 MHz, $CDCl_3$]

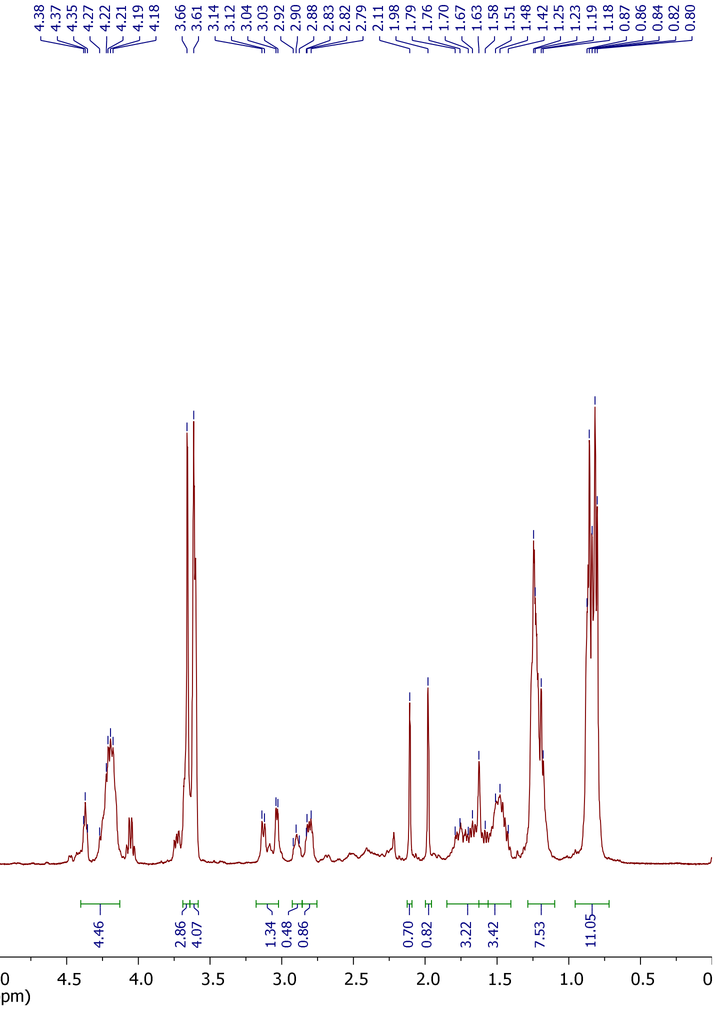

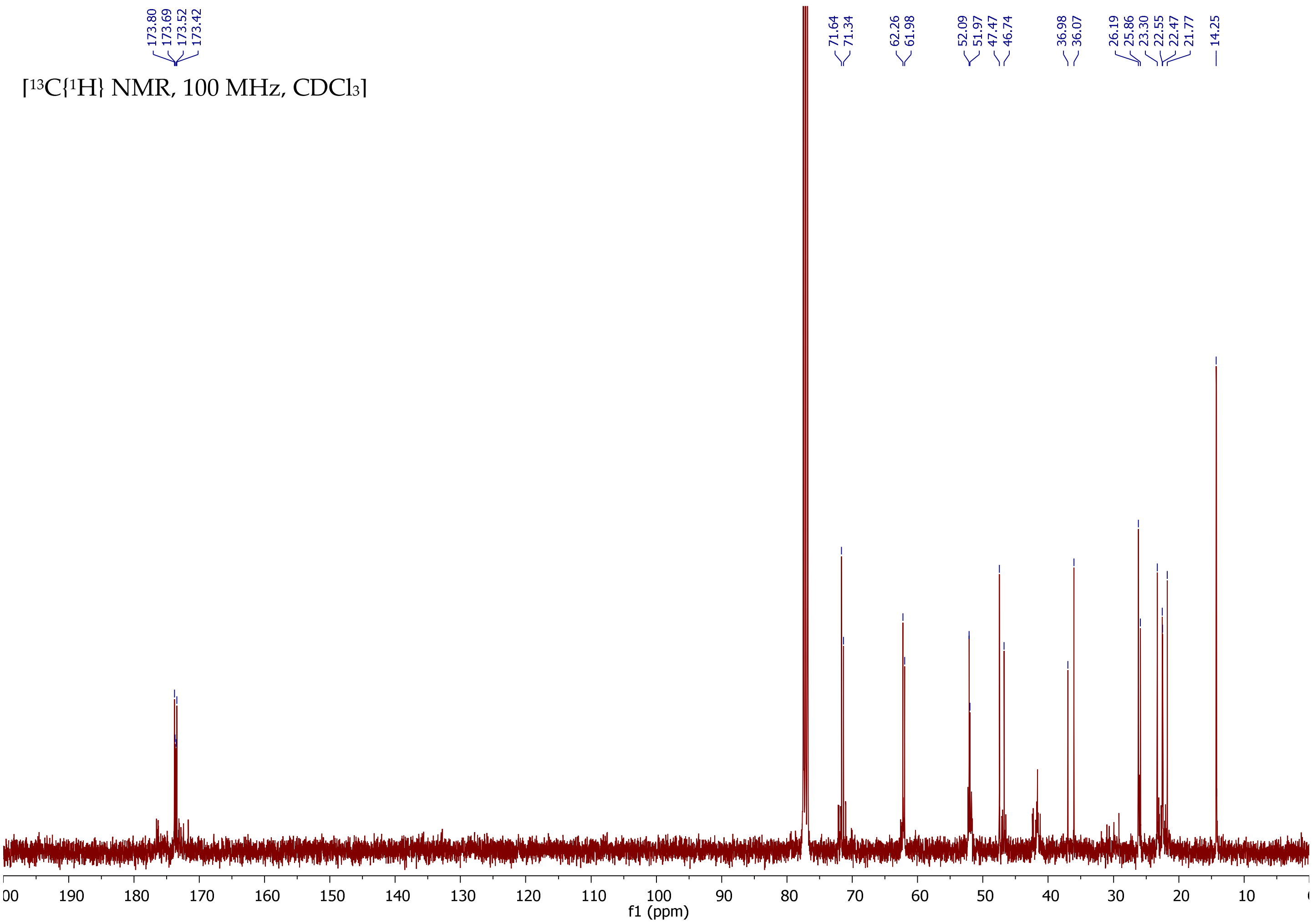
[13C{1H} NMR, 100 MHz, CDCl3]
173.80
173.69
173.52
173.42
71.64
71.34
62.26
61.98
52.09
51.97
47.47
46.74
36.98
36.07
26.19
25.86
23.30
22.55
22.47
21.77
14.25
f1 (ppm)

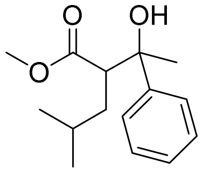

6caa
d.r. = 61:39
[¹H NMR, 400 MHz, CDCl₃]

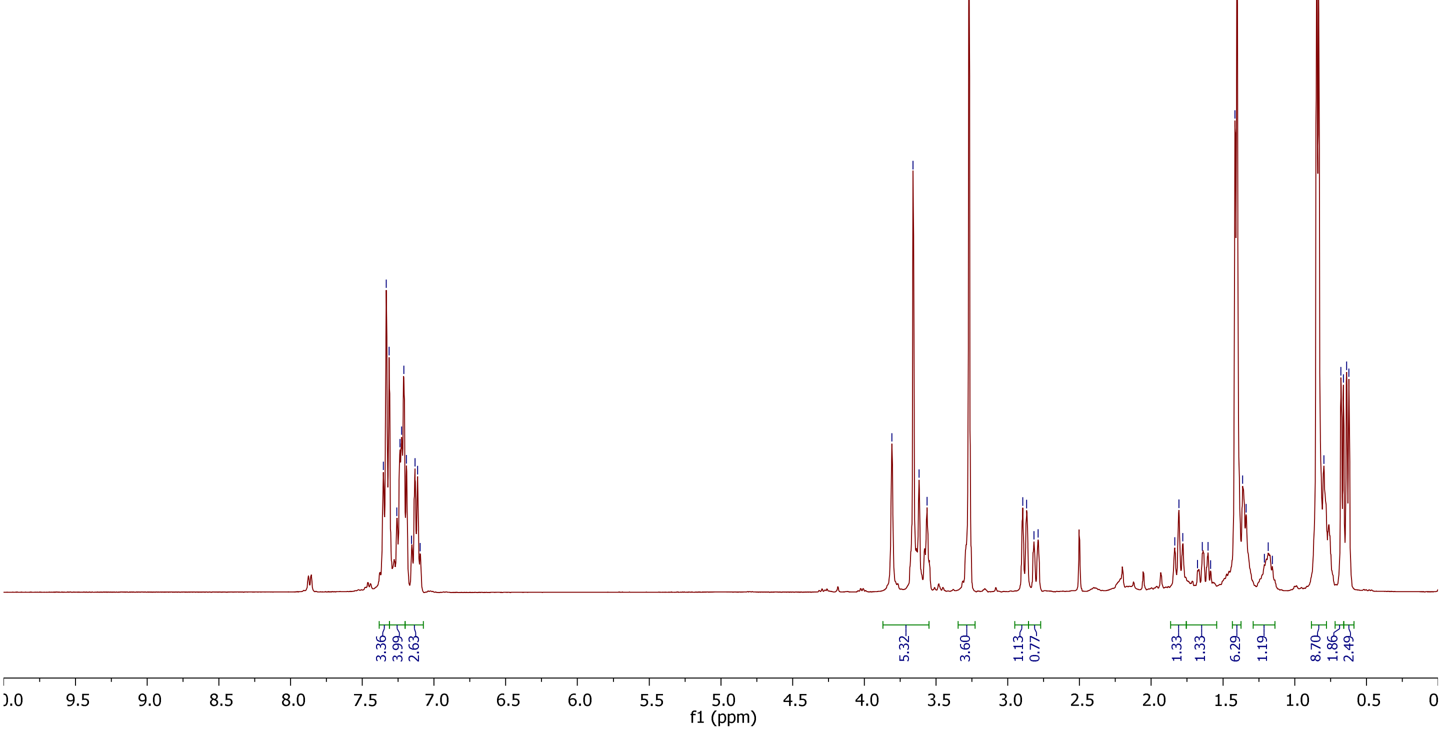

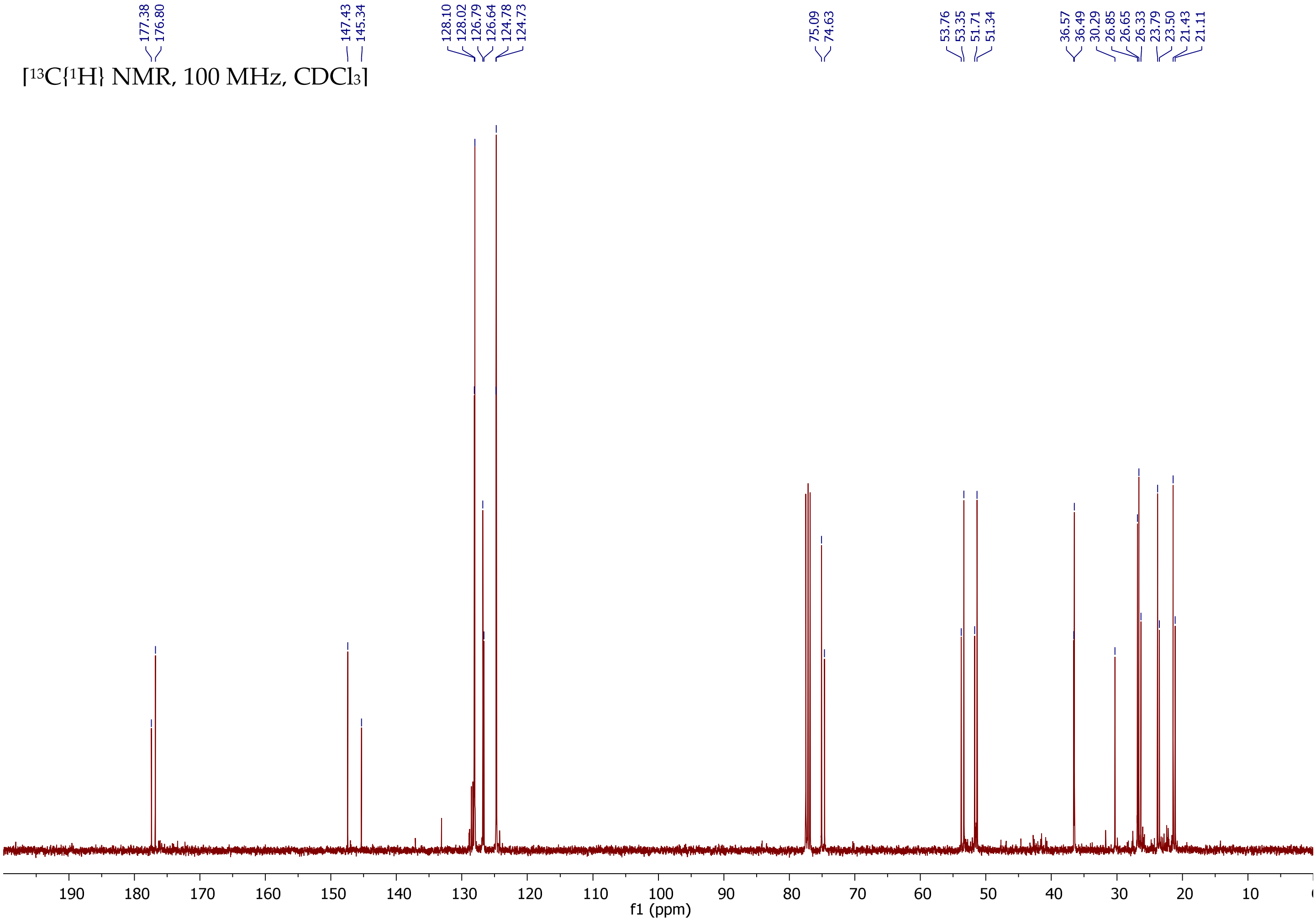
177.38
176.80
147.43
145.34
128.10
128.02
126.79
126.64
124.78
124.73
75.09
74.63
53.76
53.35
51.71
51.34
36.57
36.49
30.29
26.85
26.65
26.33
23.79
23.50
21.43
21.11
[13C{1H} NMR, 100 MHz, CDCl3]
190
180
170
160
150
140
130
120
110
100
90
80
70
60
50
40
30
20
10
f1 (ppm)

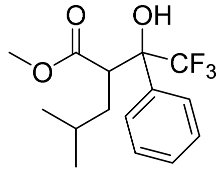

6cab
d.r. = 59:41

[¹H NMR, 400 MHz, CDCl3]

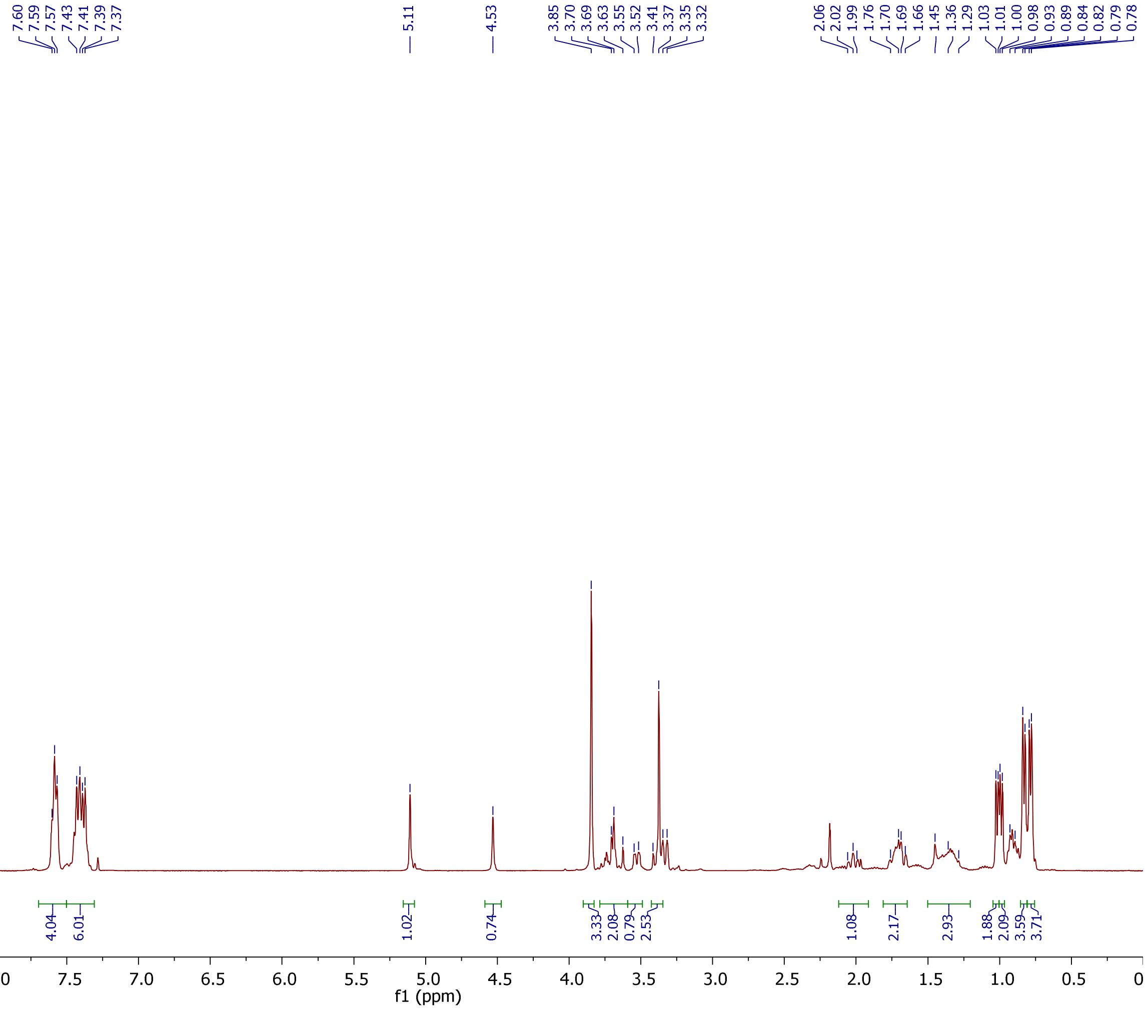

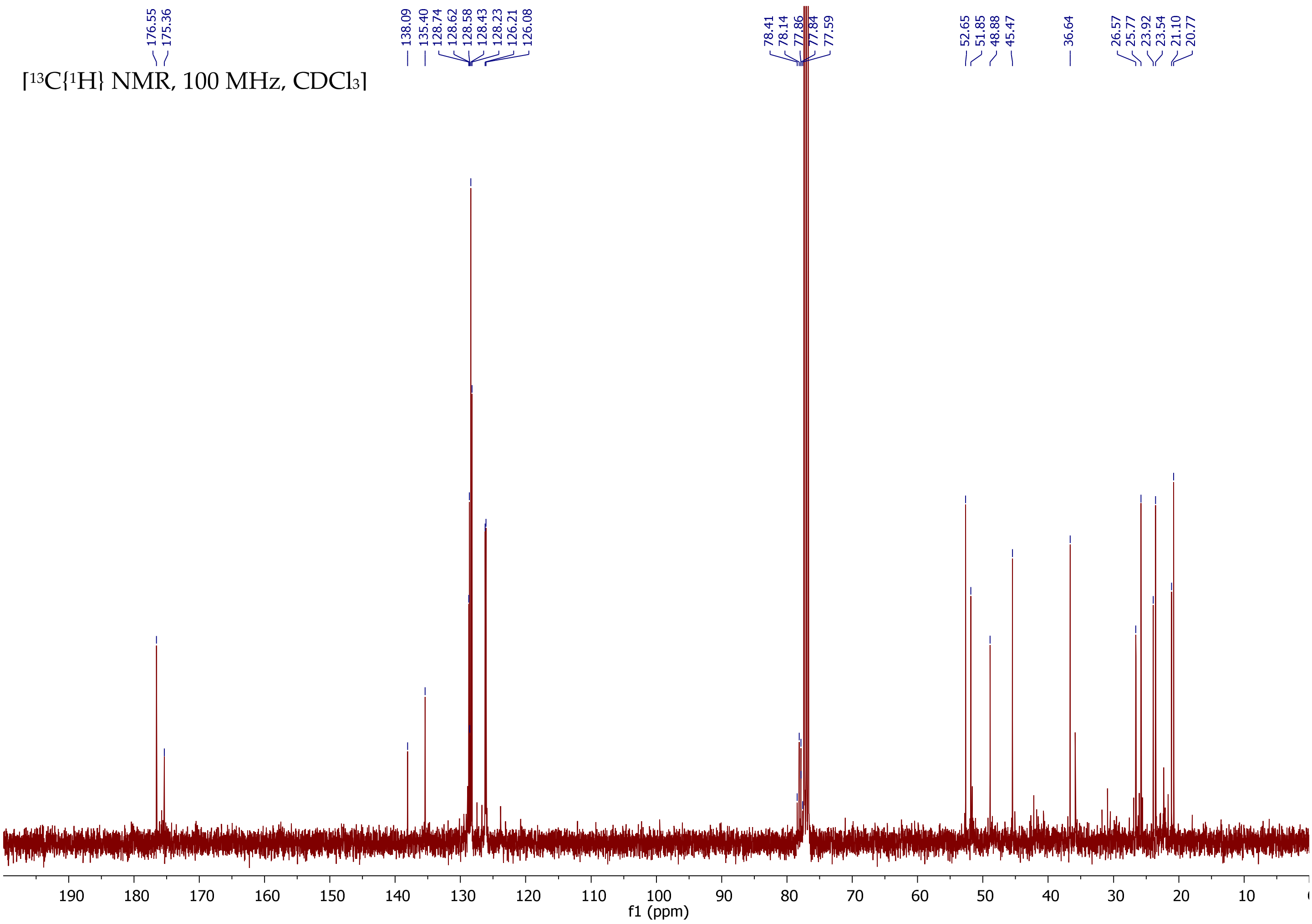
[13C{1H} NMR, 100 MHz, CDCl3]
176.55
175.36
138.09
135.40
128.74
128.62
128.58
128.43
128.23
126.21
126.08
78.41
78.14
77.86
77.84
77.59
52.65
51.85
48.88
45.47
36.64
26.57
25.77
23.92
23.54
21.10
20.77
190
180
170
160
150
140
130
120
110
100
90
80
70
60
50
40
30
20
10
f1 (ppm)

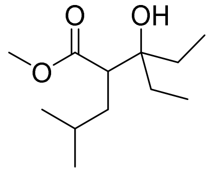

6cac

[¹H NMR, 400 MHz, CDCl₃]

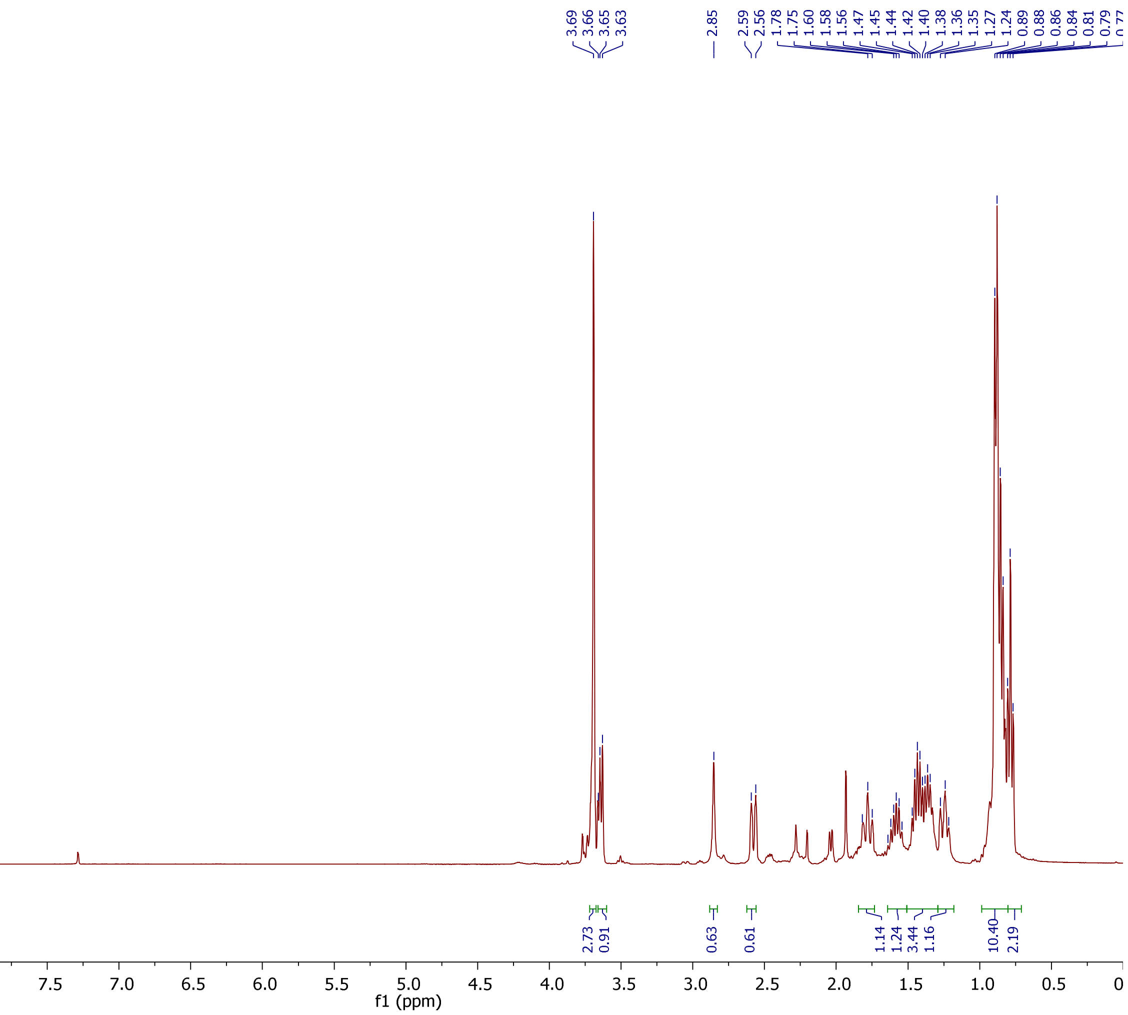

[$^{13}C\{^1H\}$ NMR, 100 MHz, $CDCl_3$]

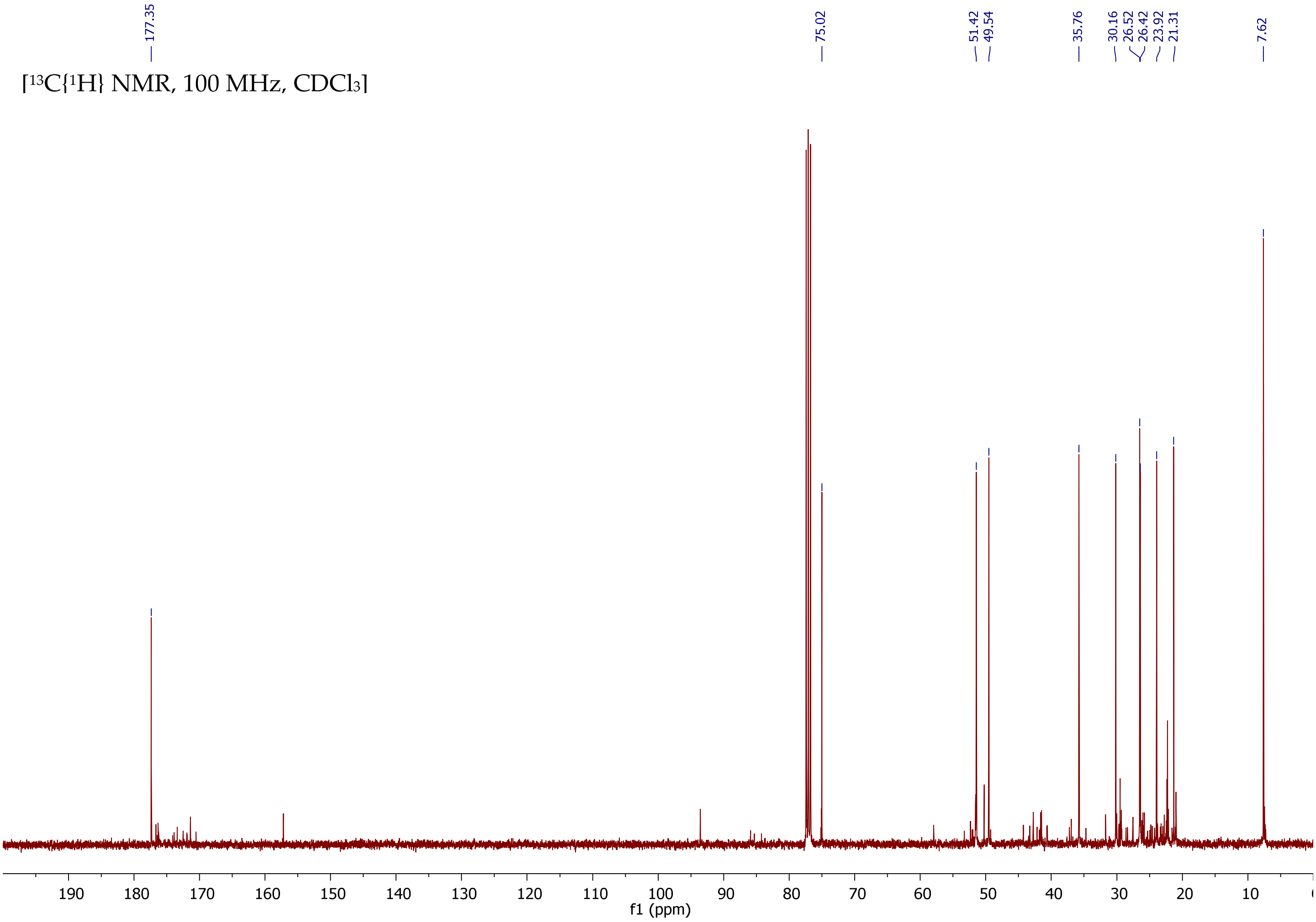

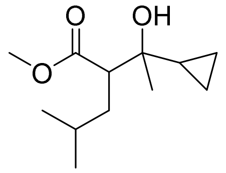

6cad
d.r. = 79:21

[1H NMR, 400 MHz, CDCl3]

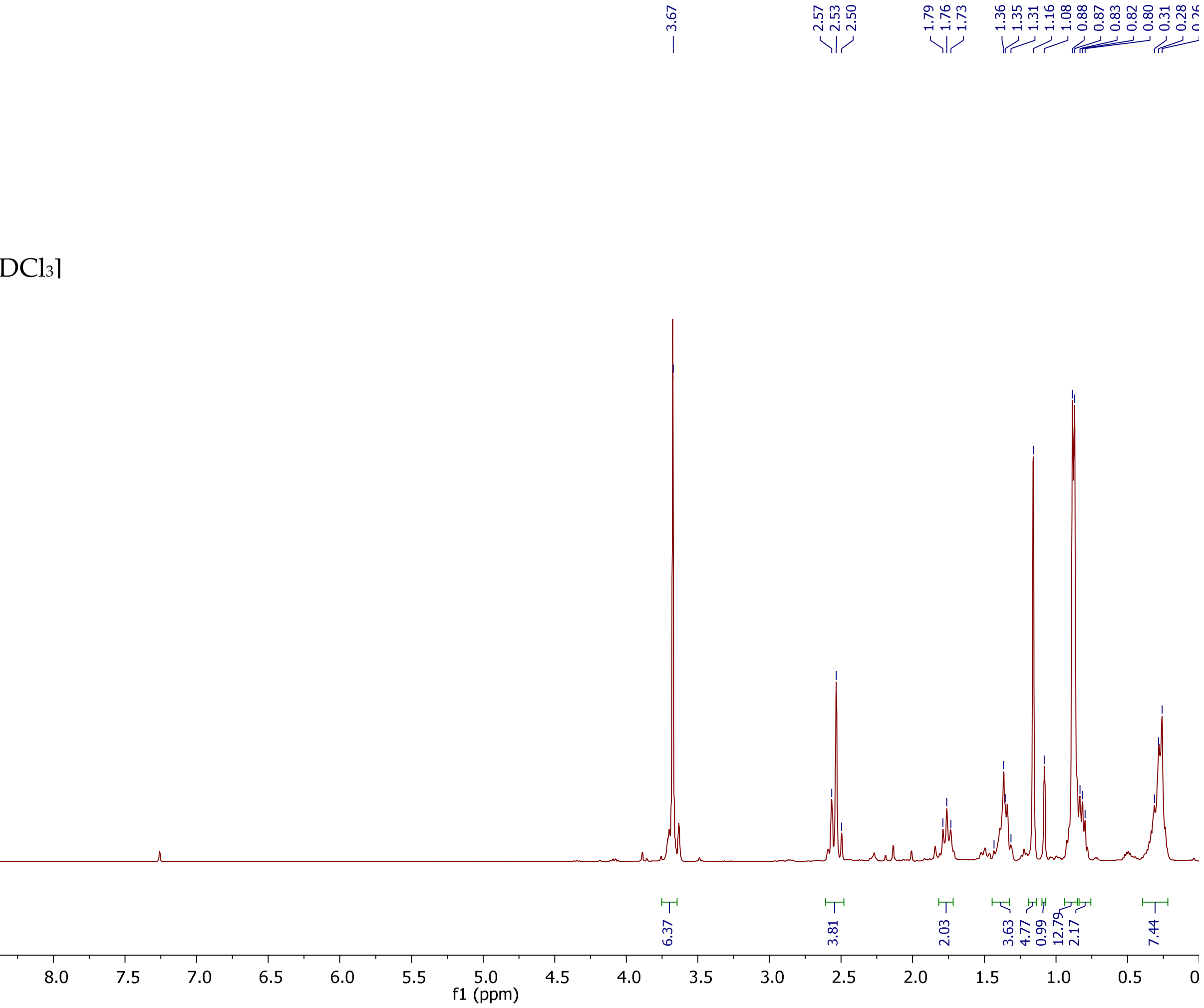

[$^{13}C\{^{1}H\}$ NMR, 100 MHz, $CDCl_3$]

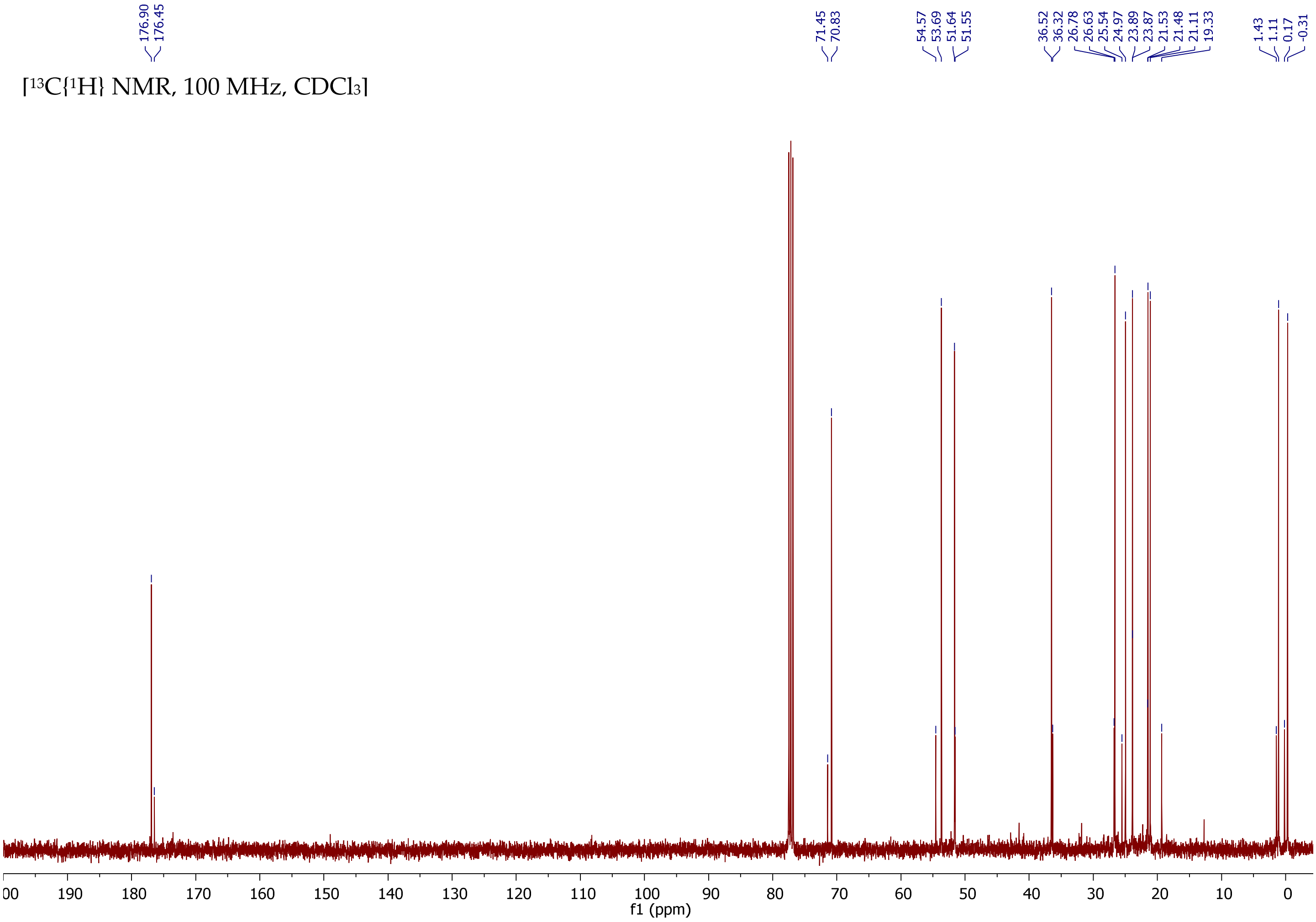

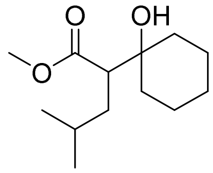

6cae

[^{1}H NMR, 400 MHz, $CDCl_3$]

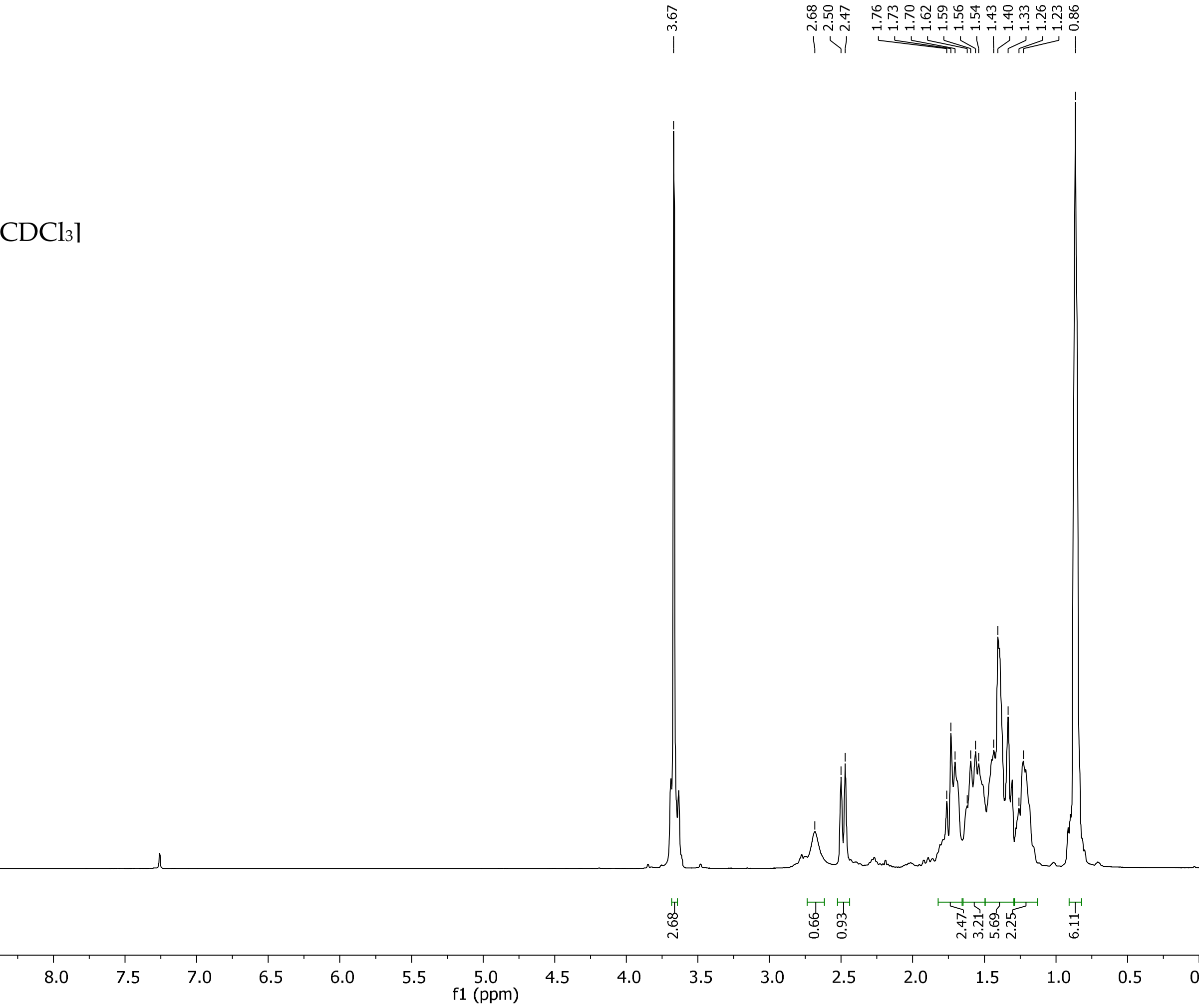

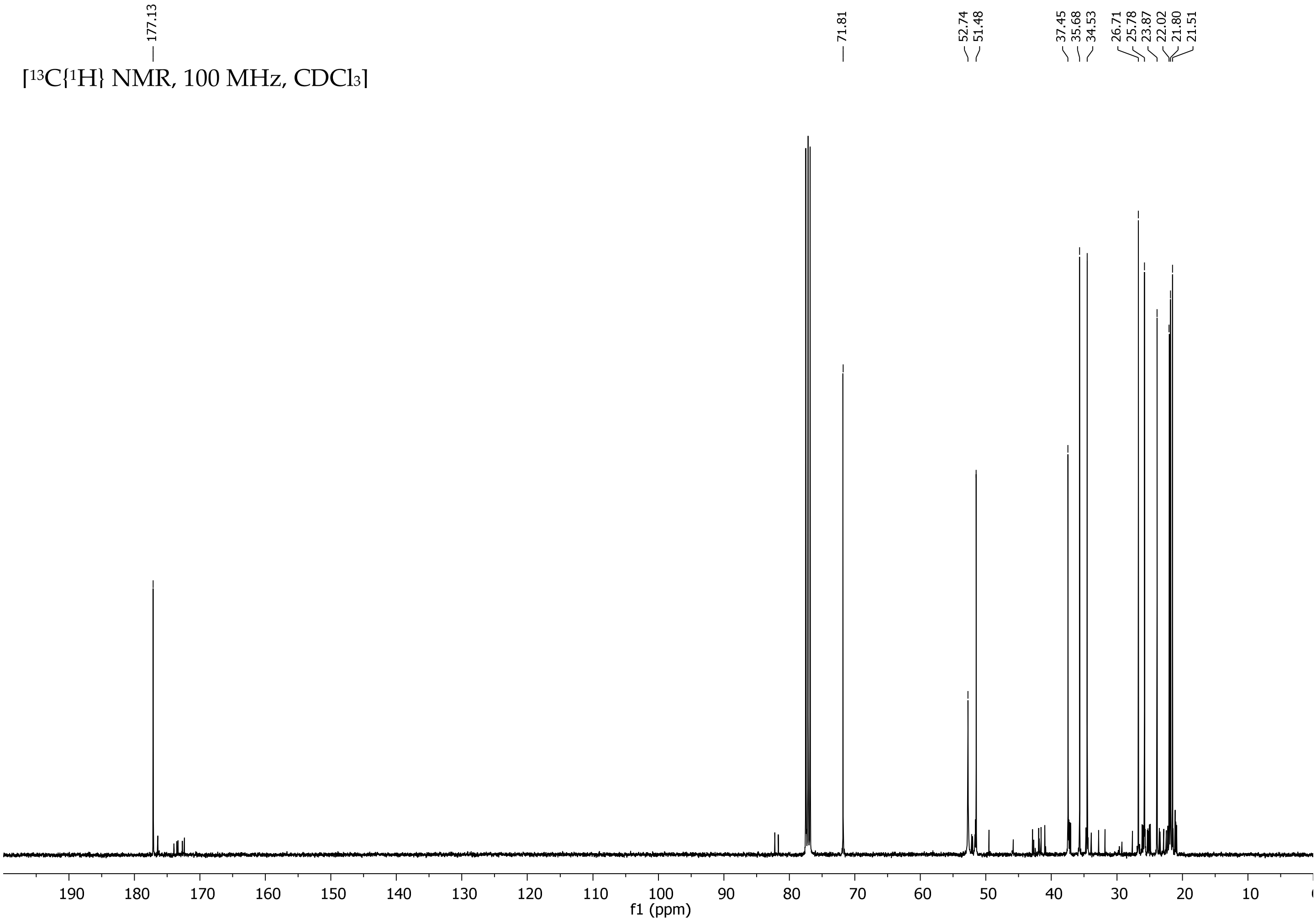
177.13
[13C{1H} NMR, 100 MHz, CDCl3]
71.81
52.74
51.48
37.45
35.68
34.53
26.71
25.78
23.87
22.02
21.80
21.51
190
180
170
160
150
140
130
120
110
100
90
80
70
60
50
40
30
20
10
f1 (ppm)

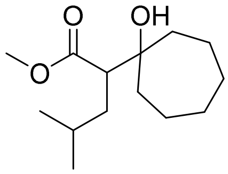

6caf

[^{1}H NMR, 400 MHz, $CDCl_3$]

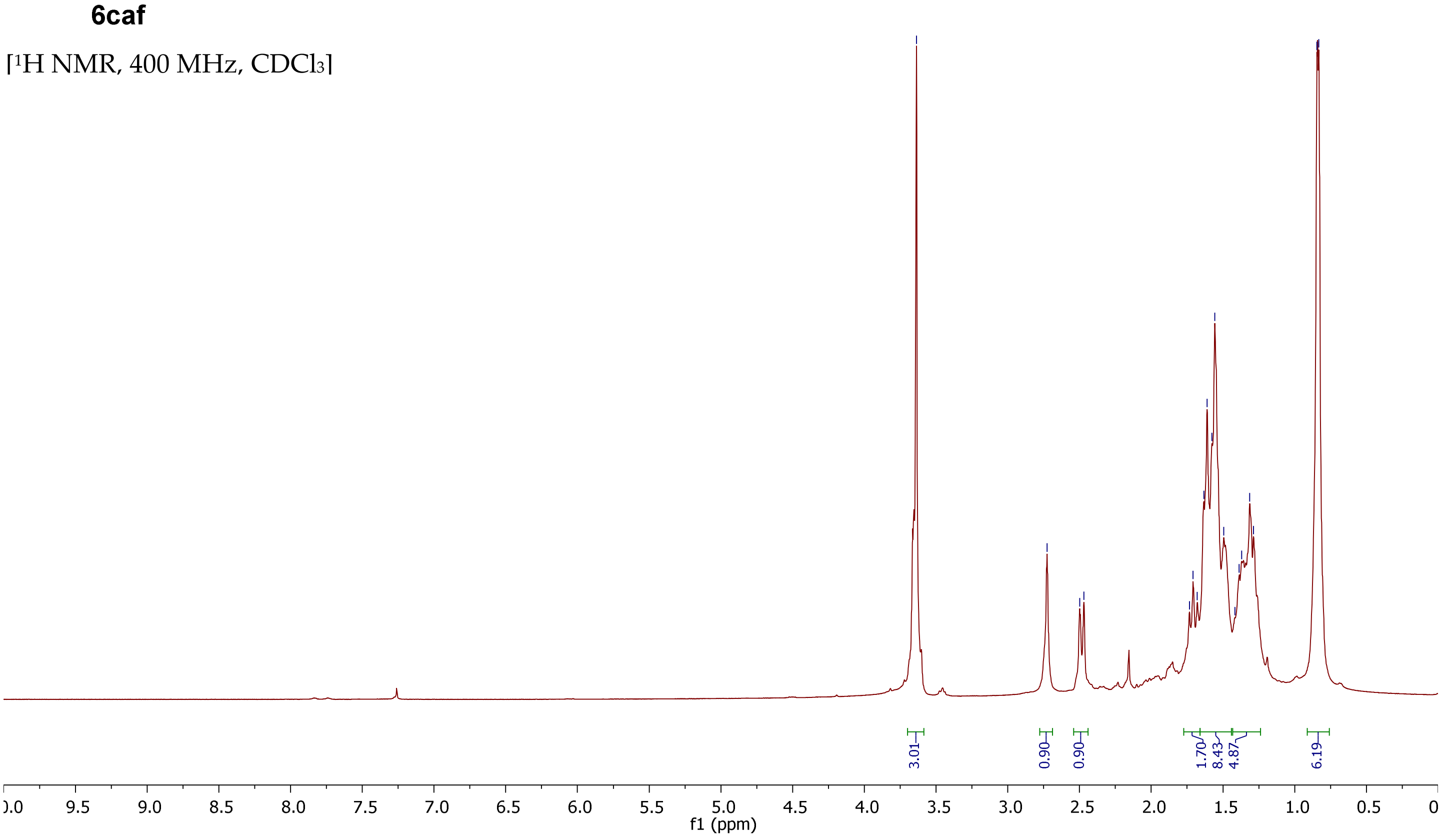

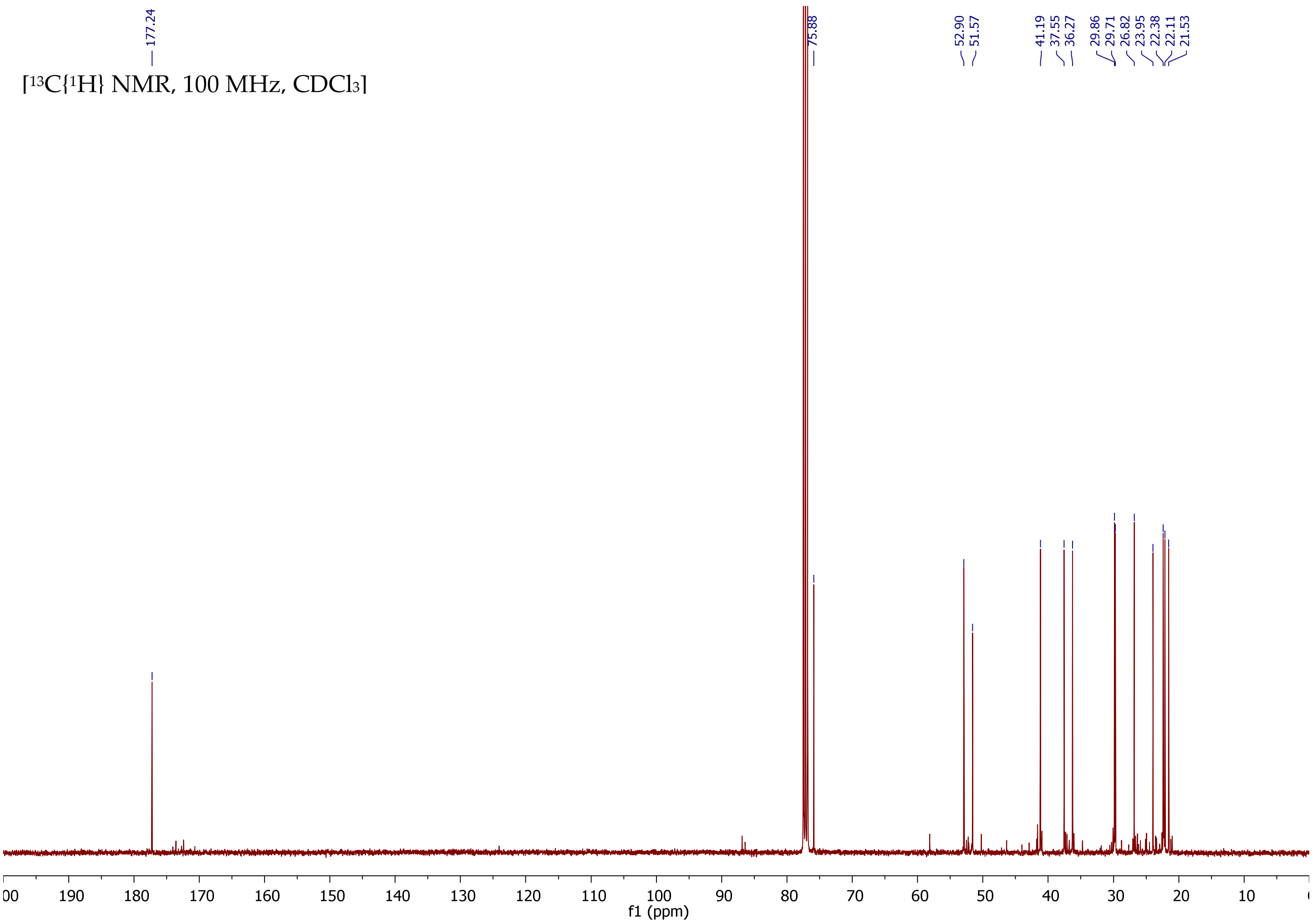
177.24
75.88
52.90
51.57
41.19
37.55
36.27
29.86
29.71
26.82
23.95
22.38
22.11
21.53
[13C{1H} NMR, 100 MHz, CDCl3]
f1 (ppm)

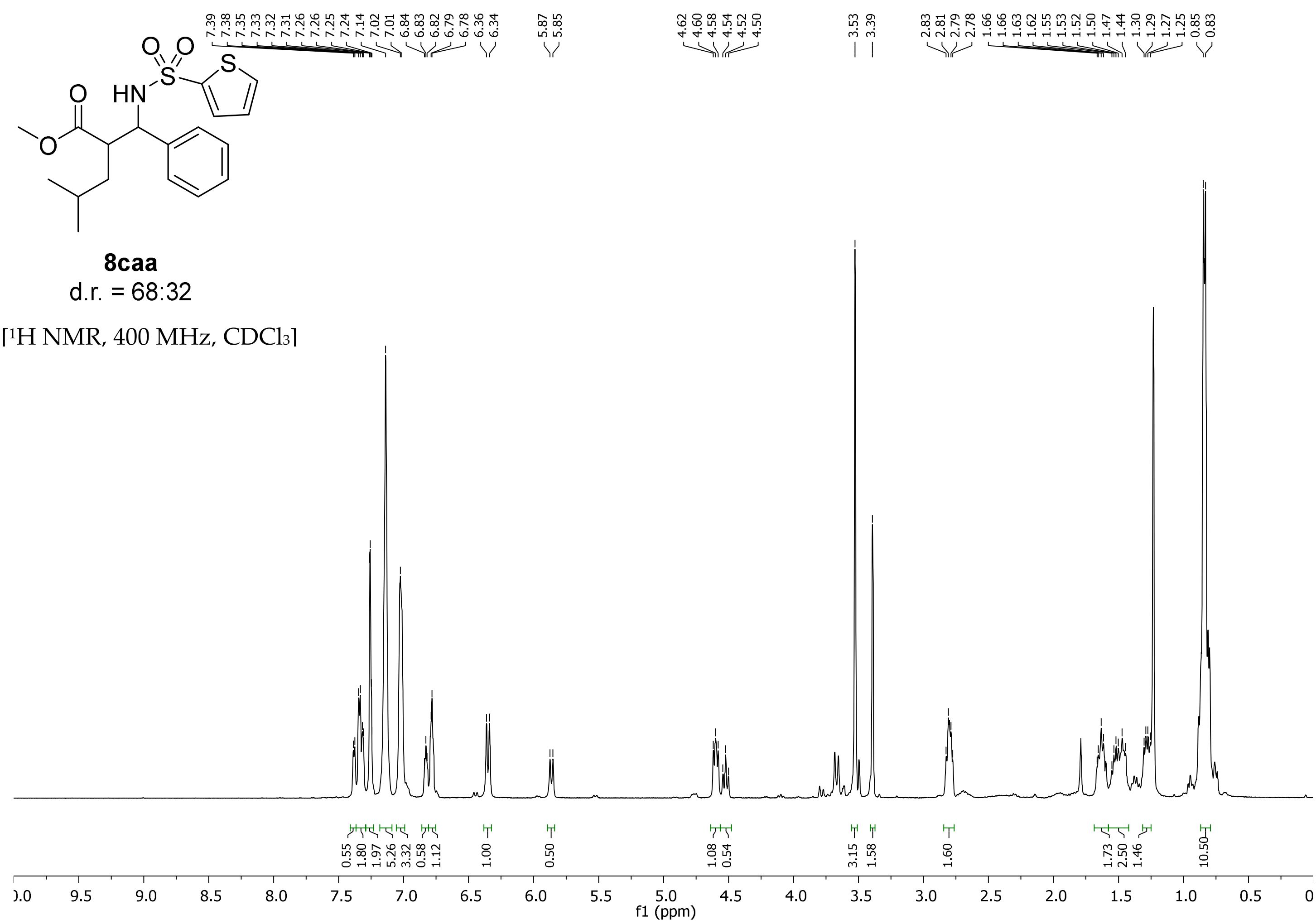
8caa
d.r. = 68:32
[1H NMR, 400 MHz, CDCl3]
f1 (ppm)

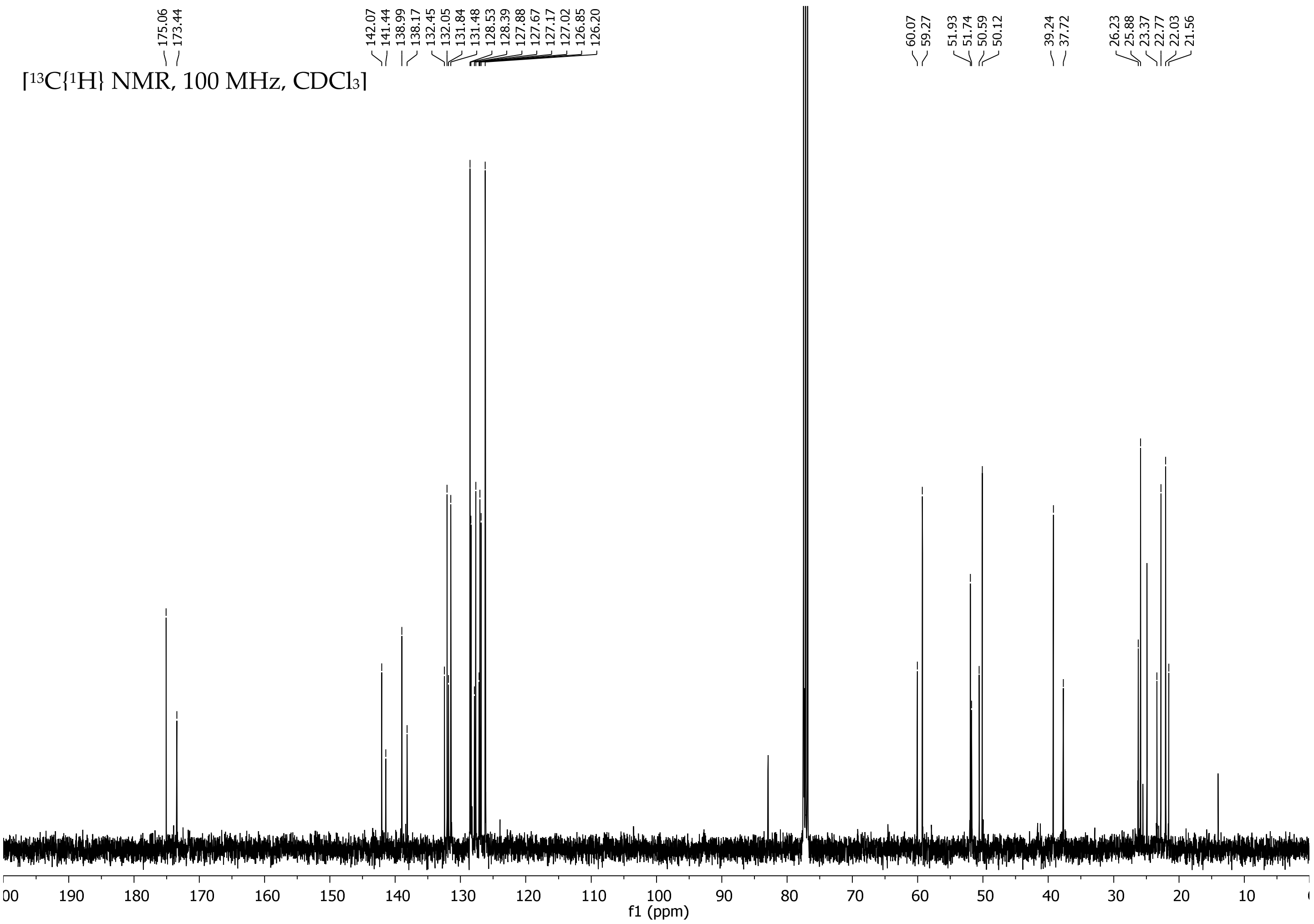
[13C{1H} NMR, 100 MHz, CDCl3]
175.06
173.44
142.07
141.44
138.99
138.17
132.45
132.05
131.84
131.48
128.53
128.39
127.88
127.67
127.17
127.02
126.85
126.20
60.07
59.27
51.93
51.74
50.59
50.12
39.24
37.72
26.23
25.88
23.37
22.77
22.03
21.56
190
180
170
160
150
140
130
120
110
100
90
80
70
60
50
40
30
20
10
f1 (ppm)

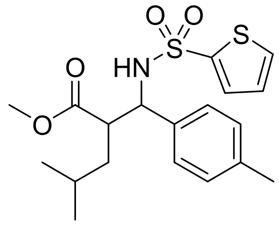

8cab
d.r. = 58:42

[^{1}H NMR, 400 MHz, $CDCl_3$]

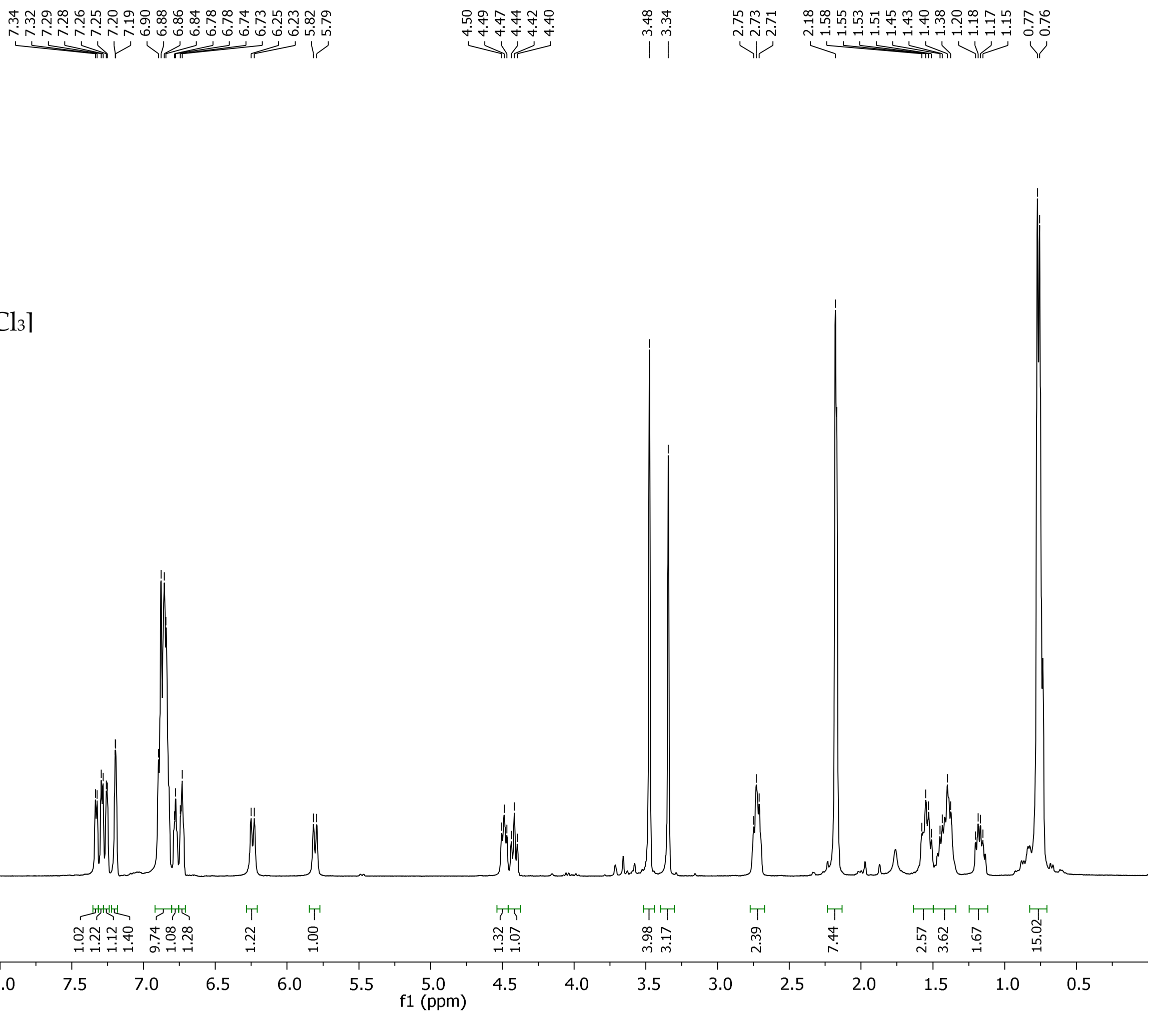

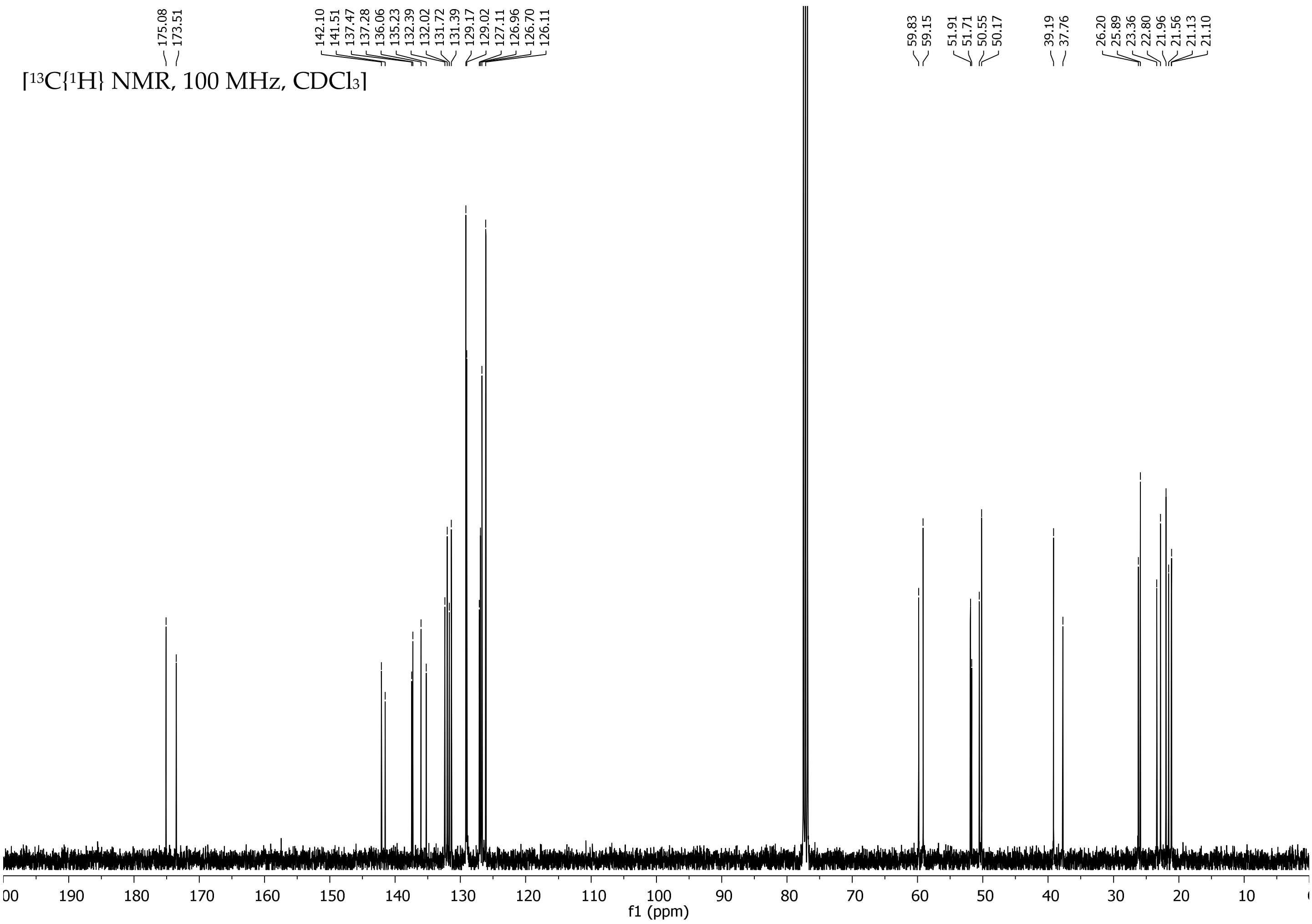
[13C{1H} NMR, 100 MHz, CDCl3]
175.08
173.51
142.10
141.51
137.47
137.28
136.06
135.23
132.39
132.02
131.72
131.39
129.17
129.02
127.11
126.96
126.70
126.11
59.83
59.15
51.91
51.71
50.55
50.17
39.19
37.76
26.20
25.89
23.36
22.80
21.96
21.56
21.13
21.10
f1 (ppm)

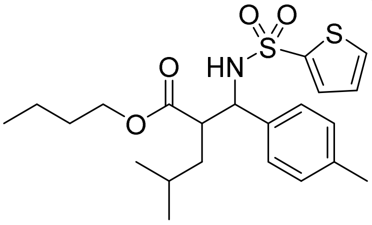

8ccb
d.r. = 53:47
[1H NMR, 400 MHz, $CDCl_3$]

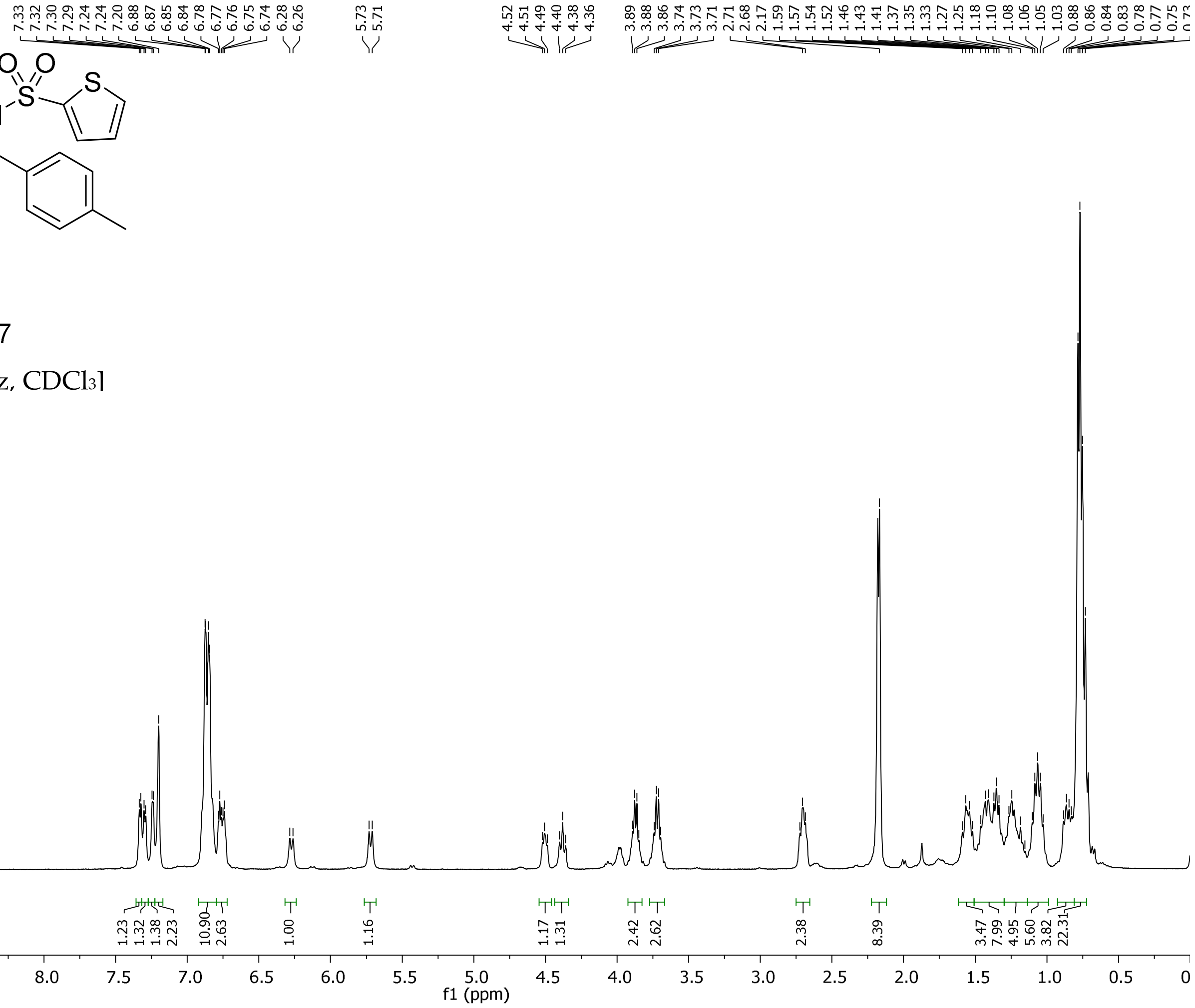

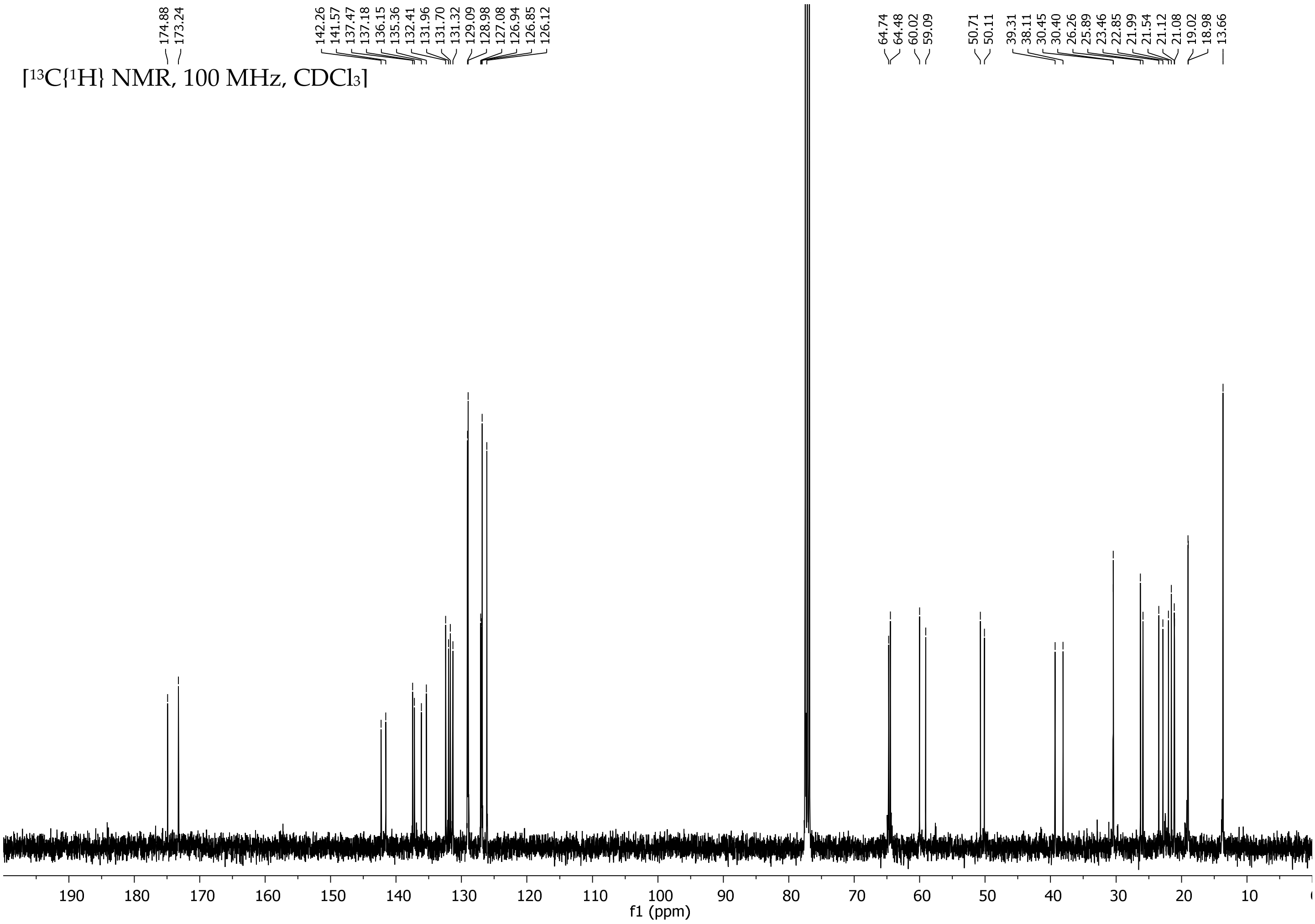
[$^{13}C\{^1H\}$ NMR, 100 MHz, $CDCl_3$]
174.88
173.24
142.26
141.57
137.47
137.18
136.15
135.36
132.41
131.96
131.70
131.32
129.09
128.98
127.08
126.94
126.85
126.12
64.74
64.48
60.02
59.09
50.71
50.11
39.31
38.11
30.45
30.40
26.26
25.89
23.46
22.85
21.99
21.54
21.12
21.08
19.02
18.98
13.66
f1 (ppm)

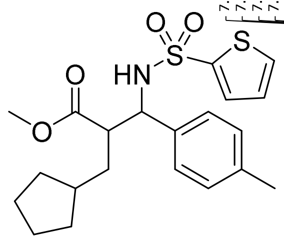

8dab
d.r. = 52:48

[^{1}H NMR, 400 MHz, $CDCl_3$]

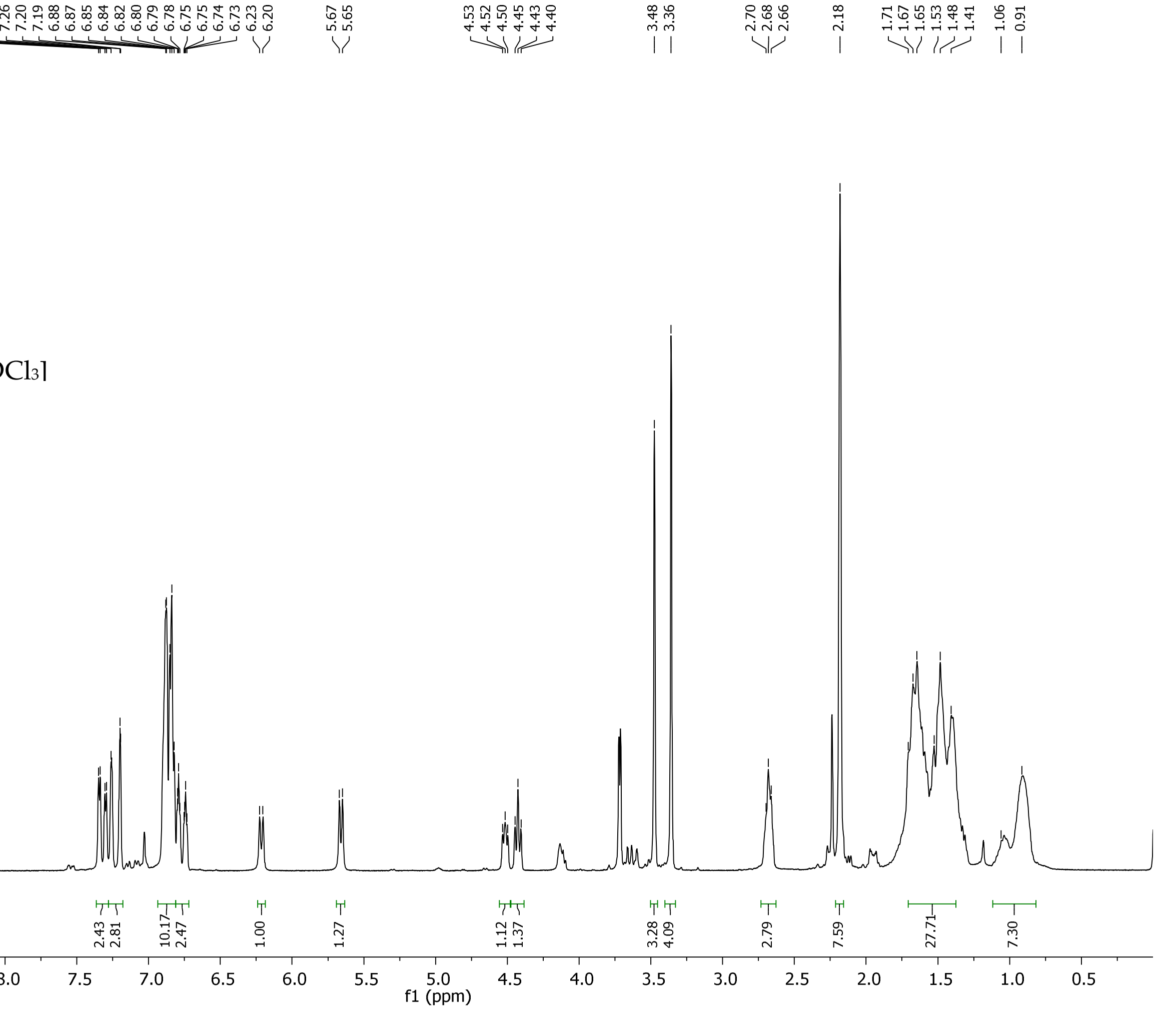

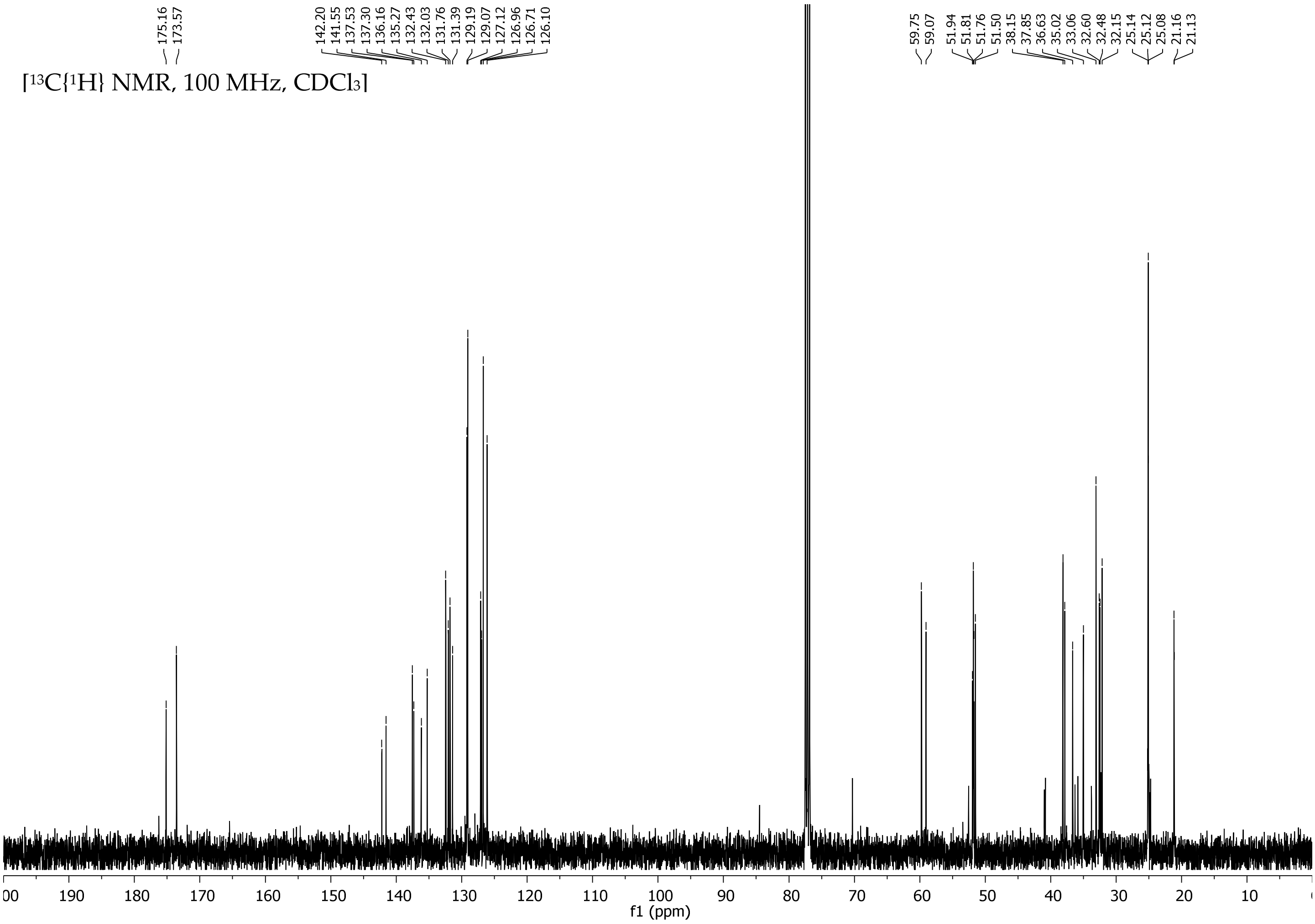
[13C{1H} NMR, 100 MHz, CDCl3]
175.16
173.57
142.20
141.55
137.53
137.30
136.16
135.27
132.43
132.03
131.76
131.39
129.19
129.07
127.12
126.96
126.71
126.10
59.75
59.07
51.94
51.81
51.76
51.50
38.15
37.85
36.63
35.02
33.06
32.60
32.48
32.15
25.14
25.12
25.08
21.16
21.13
f1 (ppm)

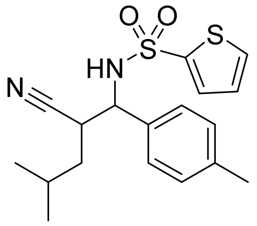

8cib
d.r. = 53:47
[¹H NMR, 400 MHz, CDCl₃]

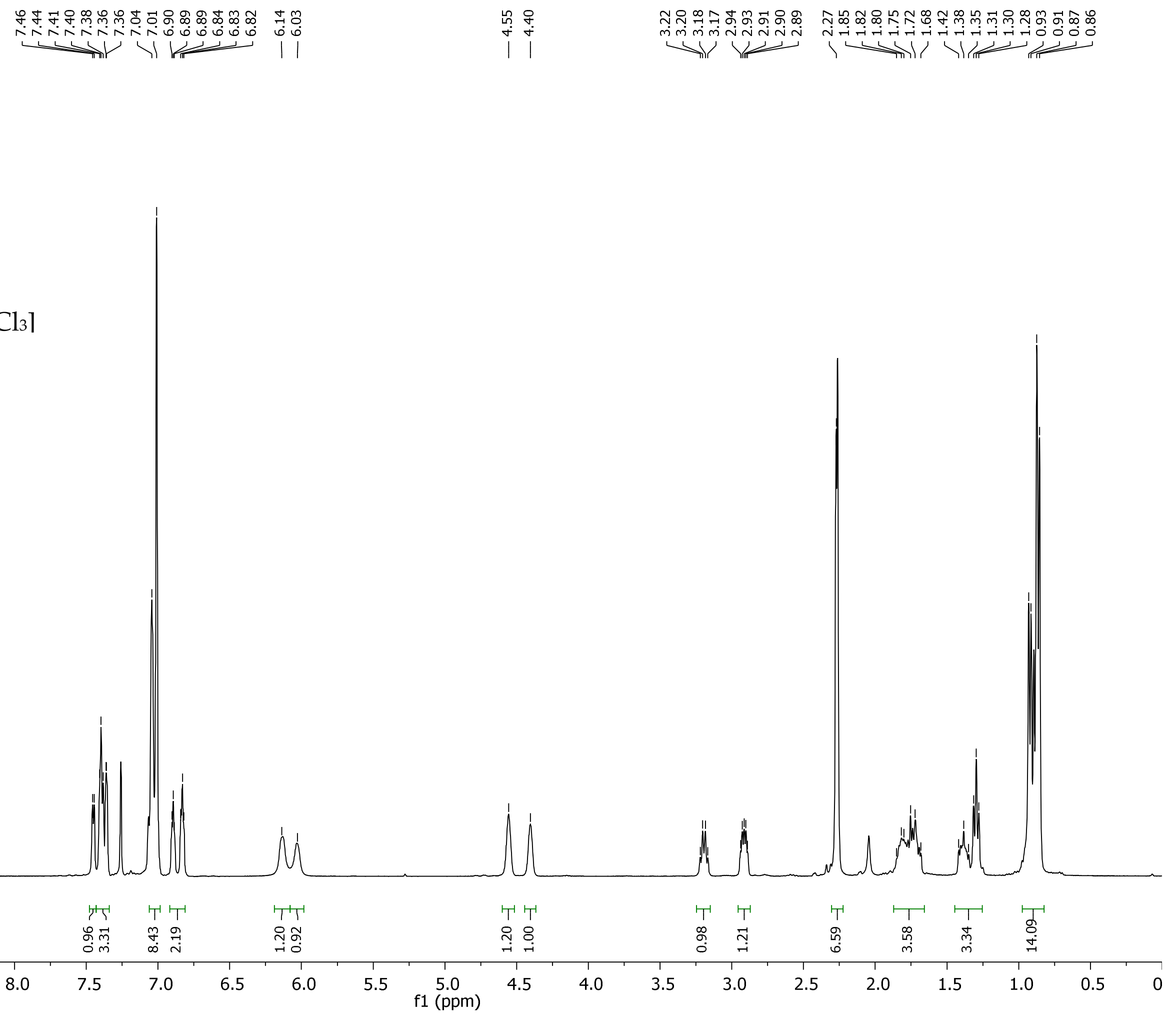

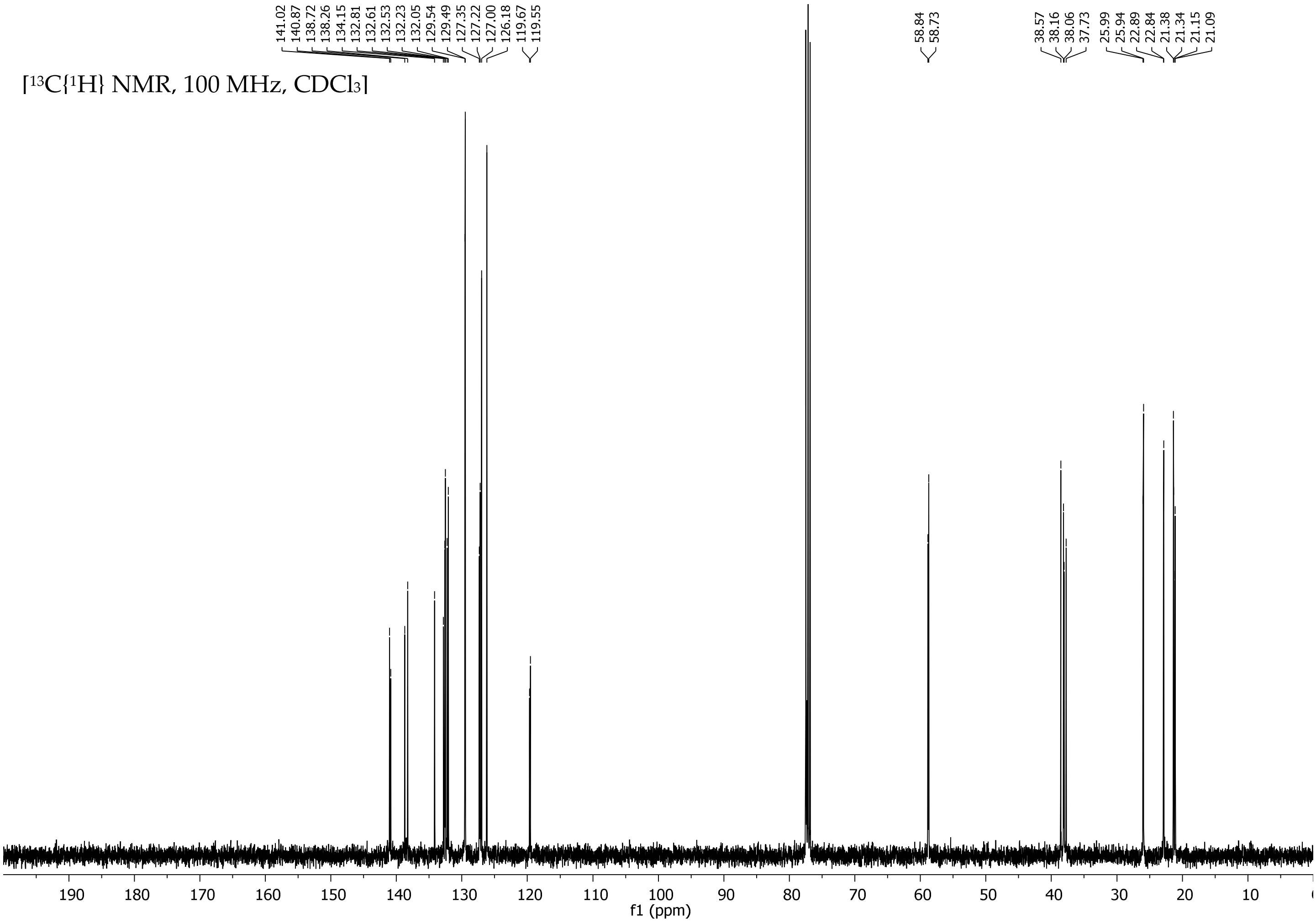
[13C{1H} NMR, 100 MHz, CDCl3]
141.02
140.87
138.72
138.26
134.15
132.81
132.61
132.53
132.23
132.05
129.54
129.49
127.35
127.22
127.00
126.18
119.67
119.55
58.84
58.73
38.57
38.16
38.06
37.73
25.99
25.94
22.89
22.84
21.38
21.34
21.15
21.09
190 180 170 160 150 140 130 120 110 100 90 80 70 60 50 40 30 20 10
f1 (ppm)

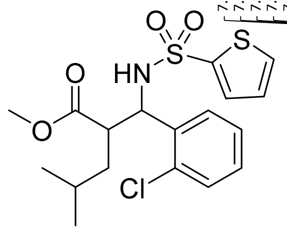

8cac
d.r. = 69:31

[^{1}H NMR, 400 MHz, $CDCl_3$]

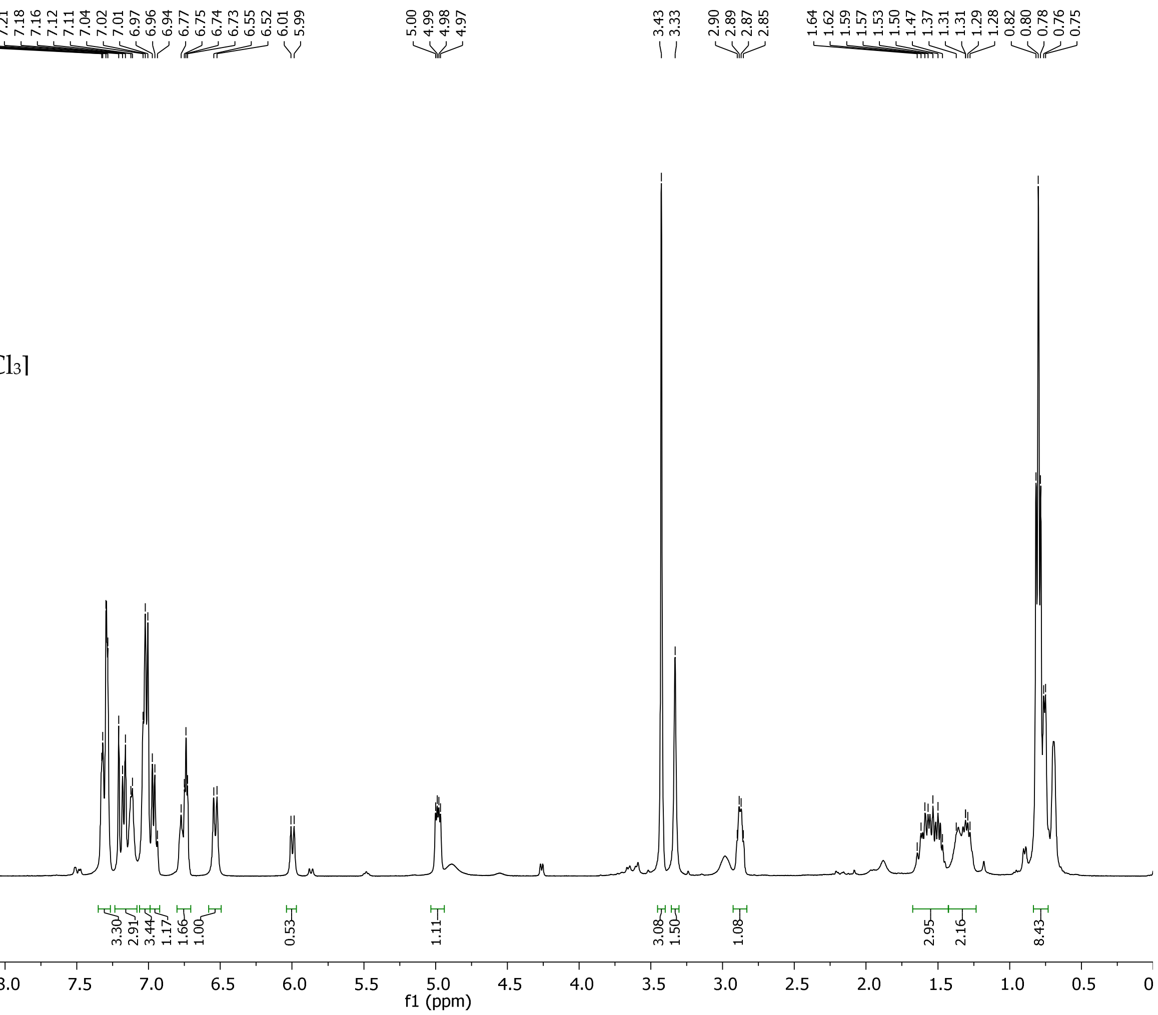

[13C{1H} NMR, 100 MHz, CDCl3]

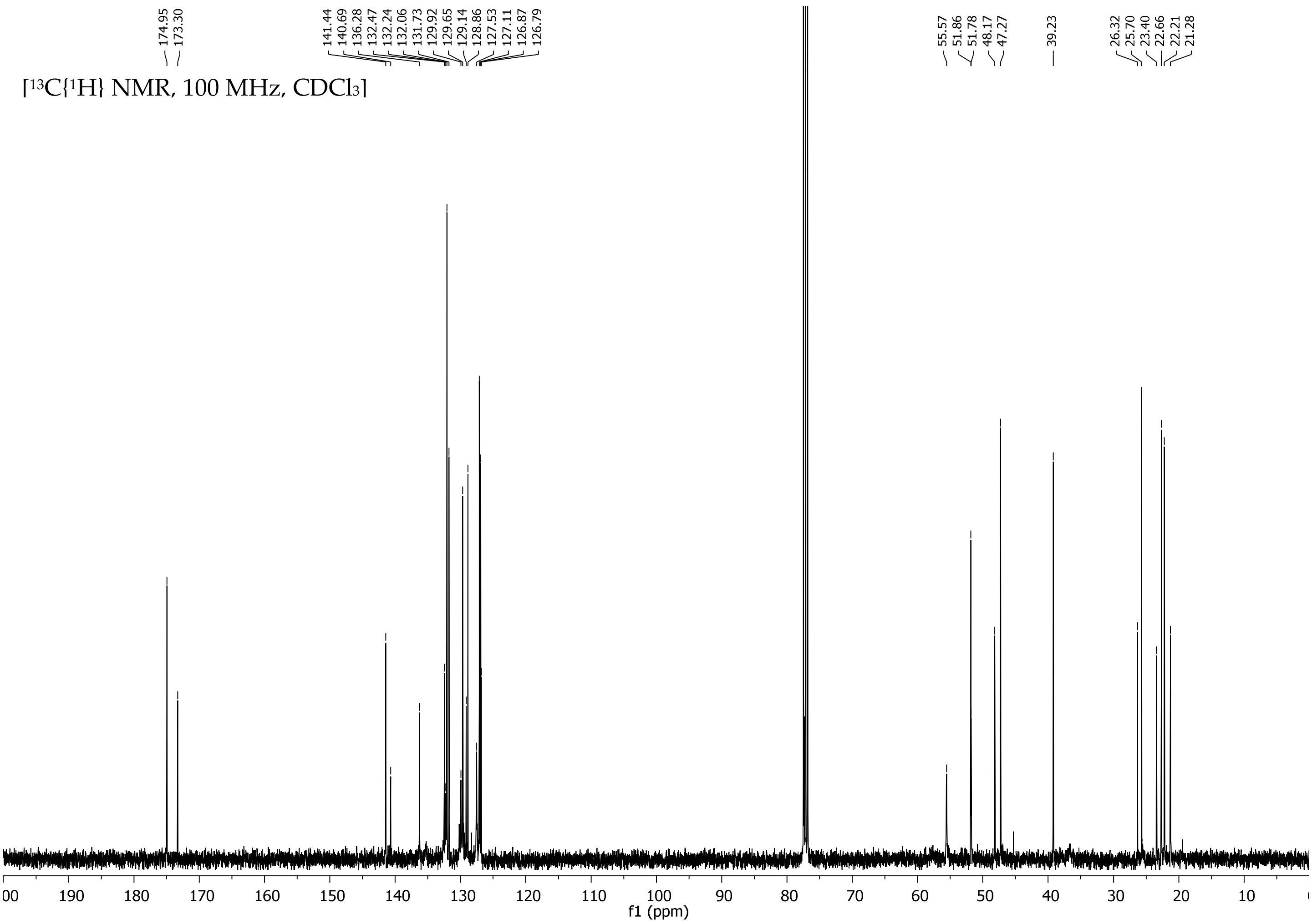

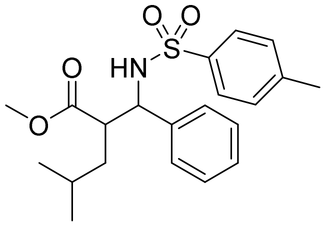

8cad
d.r. = 56:44

[¹H NMR, 400 MHz, CDCl3]

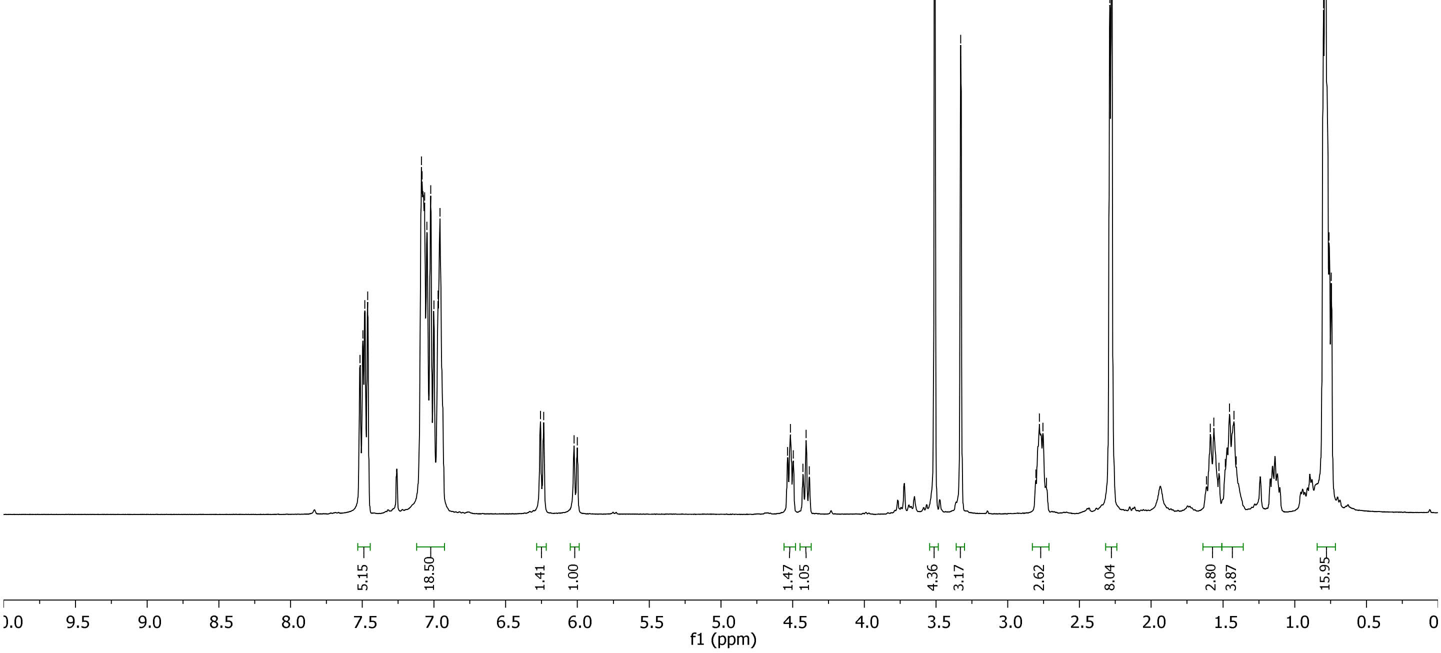

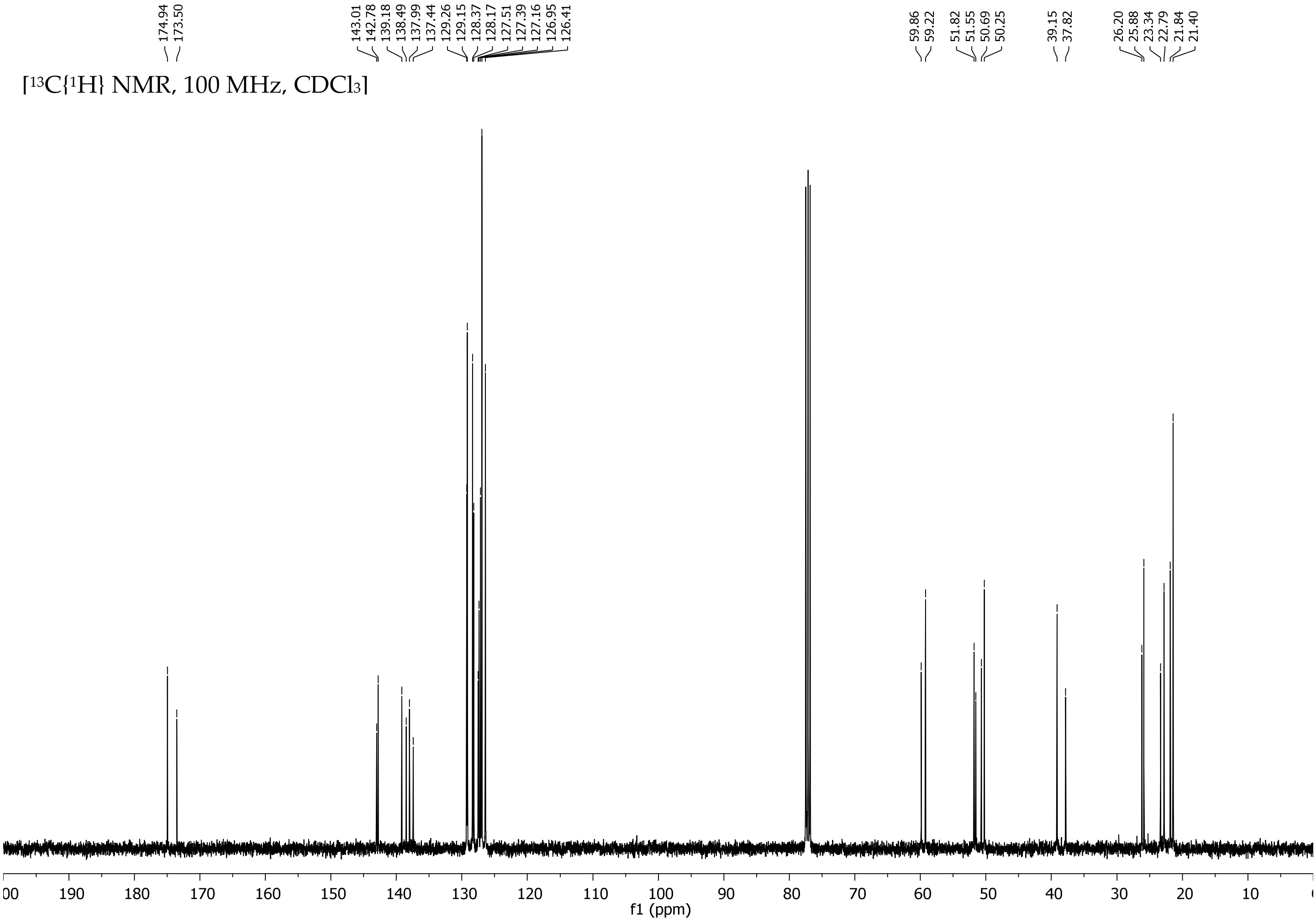
[13C{1H} NMR, 100 MHz, CDCl3]
174.94
173.50
143.01
142.78
139.18
138.49
137.99
137.44
129.26
129.15
128.37
128.17
127.51
127.39
127.16
126.95
126.41
59.86
59.22
51.82
51.55
50.69
50.25
39.15
37.82
26.20
25.88
23.34
22.79
21.84
21.40
f1 (ppm)

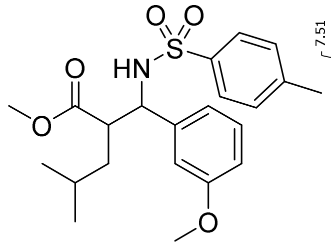

8cae
d.r. = 55:45

[^{1}H NMR, 400 MHz, $CDCl_3$]

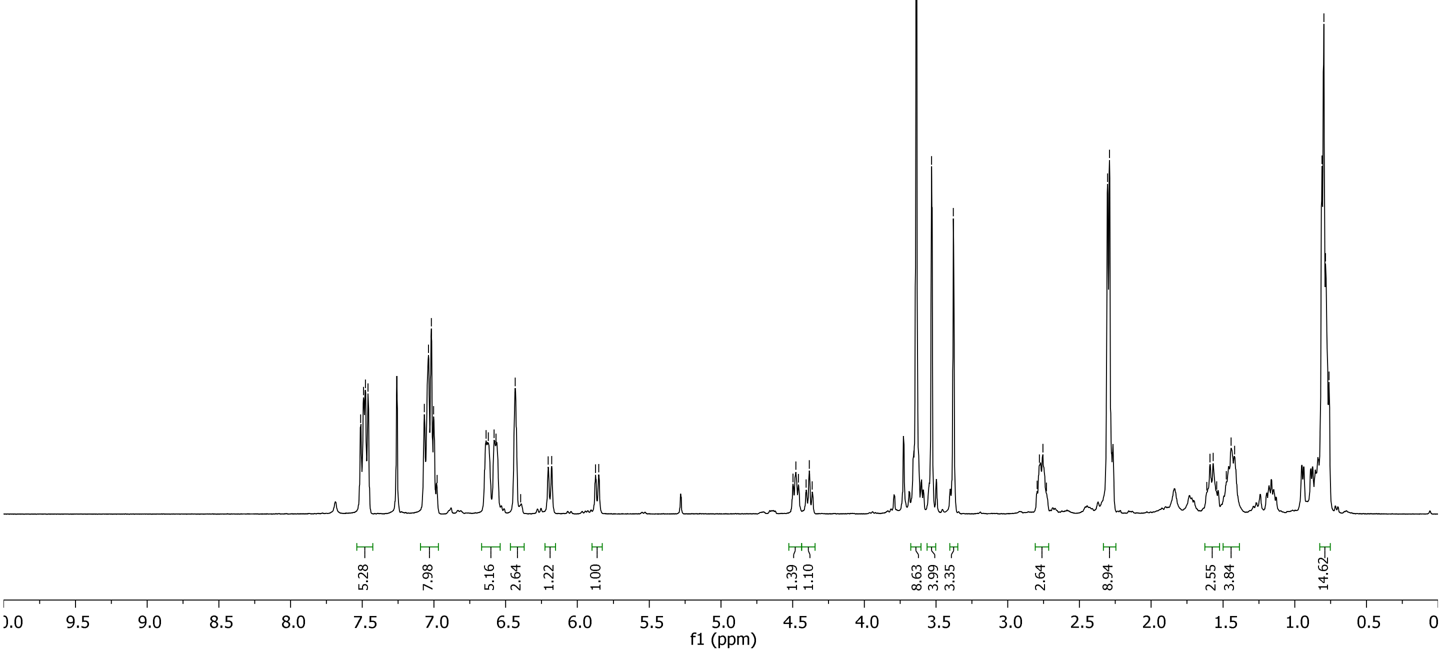

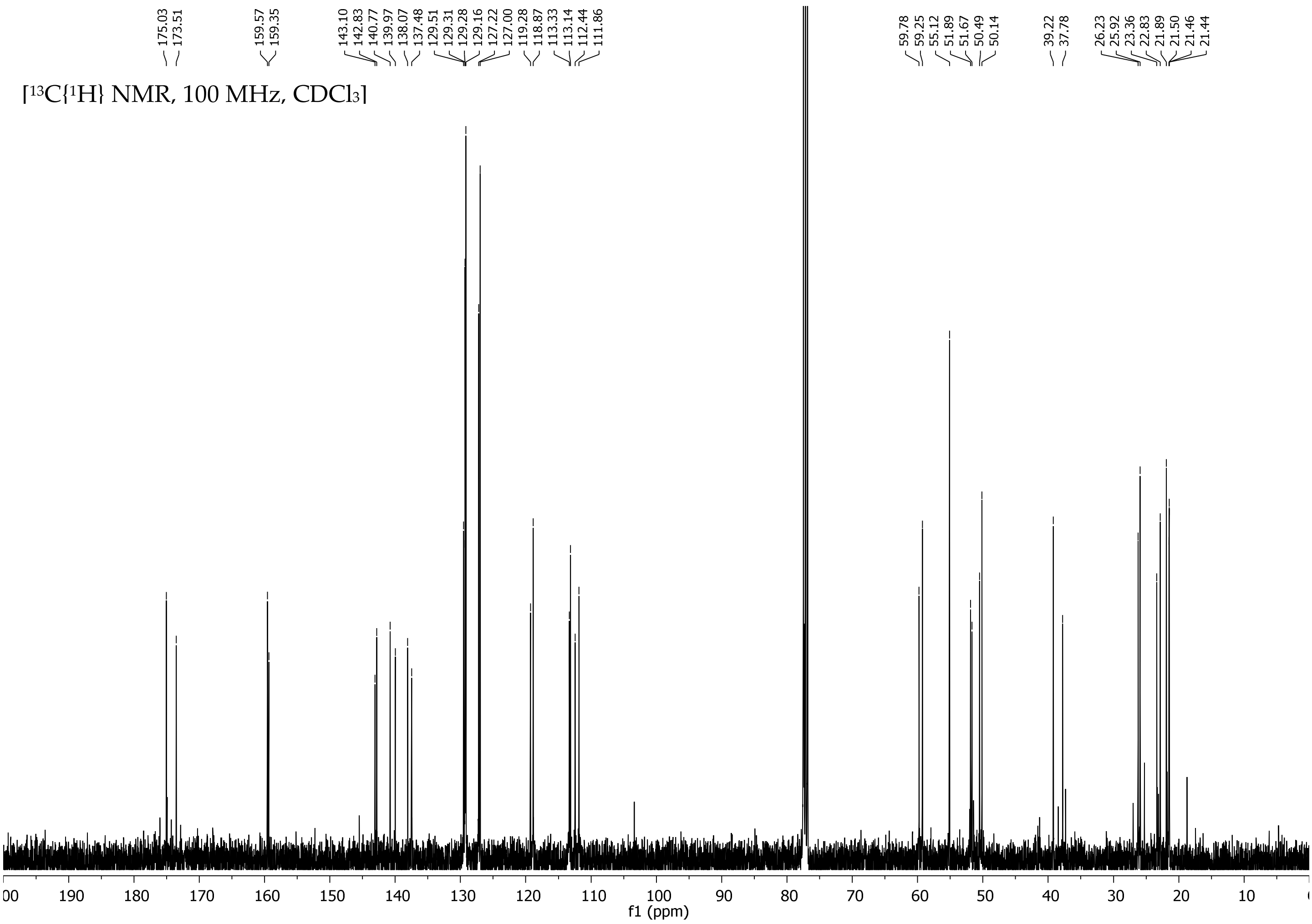
[13C{1H} NMR, 100 MHz, CDCl3]
175.03
173.51
159.57
159.35
143.10
142.83
140.77
139.97
138.07
137.48
129.51
129.31
129.28
129.16
127.22
127.00
119.28
118.87
113.33
113.14
112.44
111.86
59.78
59.25
55.12
51.89
51.67
50.49
50.14
39.22
37.78
26.23
25.92
23.36
22.83
21.89
21.50
21.46
21.44
f1 (ppm)
